# Dialogue mechanisms between astrocytic and neuronal networks: A whole-brain modelling approach

**DOI:** 10.1371/journal.pcbi.1012683

**Published:** 2025-01-13

**Authors:** Obaï Bin Ka’b Ali, Alexandre Vidal, Christophe Grova, Habib Benali

**Affiliations:** 1 Physics Department, Concordia University, Montreal, Canada; 2 Electrical and Computer Engineering Department, Concordia University, Montreal, Canada; 3 Laboratoire de Mathématiques et Modélisation d’Evry (LAMME), Université Evry, CNRS, Université Paris-Saclay, France; 4 Multimodal Functional Imaging Lab, Department of Physics, Concordia School of Health, Concordia University, Montreal, Canada; 5 Multimodal Functional Imaging Lab, Biomedical Engineering Department, McGill University, Montreal, Canada; 6 INSERM U1146, Paris, France; Inria, FRANCE

## Abstract

Astrocytes critically shape whole-brain structure and function by forming extensive gap junctional networks that intimately and actively interact with neurons. Despite their importance, existing computational models of whole-brain activity ignore the roles of astrocytes while primarily focusing on neurons. Addressing this oversight, we introduce a biophysical neural mass network model, designed to capture the dynamic interplay between astrocytes and neurons via glutamatergic and GABAergic transmission pathways. This network model proposes that neural dynamics are constrained by a two-layered structural network interconnecting both astrocytic and neuronal populations, allowing us to investigate astrocytes’ modulatory influences on whole-brain activity and emerging functional connectivity patterns. By developing a simulation methodology, informed by bifurcation and multilayer network theories, we demonstrate that the dialogue between astrocytic and neuronal networks manifests over fast–slow fluctuation mechanisms as well as through phase–amplitude connectivity processes. The findings from our research represent a significant leap forward in the modeling of glial-neuronal collaboration, promising deeper insights into their collaborative roles across health and disease states.

## Author summary

Our study explores astrocyte-neuron interactions within a novel computational model that simulates whole-brain dynamics across two interconnected structural network layers. Our model incorporates astrocytic connections, mimicking gap junctions, which enforce short-range coupling aligned with the brain’s geometry. Additionally, it includes neuronal constraints derived from axonal fiber organization, balancing the contributions of long-range and short-range neural connections. This model allows us to examine how a network of astrocytes regulates neurochemical pathways involving key neurotransmitters like glutamate and GABA, and how these regulations influence neuronal electrical activity. Our findings reveal that astrocytic influence on neuronal activity is essential for maintaining homeostasis through slow (less than 0.5 Hz), spatially structured changes in neurotransmitter levels. These changes are closely linked to amplitude modulation within the electrophysiological alpha rhythm (8–13 Hz), with biochemical and bioelectrical fluctuations mirroring each other. Moreover, astrocytic regulation clarifies the relationship between the coordination of neural signal amplitudes and the synchronization of neural phases. Overall, our study provides new insights into the interplay between biochemical and bioelectrical processes from a neuron-glial perspective, offering a deeper understanding of the brain’s complexity and the intricate mechanisms that underlie its functioning.

## Introduction

**Fig 1:**
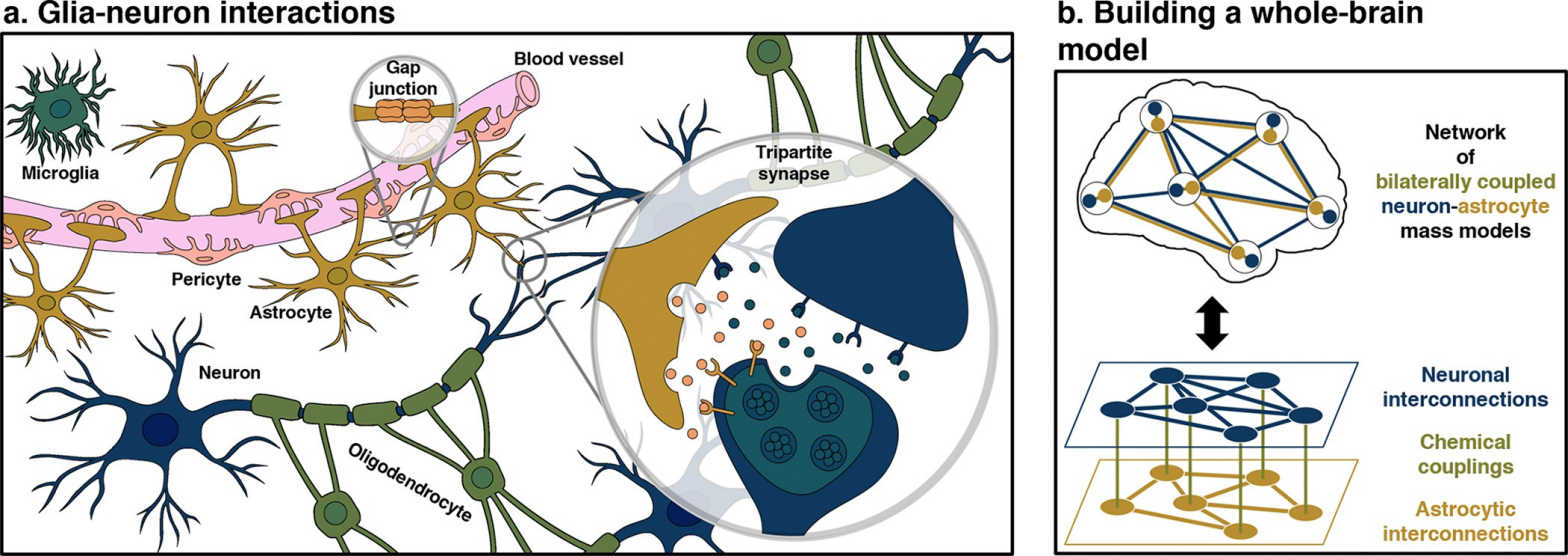
Glia-neuron modeling. **(a)** Illustrates astrocytes’ multifaceted roles in the brain, including synaptic and vascular contacts, brain tissue *tiling* into mini-circuits, and formation of a syncytium connected by gap junctions for intercellular communication. Although our study focuses solely on astrocyte-neuron interactions, this panel also provides context by highlighting the interactions of various glial cells, like oligodendrocytes (distinguished as they wrap myelin around axons to speed up neuronal transmission) and microglia (well-known to keep the brain under surveillance for damage or infection), with neurons, and with blood vessels through pericytes, contributing to the neuro-glio-vascular unit. **(b)** Introduces our biophysical model that simulates whole-brain activity by coupling astrocytic and neuronal networks through glutamatergic and GABAergic transmission pathways. The model is characterized by a two-layered structural network, where one layer interconnects astrocytic populations and the other interconnects neuronal populations, with both layers interacting at each node. For illustration, the schematic discretizes the brain into six nodes corresponding to regions such as the middle frontal, superior frontal, superior parietal, lateral occipital, middle temporal, and insula. This simplified schematic is used for conceptual clarity and to showcase key features such as first-neighbor astrocytic connections and the more complex neuronal connections, but does not capture the full scale or complexity of the model, which is described in detail in the “*Methods*” section. For example, in the astrocytic layer, where interconnections are restricted to first-neighbor connections aligned with the cortical surface’s geometry, the middle frontal node connects only to its immediate neighbors, such as the superior frontal and insula nodes, but not to more *distant* regions like the parietal or temporal lobes. In contrast, the neuronal layer balances short-range and long-range connections according to axonal fiber organization. With the use of a specific brain parcellation, the model accurately distinguishes between first-neighbor and more distant connections, ensuring their precise representation.

Astrocytes are intricately intertwined with neurons, both structurally and functionally [1,2]. Central to this coupling is the tripartite synapse model (Fig 1A), within which astrocytes employ various mechanisms (such as membrane channels, receptors, transporters, and pumps) to not only monitor and regulate synaptically released substances by neurons (including ions, neurotransmitters, neurotrophic factors) but also engage and partake in signaling, e.g., by releasing gliotransmitters like glutamate and gamma-aminobutyric acid (GABA) back to presynaptic and postsynaptic neuronal terminals [1,2]. Moreover, astrocytes generally delimit non-overlapping or minimally overlapping territories (Fig 1A), with each territory encompassing 0.3–2 million synapses that may be associated with multiple neurons [2,3]. Additionally, astrocytes interconnect through gap junctions (Fig 1A), forming syncytia that support intercellular communication via propagating calcium waves [4,5]. Other key roles of astrocytes encompass ion homeostasis, metabolic and neurotrophic supports, inflammation, blood-brain barrier maintenance and function, neurovascular coupling, cellular interactions beyond neurons and synapses to involve microglia, oligodendrocytes, and pericytes [1,2,5,6]. Collectively, these examples underscore astrocytes’ capacity to influence neuronal circuits actively, strategically, and profoundly [1,2]. The intimate association between astrocytes and neurons also suggests that they operate as a unified functional entity rather than as independent units [1,2].

Yet, despite their evident significance as potential signaling hubs within the neuropil, astrocytes have received limited attention in neurobiological research compared to neurons [1,2,5,6]. Inevitably, the prevailing focus on neurons has led to a limited understanding of the computational processes in the brain [1]. To address this discrepancy, the field of neuroscience has recently seen a momentous shift towards a neuron-glial perspective. This evolving viewpoint encourages neuroscientists to reevaluate existing paradigms and theories to include glial cells [1]. Complementing this neuron-glial perspective are significant advancements in the field of chemical neuromodulation [7,8]. Historically developing alongside glial research, yet somewhat independently, this field has also come to advocate for a comprehensive approach beyond neuronal wiring and firing, that acknowledges the intricacies of the brain’s neurochemical environment.

The neuron-glial perspective aspires to elucidate the mutual dependence between neuronal and glial processes across a spectrum of spatiotemporal scales, from molecular to system-wide dynamics and from milliseconds to years [2,6]. It calls for a conceptual framework wherein the brain is characterized not only by the interactions between neurons and glia but also by the flexible integration of glial processes into the architectural and operational fabric of neuronal circuits. In practice, because neuron-glial interactions are inherently nonlinear and multiscale, computational modeling becomes an essential tool to comprehend them [1,5]. However, the latest reviews reveal a conspicuous scarcity of modeling efforts beyond microscale phenomena, with a marked absence of models addressing whole-brain scales [1,5]. We aim to fill this void.

**Fig 2:**
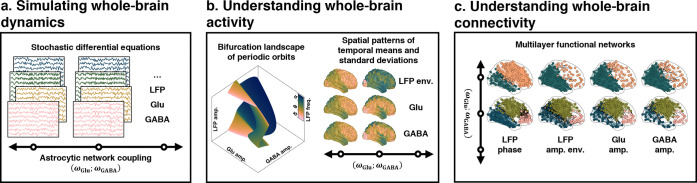
Study overview. **(a)** Describes our simulation approach of systematically varying two global parameters, *ω*_Glu_ and *ω*_GABA_, to explore the astrocytic network’s modulation of neurotransmission. We defined 1225 unique parameter pairs from physiologically plausible criteria and conducted stochastic simulations for each, using a network of 216 nodes, with each simulation lasting 120 seconds and repeated ten times. **(b)** Shows the methodology for analyzing network activity, including the derivation of whole-brain metrics, and regional temporal means and standard deviations. Bifurcation analysis, focusing on amplitude and frequency properties of periodic orbits, was employed to gain deeper biophysical insights into the oscillatory network dynamics. **(c)** Outlines the construction of a four-layered multiplex functional network for analyzing connectivity, with layers capturing different aspects of neural communication, including phase locking values and amplitude envelope Pearson-correlations of alpha-band-limited LFP dynamics, and Pearson-correlations of Glu_e_ or GABA_e_ dynamics. The analysis consisted in characterizing network properties such as clustering coefficient, path length, edge overlap, eigenvector versatility, community organization, and structural reducibility.

In this study, we unveil a biophysical model that captures the large-scale activity of neuron-astrocyte networks (Fig 1B). The model strikes a balance between biological fidelity and computational feasibility by leveraging principles from neural mass network modeling and compartmental modeling [9,10]. Additionally, it is tailored to the spatiotemporal dimensions pertinent to human neuroimaging data for functional studies [9,10]. The model represents each node within the network as a mesoscopic brain region whose temporal activity is explained by a neuron-astrocyte mass model (Fig 1B). This regional activity is driven by interactions between neuronal and astrocytic populations through glutamatergic and GABAergic transmissions, modulated by stochastic fluctuations and the influence of distal regions. Central to the model is a two-layered structural network to constrain the distal influences (Fig 1B): one layer connects neuronal populations across regions, reflecting white matter tracts, while the other interconnects astrocytic populations, symbolizing gap junctional densities. This coupling framework facilitates the exploration of how regional dynamics of neurotransmission can prompt adjacent astrocytic populations to synchronize their activities, following a unique topological arrangement distinct from the neuronal layer. Concurrently, employing this network scheme allows delving into the astrocytic networks’ role in modulating whole-brain neuronal firing patterns via gliotransmission. In this study, we theoretically investigate the contributions of astrocytic networks to the patterns of whole-brain activity and emerging functional connectivity, through physiologically constrained simulations complemented by bifurcation and multilayer network analyses (Fig 2).

### Model

This section is divided into three subsections, each delving into the core principles underpinning the mutual coupling between astrocytic and neuronal networks, which form the foundation of our whole-brain model depicted in Fig 1B. In our model, each node encapsulates a mesoscopic brain region represented through a mass model, detailed in the “*Neuron-astrocyte mass model*” subsection. These nodes structurally interconnect through a two-layered network architecture, distinguishing between neuronal and astrocytic pathways. The “*Network extension for the neuronal compartment*” subsection elucidates the neuronal network coupling, while the “*Network extension for the astrocytic compartment*” subsection describes the astrocytic network coupling.

In the following subsections, temporal derivatives are indicated by overdots. Additionally, we adopt the common practice of setting most model parameters homogeneously across all network nodes [9,10], although it is possible in a more detailed model for any given parameter to vary by region and over time. *Tables* in *S1 File* provide a comprehensive overview of the symbols used to represent each variable and parameter introduced.

### Neuron-astrocyte mass model

This mass model characterizes the coarse-grained temporal dynamics among four distinct, yet coupled, homogeneous subpopulations of neural cells: glutamatergic pyramidal neurons (Pyr), excitatory interneurons (ExIn; a term used here to encompass both pyramidal neurons that are distinct from the primary Pyr population in their laminar position, as well as spiny stellate cells found in certain sensory cortices), GABAergic inhibitory interneurons (InIn), and astrocytes (Ast). It extends the foundational work presented in reference [11], by offering a nuanced portrayal of the interplay between neuronal and astrocytic subpopulations through electrical firing as well as glutamatergic and GABAergic transmission pathways. This extension critically assumes that astrocytic populations exhibit sufficient functional homogeneity to be biologically plausibly modeled within the mass modeling framework [5]. Notably, our enhanced neuron-astrocyte mass model incorporates the effects of glutamatergic gliotransmission as well as stochastic fluctuations arising from both distant regions and the immediate nodal environments.

At the nodal level, the mass model, indexed by *n*, articulates two primary types of interactions among the subpopulations: neuron-neuron and neuron-astrocyte.

On the one hand, neuron-neuron interactions are abstracted to the dendro-somatic transformations between subpopulation average postsynaptic potentials (*E*_Pyr_, *E*_ExIn_, and *E*_InIn_) and mean firing rates (*F*_Pyr_, *F*_ExIn_, and *F*_InIn_); assuming that feedforward pyramidal neurons receive feedback from excitatory and inhibitory interneurons, excitatory neuronal network feedback (*Q*_Pyr_), and arbitrary excitatory inputs (*q*). These interactions are formalized in Eqs *(1)* and *(2)*, while the neuronal network feedback term is explored in more detail in the subsection “*Network extension for the neuronal compartment*”. For simplicity, and in contrast to reference [11], we omitted the excitatory self-feedback mechanism on the pyramidal neurons.

*Postsynaptic potential dynamics*:

$${{\ddot{E}}_{Pyr}}_{\left[n\right]}=Aa{F_{Pyr}}_{\left[n\right]}-2a{{\dot{E}}_{Pyr}}_{\left[n\right]}-a^{2}{E_{Pyr}}_{\left[n\right]}$$

$${{\ddot{E}}_{ExIn}}_{\left[n\right]}=Aa\left(C^{ExIn\to Pyr}{F_{ExIn}}_{\left[n\right]}+\underset{\begin{array}{c} neuroal\\ network\\ feedback \end{array}}{\underset{︸}{{Q_{Pyr}}_{\left[n\right]}}}+q_{\left[n\right]}\right)-2a{{\dot{E}}_{ExIn}}_{\left[n\right]}-a^{2}{E_{ExIn}}_{\left[n\right]}$$ (1)

$${{\ddot{E}}_{InIn}}_{\left[n\right]}=BbC^{InIn\to Pyr}{F_{InIn}}_{\left[n\right]}-2b{{\dot{E}}_{InIn}}_{\left[n\right]}-b^{2}{E_{InIn}}_{\left[n\right]}$$

*Neuronal firing rates*:

$${F_{Pyr}}_{\left[n\right]}=S({E_{ExIn}}_{\left[n\right]}-{E_{InIn}}_{\left[n\right]},\nu_{max},r,{v_{Pyr}}_{\left[n\right]},0)$$

$${F_{ExIn}}_{\left[n\right]}=S(C^{Pyr\to ExIn}{E_{Pyr}}_{\left[n\right]},\nu_{max},r,{v_{ExIn}}_{\left[n\right]},0)$$ (2)

$${F_{InIn}}_{\left[n\right]}=S(C^{Pyr\to InIn}{E_{Pyr}}_{\left[n\right]},\nu_{max},r,{v_{InIn}}_{\left[n\right]},0)$$

Here S is a *sigmoidal function* defined as:

S:(x,ν,r,θ,δ)⟼ν/(1+exp(r(θ−x)))−δ

On the other hand, neuron-astrocyte interactions are modeled as concurrent synaptic releases and uptakes of neurotransmitters into and from the extracellular space (e), as described in [12]. The model specifically considers the two major neurotransmitters: glutamate (Glu) and GABA, for excitatory and inhibitory signaling, respectively, as detailed in Eqs *(3)* and *(4)*.

*Glutamate dynamics*:

$${{\ddot{J}}_{\mathrm{Glu}}}_{\left[n\right]}=Ww_{r}\left(\underset{\begin{array}{c} neuronal\;firing\\ induced\;Glu\;release \end{array}}{\underset{︸}{{F_{Pyr}}_{\left[n\right]}}}+\underset{\begin{array}{c} astrocytic\;network\\ induced\;Glu\;release \end{array}}{\underset{︸}{{Q_{Glu}^{Ast}}_{\left[n\right]}}}\right)-(w_{r}+w_{d}){{\dot{J}}_{Glu}}_{\left[n\right]}-w_{r}w_{d}{J_{Glu}}_{\left[n\right]}$$

$${{\dot{Glu}}_{e}}_{\left[n\right]}={J_{Glu}}_{\left[n\right]}-\underset{astrocytic\;Glu\;uptake}{\underset{︸}{S({{Glu}_{e}}_{\left[n\right]},V_{Glu}^{e\to Ast},r_{Glu}^{e\to Ast,Pyr},\theta_{Glu}^{e\to Ast,Pyr},0)}}-\underset{neuronal\;Glu\;uptake}{\underset{︸}{S({{Glu}_{e}}_{\left[n\right]},V_{Glu}^{e\to Pyr},r_{Glu}^{e\to Ast,Pyr},\theta_{Glu}^{e\to Ast,Pyr},0)}}$$ (3)

$${{\dot{Glu}}_{Ast}}_{\left[n\right]}=S({{Glu}_{e}}_{\left[n\right]},V_{Glu}^{e\to Ast},r_{Glu}^{e\to Ast,Pyr},\theta_{Glu}^{e\to Ast,Pyr},0)-\underset{Glu\;degradation}{\underset{︸}{{{Glu}_{Ast}}_{\left[n\right]}/\tau_{Glu}^{Ast}}}$$

Glutamate release (*J*_Glu_) is modulated by the firing activity of pyramidal neurons (*F*_Pyr_), while GABA release (*J*_GABA_) is controlled by the activity of inhibitory interneurons (*F*_InIn_). These release dynamics are also regulated by the astrocytic network, specifically through $Q_{Glu}^{Ast}$ and $Q_{GABA}^{Ast}$, a topic elaborated upon in the subsection “*Network extension for the astrocytic compartment*”. The uptake of extracellular glutamate (Glu_e_) is primarily astrocytic, though neurons contribute to a lesser extent (see also *Table B* in *S1 File*). In contrast, the uptake of extracellular GABA (GABA_e_) is primarily neuronal, with astrocytes playing a subsidiary role (refer also to *Table B* in *S1 File*). Post-uptake, neurotransmitters are degraded within astrocytes, as captured by the state variables Glu_Ast_ and GABA_Ast_ [12].

*GABA dynamics*:

$${{\ddot{J}}_{GABA}}_{\left[n\right]}=Zz_{r}\left(\underset{\begin{array}{c} neuronal\;firing\\ induced\;GABA\;release \end{array}}{\underset{︸}{{F_{InIn}}_{\left[n\right]}}}+\underset{\begin{array}{c} astrocytic\;network\\ induced\;GABA\;release \end{array}}{\underset{︸}{{Q_{GABA}^{Ast}}_{\left[n\right]}}}\right)-(z_{r}+z_{d}){{\dot{J}}_{GABA}}_{\left[n\right]}-z_{r}z_{d}{J_{GABA}}_{\left[n\right]}$$

$${{\dot{GABA}}_{e}}_{\left[n\right]}={J_{GABA}}_{\left[n\right]}-\underset{astrocytic\;GABA\;uptake}{\underset{︸}{H({{GABA}_{e}}_{\left[n\right]},V_{GABA}^{e\to Ast},K_{GABA}^{e\to Ast})}}-\underset{neuronal\;GABA\;uptake}{\underset{︸}{H({{GABA}_{e}}_{\left[n\right]},V_{GABA}^{e\to InIn},K_{GABA}^{e\to InIn})}}$$ (4)

$${{\dot{GABA}}_{Ast}}_{\left[n\right]}=H({{GABA}_{e}}_{\left[n\right]},V_{GABA}^{e\to Ast},K_{GABA}^{e\to Ast})-\underset{GABA\;degradation}{\underset{︸}{{{GABA}_{Ast}}_{\left[n\right]}/\tau_{GABA}^{Ast}}}$$

Here H is a *Michaelis–Menten function* defined as:

H:(x,V,K)⟼Vx/(K+x)

Critically, the mass model establishes a relationship between extracellular neurotransmitter concentrations and neuronal firing rates through the excitability levels of targeted neuronal subpopulations, as formulated in Eq *(5)*. This relationship manifests in two ways: an elevation in Glu_e_ generally leads to a bounded (potentially transient) decrease in the excitability thresholds of both pyramidal cells and inhibitory interneurons, while an elevation in GABA_e_ typically results in a bounded (potentially transient) increase in the excitability threshold of pyramidal neurons. Focusing on excitability thresholds allows the mass model to effectively capture the immediate, dynamic effects of extracellular neurotransmitter levels on these neuronal subpopulations. This approach specifically targets short-term regulatory processes, without conflating these effects with the more stable, long-term synaptic changes associated with phenomena like long-term potentiation and long-term depression. This distinction is crucial for understanding the rapid, transient interactions between astrocytes and neurons, which are central to our study of spontaneous whole-brain activity. For simplicity, the model assumes that excitatory interneurons remain unaffected by changes in extracellular neurotransmitter levels [11].

Given the concurrent nature of all the nodal processes described so far, complex interactions emerge between neuronal excitatory and inhibitory firings, and neuron-astrocyte uptakes and releases of neurotransmitters, fostering a wide repertoire of dynamics across various (fast–slow) timescales [11,12].

*Neuronal excitability levels*:

$${v_{Pyr}}_{\left[n\right]}=v_{0}+{v_{GABA}}_{\left[n\right]}-{v_{Glu}}_{\left[n\right]}$$

$${v_{ExIn}}_{\left[n\right]}=v_{0}$$

$${v_{InIn}}_{\left[n\right]}=v_{0}-\mu_{Glu}^{InIn/Pyr}{v_{Glu}}_{\left[n\right]}$$ (5)

$${v_{Glu}}_{\left[n\right]}=S({{Glu}_{e}}_{\left[n\right]},m_{Glu}^{Pyr},r_{Glu}^{Pyr,InIn},\theta_{Glu}^{Pyr,InIn},\delta_{Glu}^{Pyr})$$

$${v_{GABA}}_{\left[n\right]}=S({{GABA}_{e}}_{\left[n\right]},m_{GABA}^{Pyr},r_{GABA}^{Pyr},\theta_{GABA}^{Pyr},\delta_{GABA}^{Pyr})$$

### Network extension for the neuronal compartment

Consistent with established practices [9,10], neuronal interconnections across the network’s nodes via white matter tracts are presumed to be excitatory, involving solely the pyramidal cell populations. The corresponding network interaction terms for these connections are detailed in Eq *(6)* and appear in the state variable *E*_ExIn_ in Eq *(1)*. These terms are formulated as a linear combination of incoming firing rates (*Q*_Pyr_), with the weights encapsulated in the parameter matrix *Ω*_Pyr_, and a global coupling parameter, *ω*_Pyr_, modulates the relative impact of *Ω*_Pyr_ on nodal dynamics. The detailed methodology to define the matrix *Ω*_Pyr_ from empirical diffusion magnetic resonance imaging (MRI) data is provided in the section “*Defining structural layers*” of “*Methods*”.

*Neuronal network feedback*:

$${Q_{Pyr}}_{\left[n\right]}=\omega_{Pyr}\sum_{\tilde{n}}{\Omega_{Pyr}}_{\left[n,\tilde{n}\right]}{F_{Pyr}}_{\left[\tilde{n}\right]}$$ (6)

### Network extension for the astrocytic compartment

Due to the current lack of comprehensive experimental data for whole-brain astrocytic modeling, our network model incorporates insights from various microscale studies. These studies have demonstrated and clarified the activities within astrocytic networks that interconnect through gap junctions [1–4,6,13,14]. For instance, the work referenced in [13] describes a process where a portion of glutamate synaptically released by a neuron into the extracellular space can bind to the glutamate receptors of an astrocyte. This binding may initiate a cascade of events, including the production of inositol 1,4,5-trisphosphate within the astrocyte. This compound can trigger calcium release from the endoplasmic reticulum within the same astrocyte and propagate through gap junctions to stimulate calcium release in adjacent astrocytes. Subsequently, the calcium releases may lead these astrocytes to secrete glutamate into the extracellular space, which can diffuse extrasynaptically and bind to pre-terminal neuronal receptors, potentially inducing neurotransmitter release independently of neuronal firing.

Building on these insights, we have developed a preliminary astrocytic network coupling model. This coupling model extrapolates from the structural concept of astrocytes forming a syncytium connected by gap junctions, and from the functional roles of glutamate neurotransmission in facilitating intercommunication between astrocytes, as well as the impact of excitatory gliotransmission on neuronal pre-terminal receptors [3,4,6].

Structurally, the coupling model posits that astrocytic interconnections across network nodes adhere to a syncytial organization, where an astrocytic population within one region connects exclusively with those in immediately adjacent regions along the cortical mantle. To define this lattice-like network structure, we introduce the parameter matrix *Ω*_Ast_, which uses physical proximity as a surrogate for astrocytic coupling facilitated by gap junctional densities. This modeling approach is designed to simulate the attenuation of intercellular signaling that occurs over distance via gap junctions. The weights within *Ω*_Ast_ are determined by the reciprocal of the geodesic distances between the centers of mass of the regions, informed by the brain’s cortical surface geometry. The detailed methodology for constructing the parameter matrix *Ω*_Ast_ from empirical MRI data is elaborated in the “*Defining structural layers*” section within “*Methods*”. Given the novelty of this framework, to our knowledge, there are no established references for deriving a whole-brain astrocytic connectivity matrix from MRI data [1,3,15]. Our use of the reciprocal of geodesic distances between brain regions represents a new approach, providing a biologically plausible approximation of the pathways through which populations of astrocytes interact, as well as the attenuation of intercellular signaling over these pathways across the cortical surface.

*Astrocytic network feedback*:

$${Q_{Glu}^{Ast}}_{\left[n\right]}=\omega_{Glu}\sum_{\tilde{n}}{\Omega_{Ast}}_{\left[n,\tilde{n}\right]}S({{Glu}_{e}}_{\left[\tilde{n}\right]},m_{Glu}^{Ast},r_{Glu}^{Ast},\theta_{Glu}^{Ast},0)$$ (7)

$${Q_{GABA}^{Ast}}_{\left[n\right]}=\omega_{GABA}\sum_{\tilde{n}}{\Omega_{Ast}}_{\left[n,\tilde{n}\right]}S({{Glu}_{e}}_{\left[\tilde{n}\right]},m_{Glu}^{Ast},r_{Glu}^{Ast},\theta_{Glu}^{Ast},0)$$

Functionally, the coupling model proposes that the astrocytic network feedback ($Q_{Glu}^{Ast}$ and $Q_{GABA}^{Ast}$), as outlined in Eq *(7)*, partially modulate the nodal releases of neuronal glutamate and GABA (*J*_Glu_ and *J*_GABA_) through excitatory gliotransmission initiated by nodal glutamate bindings. For simplicity, we assume that astrocytic glutamate binding and uptake share similar sigmoidal kinetics, allowing their sigmoidal parameters to be equated. This modeling choice implies that elevated glutamate levels can intensify astrocytic coupling and network feedback. To express how the nodal neurotransmitter release rates (*J*_Glu_ and *J*_GABA_) are modulated, we incorporate linear terms in Eqs *(3)* and *(4)*, combining local (firing-induced) and distal (astrocytic-network-induced) dynamics, with the latter structurally constrained by *Ω*_Ast_. In these equations, the coupling parameters *ω*_Glu_ and *ω*_GABA_ are introduced to differentiate the astrocytic network’s impact on glutamate release by pyramidal cells versus GABA release by inhibitory interneurons, and they dictate the relative influence of *Ω*_Ast_ on nodal dynamics. This overall approach simulates the diffusion-like influence of distal extracellular glutamate concentrations on local neurotransmitter releases. We further simplify by assuming that astrocytic glutamate release into the extrasynaptic cleft, triggered by local glutamate binding, is comparatively less influential than the effects induced by neighboring astrocytes, thereby maintaining a zero diagonal in *Ω*_Ast_. By omitting astrocytic self-feedback mechanisms, we can focus more precisely on network behaviors, offering new insights into how inter-astrocytic and astrocyte-neuron communications orchestrate whole-brain dynamics. Lastly, as the literature provides less evidence for astrocytic network feedback mediated by GABA [14,16,17], we exclude the considerations of GABA-induced gliotransmission in our current model.

To summarize, the modulation of nodal neuronal glutamate and GABA release rates (*J*_Glu_ and *J*_GABA_) by the astrocytic network is represented by linear interaction terms ($Q_{Glu}^{Ast}$ and $Q_{GABA}^{Ast}$), with weights determined by the parameter matrix *Ω*_Ast_. These terms, detailed in Eq *(7)*, influence the state variables *J*_Glu_ and *J*_GABA_ in Eqs *(3)* and *(4)*, respectively. Additionally, two global coupling parameters, *ω*_Glu_ and *ω*_GABA_, govern the impact of *Ω*_Ast_ on nodal dynamics.

In essence, this postulated coupling model outlines a large-scale neuron-astrocyte network framework where regional glutamate dynamics encourage neighboring astrocytic populations to synchronize their activities. This synchronization is based on a topology symbolizing gap junctional densities, which is distinct from the neuronal topology determined by axonal densities (Fig 1B). This coordinated astrocytic network activity ultimately influences whole-brain patterns of neuronal excitatory and inhibitory firing rates through gliotransmission.

## Results

### Analyses overview

Our research aimed to elucidate the contributions of astrocytic networks to the patterns of whole-brain activity and functional connectivity. This investigation involved the systematic manipulation of two key global parameters, *ω*_Glu_ and *ω*_GABA_, which regulate the astrocytic network modulation of glutamatergic and GABAergic neurotransmissions, respectively (Fig 2A). We established an exploration grid for these parameters, guided by the objective of aligning our model’s outputs—such as the local field potential (LFP = *E*_ExIn_−*E*_InIn_), and the extracellular concentrations of glutamate (Glu_e_) and GABA (GABA_e_)—with empirically observed characteristics of *normative* resting-state dynamics in humans. Specifically, we sought to ensure that the LFP dynamics would exhibit oscillations within the electrophysiological alpha band (8–13 Hz), characterized by waxing and waning patterns that underlie amplitude and phase network synchronizations, while maintaining quasi-stationary slow (less than 0.5 Hz) fluctuations in Glu_e_ and GABA_e_ through homeostatic balancing of neurotransmitter uptake and release rates (the section “*Constraining dynamical regimes*” in “*Methods*” offers more details). These criteria led us to define 1225 distinct pairs of (*ω*_Glu_;*ω*_GABA_) parameters. Each pair was subjected to ten simulations, with each simulation lasting 120 seconds across a network comprising 216 nodes (see also “*Simulation scheme*” in “*Methods*”). The analysis encompassed both whole-brain activity and connectivity, employing a bifurcation-based computational framework to interpret the findings (Fig 2B and 2C).

### Neuron-astrocyte network activity analysis

For the activity analysis, detailed in the “*Neuron-astrocyte network activity analysis*” section under “*Methods*”, we focused on extracting whole-brain metrics and identifying spatial patterns through regional temporal means and temporal standard deviations.

**Fig 3:**
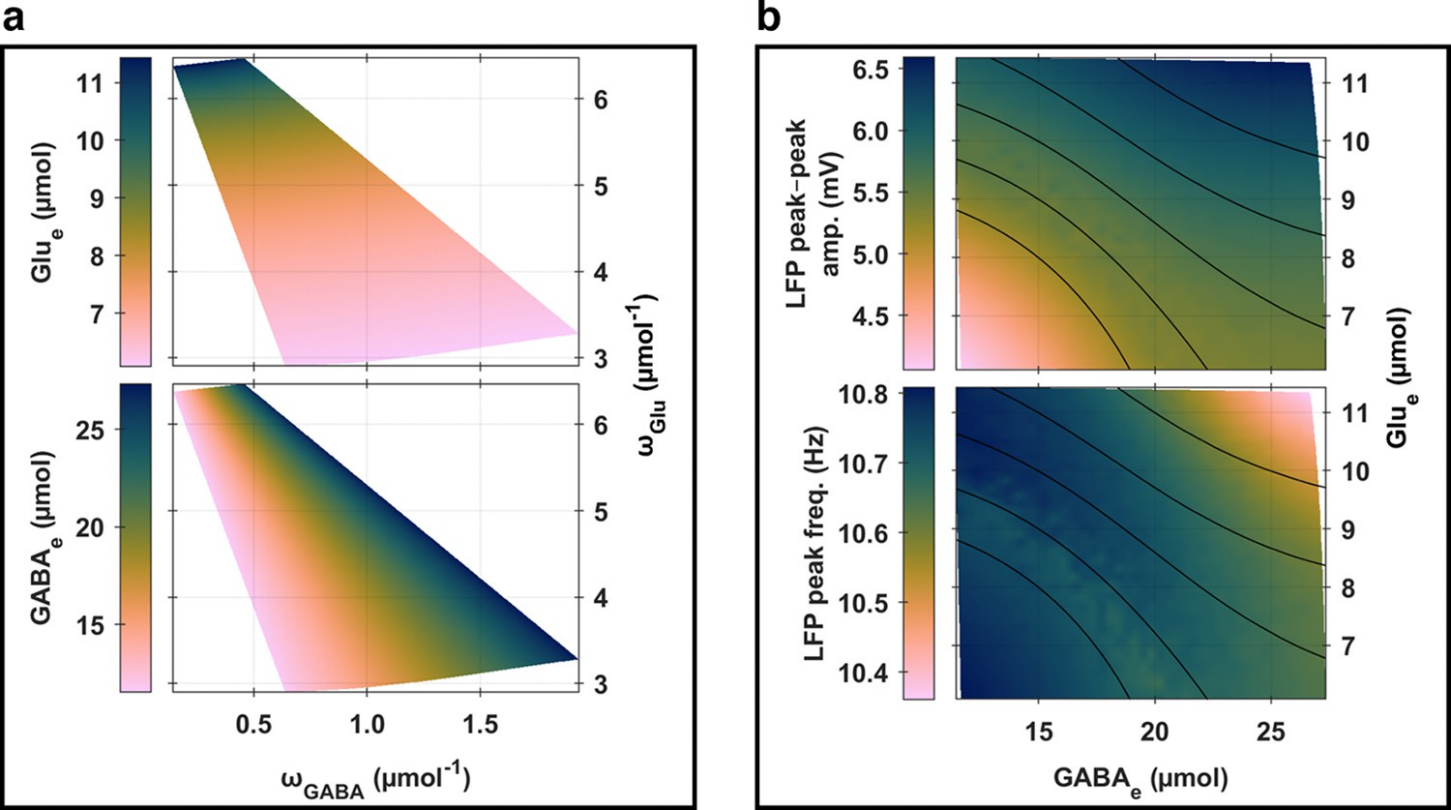
Global analysis of whole-brain activity. **(a) Links between simulation parameters and empirically concrete state variables.** The heatmaps show variations in whole-brain levels of Glu_e_ (top tile) and GABA_e_ (bottom tile) as functions of *ω*_Glu_ (vertical axis) and *ω*_GABA_ (horizontal axis). **(b) Links between electrophysiology and neurotransmission.** The heatmaps depict whole-brain levels of LFP peak-to-peak amplitudes (top tile) and peak frequencies (bottom tile) as functions of whole-brain concentration levels of Glu_e_ (vertical axis) and GABA_e_ (horizontal axis). The black solid curves represent isolines of periodic orbit peak-to-peak LFP amplitudes obtained through bifurcation analyses (refer to Fig 4), traversing specific (Glu_e_;GABA_e_) coordinates (in μmol×μmol): (8; 15), (9; 15), (10; 15), (11; 15), or (11; 20).

The relationship between the global coupling parameters (*ω*_Glu_;*ω*_GABA_) and whole-brain levels of empirically concrete state variables, Glu_e_ and GABA_e_, is illustrated in Fig 3A. On the one hand, we observed that increasing *ω*_Glu_, irrespective of *ω*_GABA_ levels, was linked to elevated levels of both Glu_e_ and GABA_e_. On the other hand, augmenting *ω*_GABA_, independent of *ω*_Glu_ adjustments, was associated with a slight reduction in Glu_e_ levels while still elevating GABA_e_ levels. These coherent interaction patterns between the simulation parameters (*ω*_Glu_ and *ω*_GABA_) and the two tangible neurophysiological variables (Glu_e_ and GABA_e_) were in accordance with the model’s parameterization and matched the theoretical anticipations discussed in the “*Network extension for the astrocytic compartment*” section within “*Model*” (see also section *S*2.*2* in *S2 File*). Notably, the dynamics of neurotransmitter releases and uptakes were designed to evolve over slow temporal scales (which are below 0.5 Hz, as shown in section *S2*.*4* in *S2 File*), with all parameter adjustments relative to a common baseline network state, leading to gradual and synchronous alterations in Glu_e_ and GABA_e_ levels. To further elucidate, an *ω*_Glu_ increase from the baseline state triggers a series of whole-brain events, amplifying glutamate release rates (*J*_Glu_) and subsequently raising Glu_e_ levels. This Glu_e_ increase, in turn, lowers excitability thresholds for pyramidal cells and inhibitory interneurons (*v*_Pyr_ and *v*_InIn_) which elevates their firing rates (*F*_Pyr_ and *F*_InIn_), and simultaneously boosts astrocytic network feedback on glutamate and GABA release rates ($Q_{Glu}^{Ast}$ and $Q_{GABA}^{Ast}$). The combination of enhanced neuronal firing rates and astrocytic feedback then leads to further increases in *J*_Glu_ and *J*_GABA_, and subsequently Glu_e_ and GABA_e_ levels. These processes continue until the uptake rates align upwards with the release rates. With *ω*_GABA_ elevation, a similar sequence of events occurs, aiming to balance the neurotransmitter release and uptake dynamics, leading to increased GABA_e_ and slightly decreased Glu_e_. It is crucial to note that the outcomes of the *ω*_Glu_ and *ω*_GABA_ pathways consistently represent the model’s equilibrium states, when the overall balance between neurotransmitter release and uptake rates remains fundamentally unaltered, despite stochastic disturbances or potential transient fluctuations (such as decreases in Glu_e_ due to increases in GABA_e_ along the pathway of *ω*_Glu_ elevations, or decreases in GABA_e_ due to decreases in Glu_e_ along the pathway of *ω*_GABA_ elevations). Furthermore, the funnel-shaped parameter space depicted in Fig 3A, outlined by (*ω*_Glu_;*ω*_GABA_), highlights the highly nonlinear influence of *ω*_Glu_ on neuron-astrocyte interactions in comparison to *ω*_GABA_. This differential impact is anticipated, given that extracellular glutamate levels not only affect the excitability thresholds of both pyramidal cells and inhibitory interneurons but also modulate astrocytic network feedback mechanisms.

A significant insight from the mappings in Fig 3A, between simulation parameters and state variables, was that each simulation, characterized by specific *ω*_Glu_ and *ω*_GABA_ values, could be uniquely identified by its Glu_e_ and GABA_e_ levels. This bijective correspondence enabled us to directly relate LFP characteristics to Glu_e_ and GABA_e_ levels, thereby providing a practical framework to discuss the interplay between electrical and chemical activities in the brain. As depicted in Fig 3B, variations in LFP peak-to-peak amplitudes and peak frequencies predominantly followed monotonic trends along the Glu_e_ and GABA_e_ axes. Specifically, both the peak-to-peak amplitude and the peak frequency of LFPs reached their extrema when Glu_e_ and GABA_e_ levels were at their collective maximums or minimums. Interestingly, intermediate levels of Glu_e_ and GABA_e_ were marked by local extrema in LFP characteristics. We gained further insights into these phenomena by formally characterizing the network’s oscillatory dynamics with bifurcation analysis.

**Fig 4:**
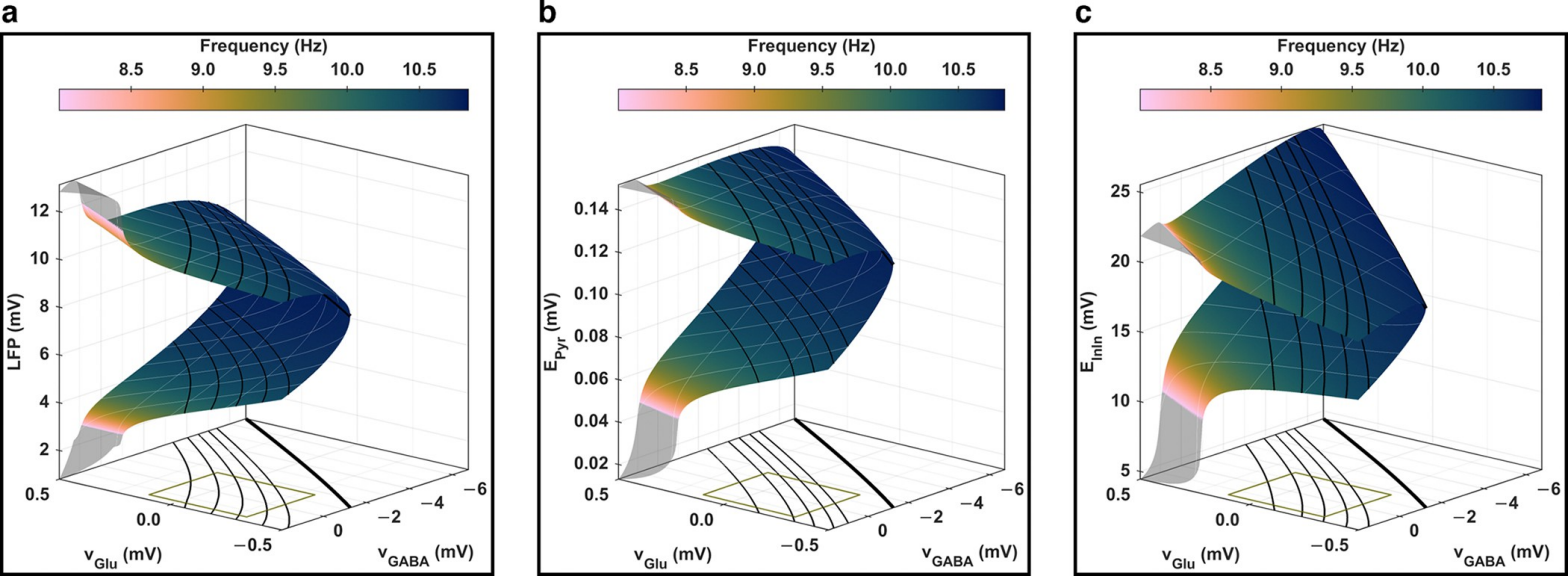
Two-parameter bifurcation landscapes of periodic orbits. **Bifurcation diagram drawn with (a) LFP**, **(b) *E***_**Pyr**_, and **(c) *E***_**InIn**_ as state variables, and *v*_Glu_ and *v*_GABA_ as bifurcation parameters. The depicted surface outlines the amplitude extrema of periodic orbits, with color gradients indicating frequencies. Areas left semi-transparent signify frequencies below eight hertz, while the black solid curves delineate contour lines of periodic orbit peak-to-peak amplitudes. The same isolines are graphed on a plane (*v*_Glu_;*v*_GABA_) under the surface, and the thickest black solid curves mark the loci of supercritical Poincaré–Andronov–Hopf bifurcation points, synonymous with a contour height of zero. As detailed in Eq *(5)*, *v*_Glu_ and *v*_GABA_ are sigmoidal functions of Glu_e_ and GABA_e_, respectively. The green rectangle beneath the surface demarcates the domain correspondence between (*v*_Glu_;*v*_GABA_) and (Glu_e_;GABA_e_) as drawn in Fig 3B, which further outlines the parameter space explored in the simulations (more details in section *S2*.*2* in *S2 File*). The isolines (except for the Hopf loci) pass through specific (Glu_e_;GABA_e_) coordinates (in μmol×μmol): (8; 15), (9; 15), (10; 15), (11; 15), and (11; 20). Notably, these isolines differ slightly across the LFP,*E*_Pyr_, and *E*_InIn_ landscapes, suggesting that each variable is sensitive to distinct phenomena within the network. This underscores the need to analyse multiple state variables to fully characterize the diverse neural dynamics within the network.

This analysis, focused on the amplitude and frequency properties of stable periodic orbits, as showcased in Fig 4, allowed us to identify a continuum where specific levels of glutamatergic and GABAergic activity enable the network model to transition between *physiological* and *epileptic-like* oscillatory behaviors. Specifically, under high Glu_e_ and GABA_e_ conditions, the network model verged on *spiking* behaviors, which are often used to simulate *low-frequency* (below the alpha band), *transient*, and *large peak-to-peak* oscillations characteristic of *epileptic-like* activity (further outlined in section *S2*.*1* in *S2 File*). This finding elucidates the trends observed in Fig 3B, where increasing peak-to-peak amplitudes and decreasing peak frequencies were noted. Additionally, the distribution of local extrema across intermediate Glu_e_ and GABA_e_ levels was captured by contour lines of periodic orbit peak-to-peak amplitudes (further illustrated in section *S5*.*1* in *S5 File*), indicating that the interplay between network activity characteristics and the underlying bifurcation landscapes of periodic orbits is intricately linked to the amplitude modulation of neuronal bioelectrical activity. Indeed, our network model was parameterized to persistently display oscillatory behaviors within the neuronal compartment, as evidenced by the bifurcation landscapes of stable periodic orbits depicted in Fig 4. Within these landscapes, the dynamics at each node are driven by Glu_e_ (mediated through *v*_Glu_),GABA_e_ (mediated through *v*_GABA_), neuronal network feedback inputs *Q*_Pyr_, and white noise *q*. The oscillatory drives, except for the white noise, are spatially structured through the astrocytic layer *Ω*_Ast_ and the neuronal layer *Ω*_Pyr_. This structuring confines each network node to specific areas of the bifurcation landscapes, where local variations in periodic orbit peak-to-peak amplitudes are pronounced, but changes in periodic orbit mean amplitudes or peak frequencies remain relatively subtle, as evidenced by Fig 3B (see also Figs B and D in *S2 File*). Furthermore, the model’s parameterization ensures that the neighborhoods covered on the parameter plane by each simulation do not overlap in terms of mean states. This non-overlapping ensures that each simulation explores a distinct zone of the periodic orbit bifurcation landscapes (refer to *Fig D* in *S2 File*), and allows the summarization of network behaviors in terms of whole-brain states as done in Fig 3. Consequently, the dynamics driven at each node by Glu_e_, GABA_e_, neuronal network feedback inputs *Q*_Pyr_, and white noise *q* give rise to spatially shaped amplitude-modulated oscillations within the neuronal compartment, which vary systematically across a continuum of glutamatergic and GABAergic activity. Moreover, while the periodic orbits in the neuronal compartment are responsible for the fastest oscillations in the model (10–11 Hz), the amplitude modulations of these orbits occur at much lower frequencies (less than 0.5 Hz), as illustrated in section *S2*.*4* in *S2 File*.

**Fig 5:**
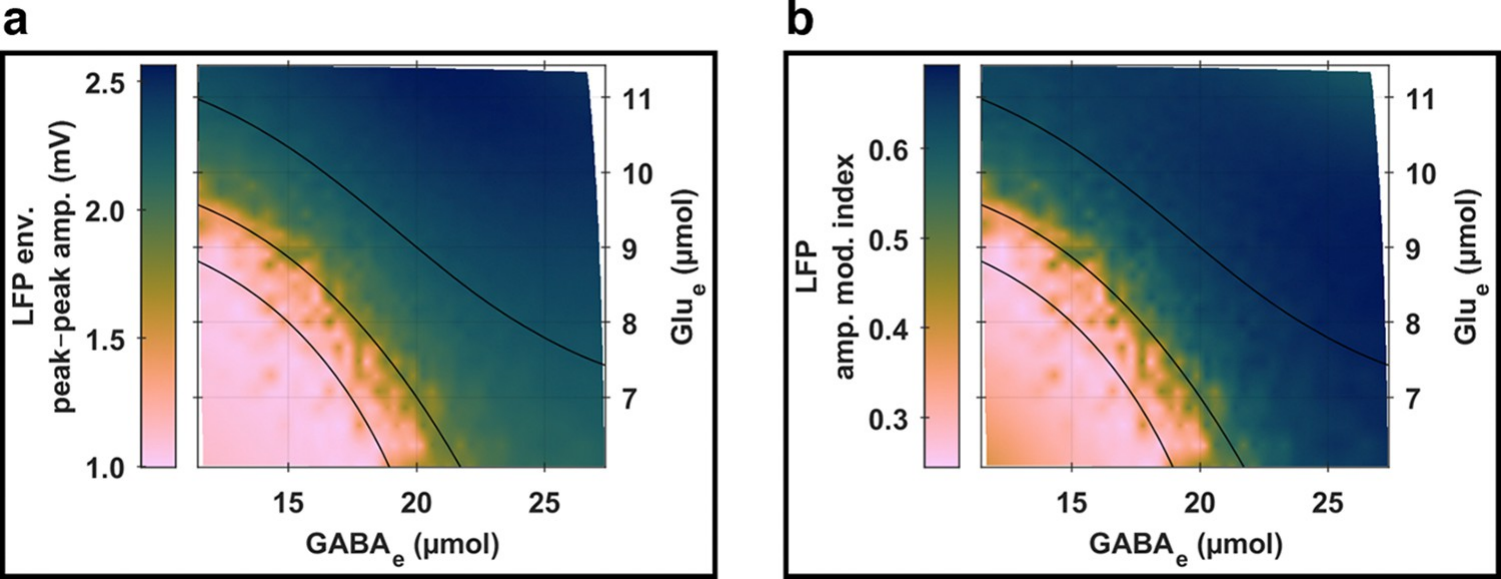
Global effects of neurotransmission on amplitude modulations of bioelectrical activity. **Whole-brain levels of (a) LFP envelope peak-to-peak amplitudes**, and **(b) LFP amplitude modulation indices**, as functions of whole-brain levels of Glu_e_ (vertical axis) and GABA_e_ (horizontal axis). The amplitude modulation index was calculated as the ratio of the LFP envelope peak-to-peak amplitude to the difference between the LFP signal peak-to-peak amplitude and the LFP envelope peak-to-peak amplitude. The black solid curves represent contour lines of periodic orbit LFP peak-to-peak amplitudes, consistent with those in Fig 6A. This alignment facilitates comparisons with the clustering analysis results of network activity patterns, which are presented in Fig 6A. Each isoline passes through specific (Glu_e_;GABA_e_) coordinates in (μmol)×(μmol): (8; 15), (7; 20), or [9,20].

For instance, Fig 5 presents the relationships between neurotransmitter concentration levels and contour lines of periodic orbit peak-to-peak amplitudes with LFP envelope peak-to-peak amplitude patterns (Fig 5A) and LFP amplitude modulation index patterns (Fig 5B). The variations observed closely align with the trends previously identified in Fig 3B (see section *S5*.*2* in *S5 File* for complementary results illustrating the correlations between the temporal fluctuations of LFP envelopes, Glu_e_, and GABA_e_). Importantly, Fig 5 highlights a set of critical isolines where profound changes in the amplitude modulation of LFP occur. These critical zones coincide with areas where more subtle changes in other network characteristics are consistently observed, as seen in Fig 3B. This finding reinforces the narrative developed earlier: that the distinctive amplitude properties of the periodic orbit bifurcation landscapes within the neuronal compartment, along with the resulting amplitude modulation of bioelectrical activity, are the principal factors driving global heterogeneity among network nodes (further discussed in section *S5*.*3* in *S5 File*). These amplitude properties promote the emergence of chimera states and metastable synchrony, driven by the interaction between independently sampled stochastic noise for each node and the structural layers of the network model.

**Fig 6:**
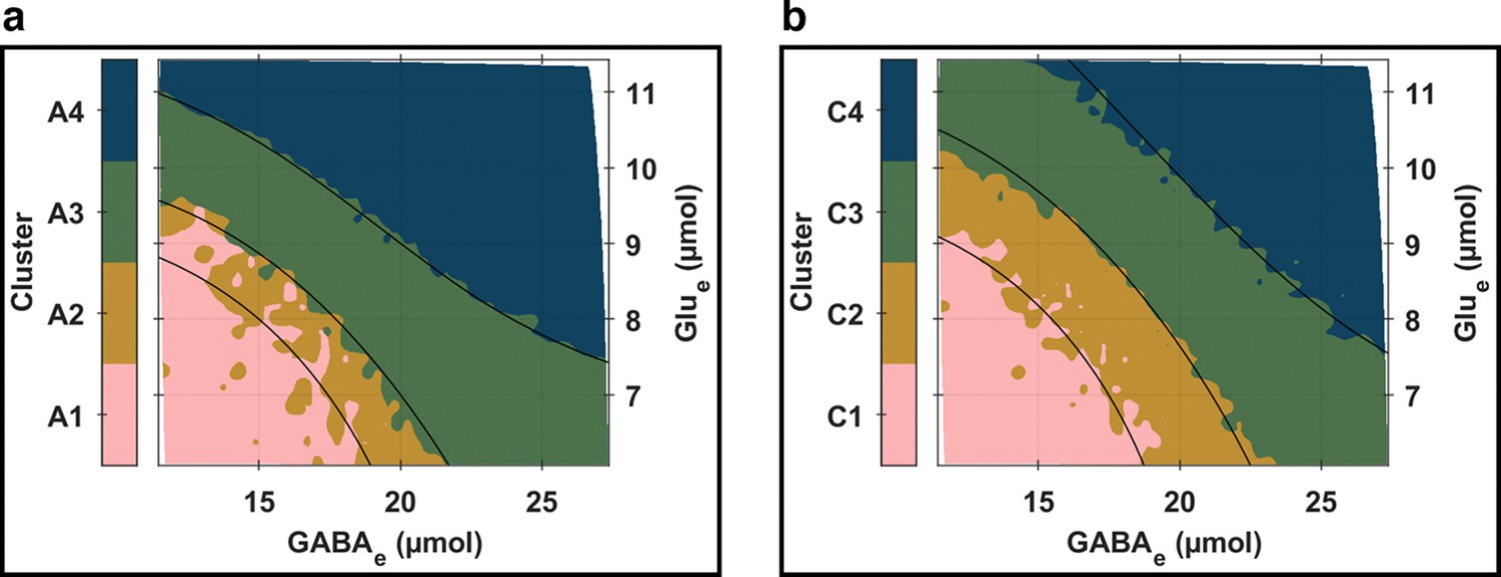
Clustering analysis of whole-brain network activity and connectivity patterns. **(a) Activity clustering.** This panel showcases the results of the clustering analysis based on the spatial patterns of normalized temporal standard deviations for LFP envelopes and normalized temporal means for Glu_e_ and GABA_e_. Each distinct color represents a cluster, corresponding to a unique partition of the (Glu_e_;GABA_e_) plane. These clusters can be directly mapped back to the simulation parameters (*ω*_Glu_;*ω*_GABA_) through a one-to-one relationship. The black solid curves illustrate the contour lines of periodic orbit peak-to-peak LFP amplitudes. These isolines pass through specific (Glu_e_;GABA_e_) coordinates (in μmol×μmol): (8; 15), (7; 20), and (9; 20). Further details on these analyses can be found in section *S5*.*4* in *S5 File*. **(b) Connectivity clustering.** This panel displays the clustering results related to the global topological properties of the reconstructed multilayer functional networks. Like panel **(a)**, each cluster is denoted by a specific color, indicating distinct partitions within the (Glu_e_;GABA_e_) plane. The black solid curves in this panel delineate the contour lines of periodic orbit peak-to-peak *E*_Pyr_ amplitudes, crossing through specific (Glu_e_;GABA_e_) coordinates (in μmol×μmol) at: (8.5; 14.0), (8.50; 18.25), and (9.5; 21.0). Additional insights are provided in section *S6*.*2* in *S6 File*.

Ultimately, the insights derived from Figs 3, 4, and 5 underscore the interplay between network activity characteristics and the underlying bifurcation landscapes of periodic orbits. We further explored these dependencies through a clustering analysis of the simulation parameter plane. This analysis utilized a Gaussian mixture model with the regional temporal standard deviations of LFP envelopes (serving as a proxy for regional LFP amplitude modulation index patterns), and the regional temporal means of Glu_e_ and GABA_e_ as predictors. The results of this clustering analysis are visually represented in Fig 6A, which identifies four distinct clusters within the simulation parameter plane. These clusters, which are mostly spatially contiguous, are primarily delineated by contour lines of periodic orbit peak-to-peak LFP amplitudes. Upon further inspection, we found that while periodic orbit *E*_Pyr_ and *E*_ExIn_ peak-to-peak amplitude isolines were less effective at delineating cluster boundaries, *E*_InIn_ demonstrated a level of effectiveness similar to LFP. These findings emphasize the dual roles of glutamatergic pyramidal neurons and GABAergic interneurons in shaping regional profiles of whole-brain network activity patterns: pyramidal neurons by integrating synaptic inputs, and interneurons by exerting critical inhibitory control over regional dynamics.

**Fig 7:**
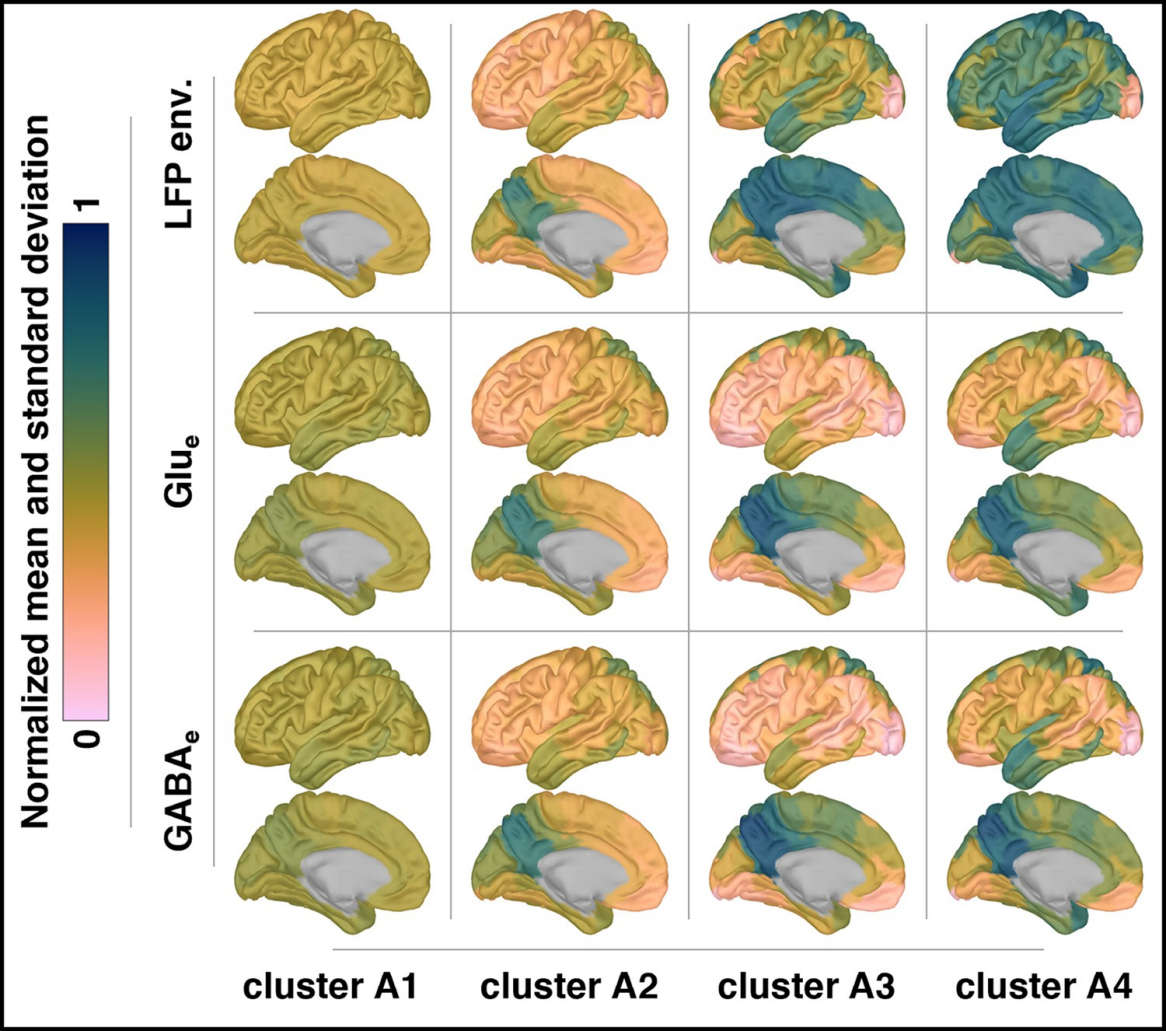
Cluster means from Gaussian mixture model analysis. This figure illustrates the cluster means for LFP envelopes, Glu_e_, and GABA_e_, as determined by a Gaussian mixture model with four components. The means represent the spatial patterns of normalized temporal standard deviations for LFP envelopes and normalized temporal means for Glu_e_ and GABA_e_. This figure complements the clustering analysis shown in Fig 6A. To aid in visualization and maintain simplicity, only patterns from the left hemisphere are shown, as they closely correspond to those in the right hemisphere (refer to *Fig F* in *S5 File* for full bilateral views).

To provide a clearer depiction of the defining regional characteristics of each identified cluster, Fig 7 presents the cluster means as fitted by the Gaussian mixture model. This visualization aids in understanding the specific effects that variations in the astrocytic network coupling parameters *ω*_Glu_ and *ω*_GABA_ have on the network’s behavior.

In cluster A1, regional patterns are relatively uniform across the brain. In contrast, clusters A2, A3, and A4 exhibit distinct spatial variations, with the precuneus and superior parietal cortices markedly differing from the lateral occipital, middle frontal, and temporal cortices. Additionally, while there is a noticeable shift in patterns between clusters A1 and A2, clusters A2, A3, and A4 demonstrate a more gradual progression of changes (see also section *S5*.*4* in *S5 File* for detailed illustrations). These spatial patterns in clusters A2, A3, and A4 particularly underscore the influence of the neuronal layer *Ω*_Pyr_. For example, in regions such as the precuneus, which are strongly connected within the neuronal layer *Ω*_Pyr_ (see Fig 10 and accompanying text in “*Methods*”), the highest mean levels of Glu_e_ and GABA_e_ were observed. These elevated levels resulted in more diverse periodic orbits, as indicated by a wider range of peak-to-peak amplitude distributions, ultimately leading to the most pronounced amplitude modulations. In contrast, the lateral occipital cortices, which are weakly connected within the neuronal layer and characterized by lower mean levels of Glu_e_ and GABA_e_, exhibited more homogeneous periodic orbit peak-to-peak amplitude distributions and the weakest amplitude modulations (refer also to section *S2*.*4* in *S2 File*). In general, the spatial patterns observed across all clusters reflect our simulation design and suggest that regions with elevated mean levels of Glu_e_ also exhibit higher mean levels of GABA_e_ and stronger LFP amplitude modulation, and conversely. This observation is consistent with the global relationships highlighted in Fig 5.

Moreover, as clusters progress from A2 to A4, regional differences become more subtle, accompanied by increasing modularity. This trend underscores the role of the astrocytic network in shaping dynamics across the simulation parameter plane (see also the properties of the astrocytic layer *Ω*_Ast_ in section S4.*3* in *S4 File*). For instance, in clusters A1 and A2, where network nodes display homogeneous behaviors or begin to exhibit mixed patterns akin to bifurcation phenomena, astrocytic network modulation is minimal due to low *ω*_Glu_ and *ω*_GABA_ values. In contrast, in clusters A3 and A4, where nodes exhibit more heterogeneous behaviors, the astrocytic network enforces consistent glutamatergic and GABAergic release rates across adjacent brain regions due to high *ω*_Glu_ and *ω*_GABA_ values. This homogenization of neurotransmitter dynamics tends to reduce the contrast between regional values.

Overall, these results from Fig 7 align with the narrative developed thus far, which outlines critical peak-to-peak amplitude values on the periodic orbit bifurcation landscapes where network characteristics undergo profound transformations due to heterogeneity induced by white noise across network nodes. Adjacent to these critical zones (cluster A2 in Fig 6A), the bifurcation landscapes divide into regions characterized by homogeneous network characteristics (cluster A1 in Fig 6A) and those showing a continuous spectrum of changing characteristics (clusters A3 and A4 in Fig 6A). These three distinct zones are reflected in the spatial patterns presented in Fig 7 (see also section *S5*.*4* in *S5 File*).

In sum, the integration of findings from this section elucidates the nuanced and diverse ways in which spatiotemporal whole-brain dynamics manifest across different types of activity (namely, LFP, Glu_e_, and GABA_e_) in response to astrocytic network modulation and white noise.

Moving forward, we apply multilayer network theory to capture the heterogeneous functional interactions among brain regions and dissect the simulated spatiotemporal whole-brain dynamics more thoroughly.

### Neuron-astrocyte network connectivity analysis

For the connectivity analysis, elaborated in the “*Neuron-astrocyte network connectivity analysis*” section under “*Methods*”, we reconstructed a four-layered multiplex functional network from the simulated whole-brain activities. This multiplex network, designed to cohesively capture various facets of neural communication, was structured using the identity matrix to represent inter-layer connections, and different bivariate statistical association measures to define intra-layer connections. Specifically, one intra-layer encoded the alpha-band-limited phase locking values (PLV) of LFP dynamics (LFP-PLV), a metric quantifying the similarity between instantaneous phases. Another intra-layer focused on the alpha-band-limited amplitude envelope Pearson-correlations (AEC) of LFP dynamics (LFP-AEC). The remaining layers were dedicated to capturing the Pearson-correlations of Glu_e_ or GABA_e_ dynamics (Glu_e_-C or GABA_e_-C, respectively).

**Fig 8:**
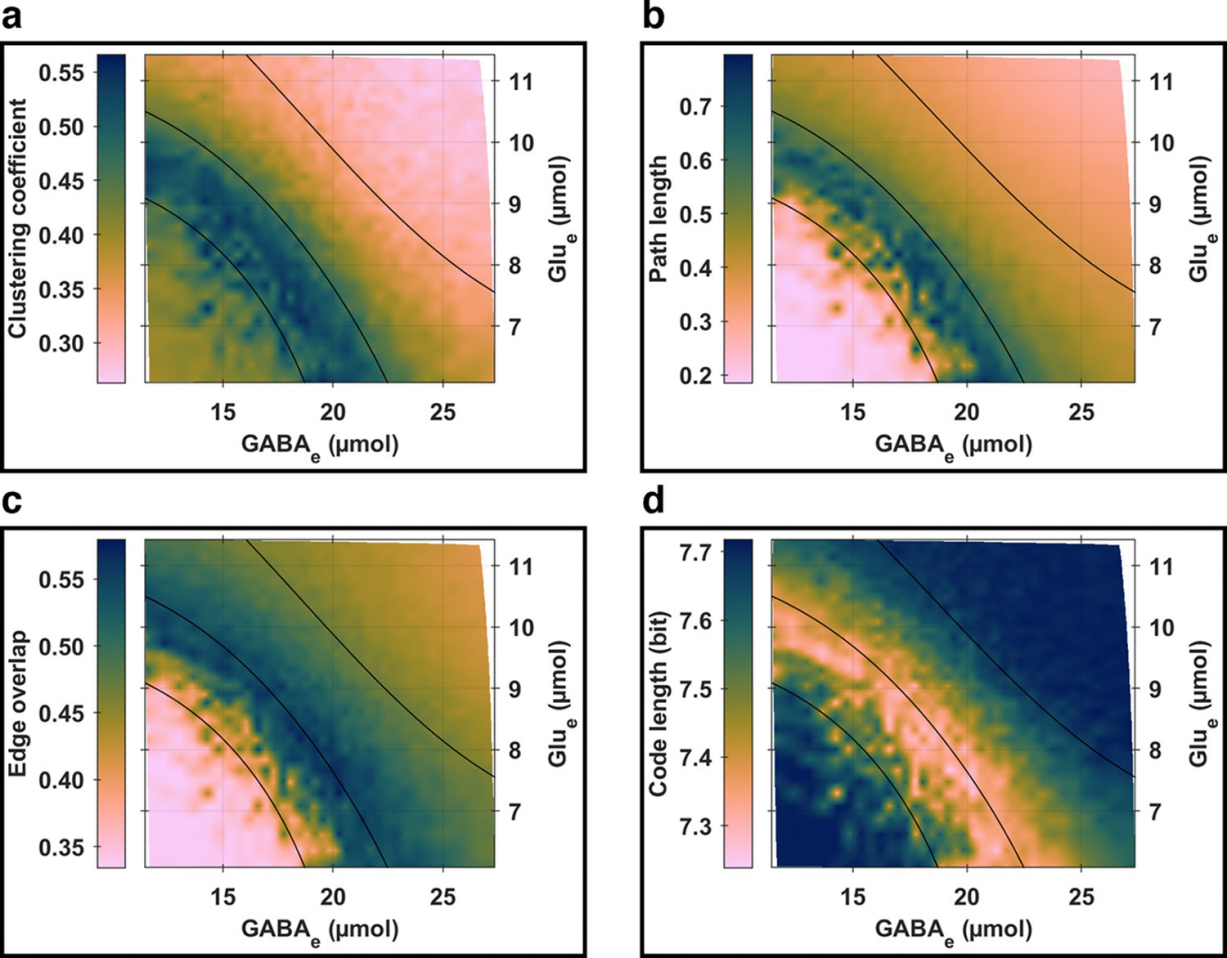
Global topological properties of multilayer functional networks. **(a) Clustering coefficient.** This metric serves as an indicator of network segregation, where higher values indicate more segregated networks. **(b) Path length.** This measure serves as an indicator of network integration, where higher values indicate more integrated networks. **(c) Edge overlap.** This quantity captures edge redundancy within the network, with higher values reflecting a greater consistency in connection weight patterns across different layers. **(d) Code length.** This is a quality index of community detection from information theory, with lower values reflecting networks with more optimal data compression of a random walker’s movements on them. **(a)–(d)** In each panel, the black solid curves represent the same contour lines of periodic orbit peak-to-peak *E*_Pyr_ amplitudes as in Fig 6B.

In Fig 8, we examine the relationships between four key global topological properties of the multilayer functional networks (clustering coefficient, path length, edge overlap, and code length) and the whole-brain levels of (Glu_e_;GABA_e_). The analysis reveals local extrema and generally monotonic trends that align with the contour lines of periodic orbit peak-to-peak *E*_Pyr_ amplitudes, suggesting a key role for glutamatergic pyramidal neurons in shaping whole-brain network synchrony, likely due to their predominant excitatory projections via white matter tracts (*Ω*_Pyr_). Complementary findings, including investigations of Von Neumann entropy, code length savings, and modularity, are provided in section *S6*.*1* in *S6 File*.

Additionally, a clustering analysis was conducted on the simulation parameter plane using a Gaussian mixture model. This model employed clustering coefficient, path length, edge overlap, and code length as predictors, elucidating the dependencies between network connectivity features and the bifurcation landscapes. Fig 6B showcases four clusters that closely mirror those identified in Fig 6A, which were originally used to inform the contour lines in Fig 8. Notably, cluster C2 predominantly encompasses the local extrema depicted in Fig 8 (further discussed in section *S6*.*2* in *S6 File*).

**Fig 9:**
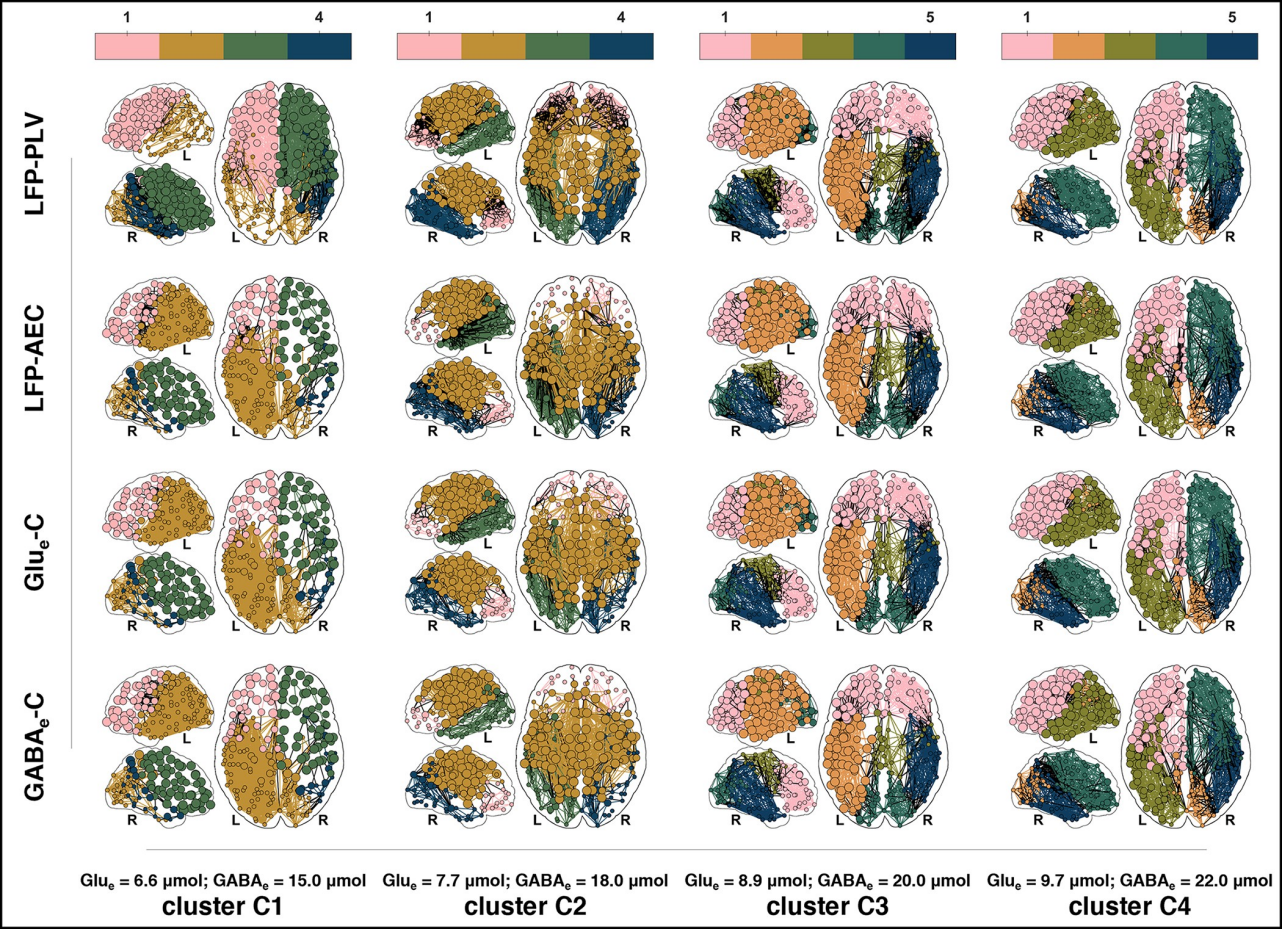
Mean multilayer functional networks. This figure shows the mean multilayer functional networks derived from four distinct simulations, each simulation representing a unique cluster identified in our analysis in Fig 6B. The layers within each network are displayed row-wise, while the column-wise arrangement corresponds to the simulations, differentiated by their whole-brain levels of (Glu_e_; GABA_e_). A color-coded legend above each column indicates the multilayer communities detected within that particular simulation, with node colors matching their assigned community. Within-community edges adopt the color of their respective community, while between-community edges are drawn in black. It is noteworthy that community alignment across layers is generally consistent, though not invariably so. Node sizes vary to represent their eigenvector versatility (an index of nodal centrality), categorized into three distinct ranges: small (from 0 to 1/3), medium (from 1/3 to 2/3), and large (from 2/3 to 1). To maintain visual clarity, only the top 10% of the most substantial edges by weight are shown in each layer, without specific coding for edge weights. The brain views provided include lateral left, lateral right, and dorsal perspectives, with “L” and “R” markers beneath each view indicating the posterior left and right sides, respectively. For detailed examination, including full adjacency matrices, refer to section *S6*.*3* in *S6 File*.

To visually represent the variability in functional network architectures, Fig 9 displays an exemplary multilayer network from each identified cluster. Key observations from this figure include the following. (i) Across all clusters, PLV-based layers demonstrated connectivity patterns that often contrasted with those observed in correlation-based layers. This contrast was manifested in preferences for short-range connections over long-range ones; or in the differentiation of specific regional connections, such as those between frontal–cingulate–parietal–insula regions as opposed to parietal–occipital–temporal connections, and *vice versa*. (ii) A consistent feature across all clusters was the similarity in connection densities between Glu_e_-C and GABA_e_-C layers, which were subtly different from those observed in LFP-AEC layers and clearly distinct from the patterns in LFP-PLV layers. (iii) The analysis further revealed notable variations in connection densities, centrality measures, and community organizations across all clusters.

**Fig 10:**
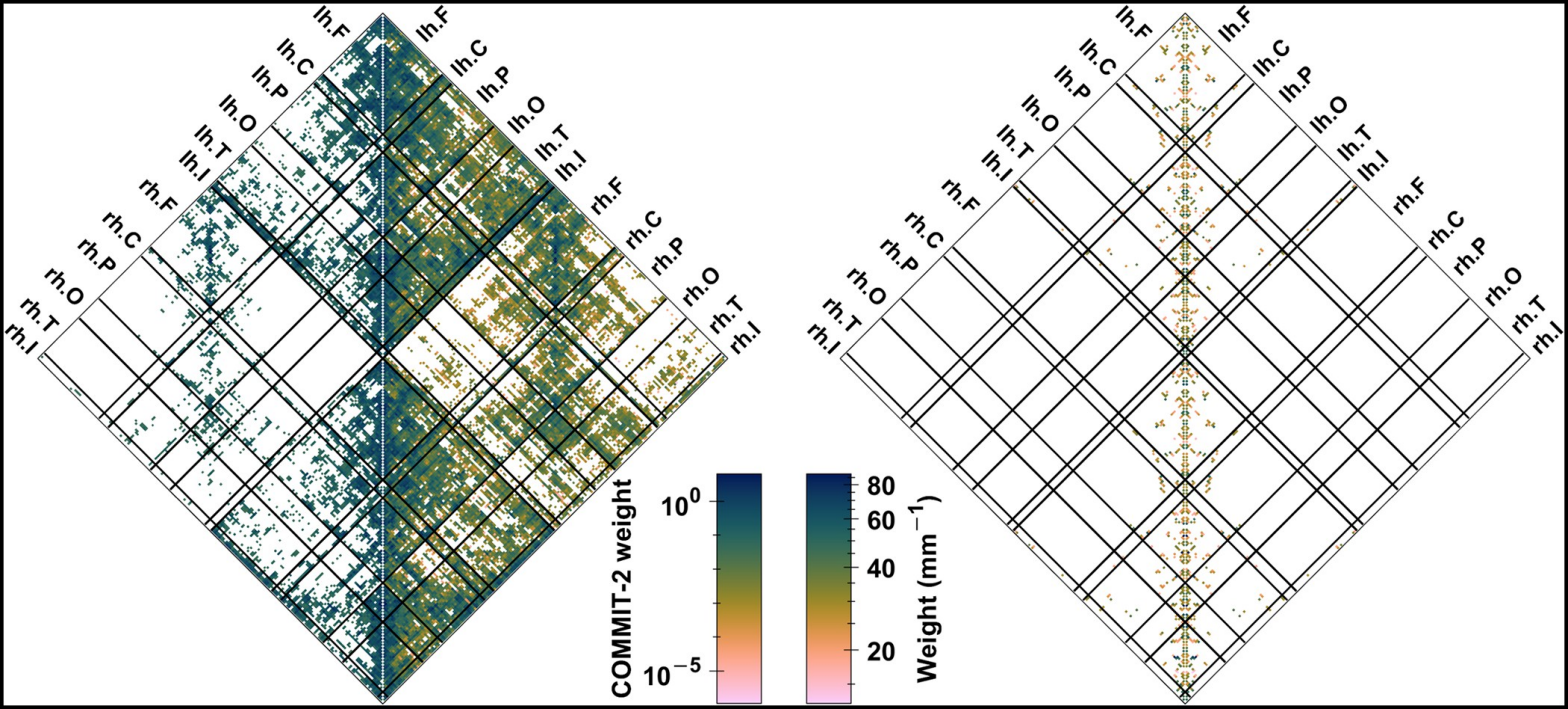
Neuronal and astrocytic structural layers. The neuronal layer (*Ω*_Pyr_) is shown on the left, and the astrocytic layer (*Ω*_Ast_) on the right. For *Ω*_Pyr_, a thresholded version of the connectome that retains the top 25 percent of connections by weight is displayed on the lower diagonal portion to facilitate the visualization of the strongest connections. This aids in the interpretation of functional connectomes. It is important to note that the neuronal interconnection connectome *Ω*_Pyr_ is an average derived from multiple subjects. Individual subject connectomes underwent biological filtering to achieve a target density of approximately 25 percent, whereas the averaged connectome was not filtered, resulting in a higher observed density of about 70 percent. This discrepancy underscores the potential implications of averaging connectomes, a topic that merits further investigation. The development of methodologies for deriving biophysically plausible connectomes, both at individual and group levels, is essential due to the significant role of these connectomes in constraining the main spatial and temporal interactions within dynamical models of whole-brain activity. The parcellation and regions follow the conventions specified in *S3 File*. Parcels in the left hemisphere are prefixed by “lh” and those in the right hemisphere by “rh”. F: frontal; C: cingulate; P: parietal; O: occipital; T: temporal; I: insula.

The interplay between functional network topologies, as captured by amplitude and phase coupling measures, provides a nuanced portrayal of the dynamics discussed in the earlier section, “*Neuron-astrocyte network activity analysis*”. In that section, we observed that the amplitude and frequency properties within the periodic orbit bifurcation landscapes of the neuronal compartments manifest as markedly distinct patterns across specific zones of the parameter plane. Within these zones, each property exhibits unique sensitivity to stochastic perturbations. Furthermore, in the whole-brain model, amplitude and frequency modulations become intrinsically coupled. This coupling is enhanced by spatial constraints and network topologies, which promote coherent behaviors within the network’s community organization (see Fig 10 and accompanying text in “*Methods*”). Indeed, synchrony in both amplitude and phase among nodes emerges simultaneously, depending on the variances in the spatially structured drives experienced by each neuronal compartment and the specific zones of their periodic orbit bifurcation landscapes that these drives sample. These synchrony patterns are primarily shaped by the neuronal layer *Ω*_Pyr_, whose small-world topology and community organization, together with stochastic perturbations from white noise, influence how each node’s dynamics navigate the state space defined by the bifurcation landscapes. Moreover, the astrocytic layer *Ω*_Ast_, with its lattice-like topology that reflects the brain’s geometrical embedding, also shapes these patterns by promoting intra-hemispheric and short-range couplings. Thus, amplitude and phase synchrony offer complementary insights into the network dynamics, at times diverging in topologies (clusters C1 and C2 in Fig 6B) or converging to similar patterns (clusters C3 and C4 in Fig 6B), influenced by the astrocytic network coupling parameters. An in-depth exploration of the relationship between phase-based and amplitude-based connectivity patterns is offered in section *S6*.*3* in *S6 File*.

In essence, the combined insights from Figs 6B, 8, and 9 underscore a broad spectrum of functional network topologies, shaped by different types of connectivity (LFP-PLV, LFP-AEC, Glu_e_-C, and GABA_e_-C). These topologies reflect the complex modulatory influences exerted by the astrocytic network on neuronal dynamics.

Lastly, to further elucidate the uniqueness of each connectivity layer, we undertook a *structural reducibility analysis* across all simulations. This investigation aimed to identify and merge layers that provided redundant topological information, thereby optimizing the architecture of each multilayer network. Interestingly, no merging was required in approximately half of the simulations (48.1%), highlighting the distinct topological contributions of each layer. In the remaining simulations, various merging configurations were observed: all correlation-based layers were merged in 31.7% of simulations, some correlation-based layers in 17.5%, and a combination of PLV-based with some correlation-based layers in 2.7%. Crucially, additional examinations of the reduced networks revealed that the insights derived from these optimized configurations closely mirrored those from the analysis of the original network sets. This consistency underscores the robustness of our findings, regardless of network simplification, as further elaborated in sections *S6*.*2* and *S6*.*4* in *S6 File*.

## Discussion

The past three decades have disclosed transformative revelations in neuroscience research, highlighting the profound impact of glial cells, including microglia, oligodendrocytes, and astrocytes, on brain functioning [1]. These findings emphasize the elaborate structural and functional interdependencies between glial cells and neurons, positioning glia not merely as adjuncts to neurons but as key figures in modulating brain architectures and dynamics [1]. The evolving narrative posits glia as critical regulators in both physiological and pathological contexts, at times even eclipsing neurons in their significance to brain health and disease mechanisms [18–20]. Despite the substantial advancements in understanding the roles of glia, a fundamental question persists: What exactly is the role of glia, alongside neurons, in health and disease states? This query remains at the forefront of neuroscientific inquiry, with numerous calls for the development of computational models capable of dissecting the complex interactions within neuron-glial networks [1,5].

Addressing this urgent need, our study introduced a dynamic model of whole-brain activity that simulates the reciprocal dialogue between neuronal and astrocytic networks through glutamatergic and GABAergic transmission pathways, with a particular focus on gliotransmission. Our examinations of astrocytic contributions to whole-brain activity and connectivity revealed that the astrocytic network, through gliotransmission, could orchestrate a diverse array of spatially structured neuronal dynamics, as evidenced by local field potential patterns, and shape profiles of excitatory and inhibitory activities, as indicated by extracellular levels of glutamate and GABA. Furthermore, our simulations unveiled a variety of emerging multilayer functional network topologies in response to astrocytic network modulation, derived from phase locking values and Pearson-correlations, which were influenced by the interplay between fast (10–11 Hz) and slow (less than 0.5 Hz) dynamic processes. In essence, our findings highlight the substantial contributions of astrocytic networks in enriching the interpretation of whole-brain activity and connectivity patterns, thereby promising to unveil new insights into the complex mechanisms underpinning brain function and dysfunction. The following paragraphs complement this overall summary.

Our investigation into whole-brain activity unveiled a notable relationship between glutamate, GABA, and local field potential patterns. The analysis suggests that the balance between excitatory and inhibitory neurotransmitter activities, governed by the interplay of neuron-astrocyte uptake and release mechanisms, plays a key role in modulating postsynaptic potential dynamics, as evidenced by fluctuations in the amplitude envelope of local field potentials. This observation is particularly noteworthy in the context of longstanding views that posit the coordinated action of multiple transmitters and modulators, both synaptically and extrasynaptically, as crucial in shaping the functional characteristics of neural circuits and significantly influencing their outputs [7,8,21–28]. Our findings, therefore, not only contribute to the broader understanding of excitation-inhibition balance frameworks [29], but also introduce a novel computational lens through which to examine the biochemical basis of whole-brain dynamics at spatiotemporal scales pertinent to neuroimaging data. For instance, a critical area of interest in neuroimaging research is the relationship between electrophysiological activities and hemodynamic changes, a topic that has garnered considerable attention over the past decade [30–33]. Notably, the alignment of network patterns observed in the band-limited amplitude envelopes of magnetoencephalography rhythms—particularly within the alpha and beta (13–30 Hz) bands—with those detected in blood-oxygen-level-dependent functional MRI signals has been a groundbreaking discovery [34]. This concordance has forged a multimodal bridge between the realms of non-invasive electrophysiological connectomics and established functional MRI connectomics, highlighting the critical role of whole-brain magnetoencephalography analyses in deciphering functional connectivity patterns [34]. Despite these advances, the mechanistic bases underlying the observed concordances have remained largely elusive. By incorporating the neuron-glial partnership into our whole-brain network modeling, we provide a novel vantage point for understanding how the intricate interplay between glutamatergic and GABAergic systems may shape the observed coupling between electrophysiological and hemodynamic rhythms in neuroimaging studies [5,35–39]. Below, we further discuss how our neuron-astrocytic network model can be enhanced by integrating vascular components to simulate cerebral blood flow, neurovascular coupling, and hemodynamics. Furthermore, our computational framework is not constrained to merely glutamatergic and GABAergic pathways. It opens the door to a more nuanced exploration of the brain’s chemical landscape, as we could envision incorporating other significant transmission and modulatory pathways such as cholinergic, dopaminergic, and serotonergic systems, with the hypothesis that astrocytic networks amplify and extend the reach of their effects on neuronal networks though calcium signaling and gliotransmitter release [40–42].

On the particular topic of electrophysiology–neurochemistry coupling, it is noteworthy that in recent years, the field of computational modeling has witnessed a significant paradigm shift, with many researchers expanding their focus from purely neuroelectrical dynamics to encompass the critical role of neurochemical activities and properties [10,23,25,28,43–47]. This broader perspective not only reflects advancements in empirical research [8,21–24,26–28,43,48–50], but also recognizes the complex interplay between electrical and chemical signaling in the brain, acknowledging that a truly comprehensive understanding of neural function necessitates considering both aspects at the very least. Despite this progress, many models continue to operate under the assumption that neuronal processes alone adequately represent the brain’s neurochemical features [10,23,25,28,43–47]. While this approach holds some merit in capturing certain physiological and pathological patterns, it also introduces ambiguities by overlooking the significant roles of non-neuronal cells [1,2,5,7,18–20,35,38,40,51–61]. Crucially, glial cells, particularly astrocytes, are central to regulating the brain’s neurochemical environment, offering multifaceted *support* for neurons that extends far beyond mere homeostatic balance and maintenance. Their contributions—spanning neurotransmitter recycling, metabolic and neurotrophic supports, orchestration of inflammation responses, maintenance and functional regulation of the blood-brain barrier, modulation of neurovascular coupling, and fine-tuning of synaptic and extrasynaptic activities—underscore the profound and complex interdependence of neuronal and glial processes [1,2,5,7,18–20,35,38,40,51–61]. The presumption that neuronal and glial activities are strongly coupled and thus can be modeled through a primarily neuronal lens is valid only under specific conditions but generally falls short of encompassing the brain’s diverse states and responses. For instance, during mental exertion, feeding, sleep, physical exercise, or amid neurological disorders, glial cells have been documented to exhibit intrinsic behaviors and regulatory mechanisms that may not directly correlate with or be predictable from neuronal activity alone [40,58,61–70]. In such contexts, glial cells can engage in autonomous functions, such as modulating the extracellular milieu, influencing blood flow, and regulating the brain’s immune responses, which may not be immediately apparent through the lens of neuronal dynamics, while being critical for the brain’s adaptability and resilience in response to varying physiological demands and pathological challenges. Therefore, these glial-mediated processes underscore the need for computational models that go beyond the neuron-centric paradigm, incorporating the dynamic contributions of both neuronal and glial compartments for a more accurate depiction of the brain’s neurochemical landscape. Embracing the complexity of neuron-glial interactions will undoubtedly enrich our understanding of brain functioning and pave the way for more nuanced and effective computational models that genuinely reflect the intricacies of neural systems.

In parallel with our exploration of whole-brain activity, our examination of whole-brain connectivity unveiled meaningful relationships between phase-based and amplitude-based network connectivity patterns. These patterns demonstrate sensitivity to distinct yet complementary spatiotemporal phenomena, highlighting the need for analytical frameworks capable of encapsulating the diversity of communication channels within neural networks. In this context, multilayer network modeling emerges as a potent tool, offering a comprehensive means to rigorously characterize dynamic network systems and deepen our grasp of the functional connectivity patterns that emerge within these networks, by accommodating multiple facets of connectivity [71–74]. Although our study primarily employed multilayer network modeling for illustrative purposes, the implications and applications of this approach are far-reaching (see also section *S6*.*3* in *S6 File*). In particular, the flexibility of our neuron-glial simulation framework facilitates the detailed examination of *coupled multilayer functional networks within the constraints of a multilayer structural network*, mirroring our assumption that whole-brain dynamics are governed by such a network architecture. Notably, our model’s astrocytic structural network was designed to reflect the brain’s geometrical embedding, aligning with recent research that posits geometrical constraints of the brain as potentially more influential on dynamics than axonal fiber connectivity, a perspective that challenges traditional understandings [10,75,76]. By integrating these innovative concepts through neuron-glial formalisms, our study advances a more refined perspective on whole-brain structure–function coupling.

The utility of bifurcation theory in elucidating stochastic network dynamics is also worth discussing. Indeed, integrating bifurcation analysis into our whole-brain model has proven pivotal in deciphering the dynamic properties of network activity and connectivity, with a particular emphasis on the roles played by heterogeneous stochastic fluctuations and contour lines of stable periodic orbit peak-to-peak amplitudes. These contour lines represent high-order bifurcation features rarely explored in traditional bifurcation analysis, and they provide a novel perspective on the nuanced organization of the network’s dynamic landscape. This methodological choice has substantially deepened our biophysical insights into the interactions across different neural compartments, the impact of structural network constraints, and the emergent patterns of functional connectivity (see also [77,78]). Interestingly, our application of clustering techniques alongside bifurcation analysis revealed a pronounced sensitivity of the stochastic network to deterministic stability and bifurcation events. This heightened sensitivity highlighted the network’s delicate balance, where minor adjustments in system parameters could lead to profound yet predictable shifts in stochastic network states. As extensively discussed in *S5 File* and *S6 File*, our findings demonstrate how bifurcation theory can be employed to probe dynamic network behaviors within a continuous landscape of stable periodic orbits, offering an indirect approach to examining oscillatory network dynamics while bypassing some of the complexities associated with the direct application of stochastic bifurcation theory. Future investigations could expand upon these findings by introducing additional complexities into the model, such as axonal transmission delays, or by exploring landscapes of periodic orbits with oscillatory frequencies beyond the initial range of 10–11 Hz.

More generally, our research underscores the necessity of incorporating glial cells into biophysical models of whole-brain activity, given their dynamic and foundational coalescence with neurons [1,5]. Moreover, utilizing computational whole-brain models that synergize with empirical findings offers a promising path to explore long-standing neuron-glial research questions, which have been challenging to investigate due to empirical limitations. For instance, this integrated whole-brain approach could be particularly valuable for examining the functional specialization of neuron-glial assemblies and understanding how glial signaling influences higher-order brain functions [1,5]. Indeed, this computational approach advocates for a mathematical representation of coupled neural activities through a wide spectrum of abstraction levels [9,10]. These levels range from phenomenological descriptions to those strictly grounded in fundamental biophysical principles, bypassing the need for detailed neurophysiological characterizations of individual cells. This modeling philosophy is predicated on the hypothesis that the core functionalities of the brain—such as movement, cognition, and perception—emerge from the collective behavior of neural cells within cortical circuits and across large-scale systems. Importantly, the outputs of these whole-brain models, along with key parameters, are designed to align with the spatiotemporal resolutions afforded by non-invasive neuroimaging techniques, including MRI, positron emission tomography, and electrophysiological recording methods [9,10]. This alignment enhances the models’ relevance and applicability to empirical data, facilitating the validation of theoretical predictions and the biophysical interpretation of complex neuroimaging findings. Additionally, while these models predominantly capture mesoscopic and macroscopic brain activities, they are informed by underlying microscale phenomena, enabling an integrated understanding across different scales of brain function [9,10].

Within this computational framework, the compartmental modeling strategy provides a versatile way for enhancing existing neuronal models, many of which are based on population firing rates [10,79–81]. By integrating an astrocytic compartment, these neuronal-centric models can be significantly enriched to encompass glial dynamics without negating the valuable insights previously garnered. As a proof of concept, our bifurcation analysis was specifically tailored to focus on neuronal dynamics, reinterpreting the bifurcation features of the Jansen–Rit model through the lens of glutamatergic and GABAergic neurotransmissions’ influences [11,82]. We employed a quasi-steady-state approximation to explore how slow changes in extracellular glutamate and GABA levels influence qualitative changes in alpha band neuronal dynamics, a process detailed in *S2 File*. This approach underscores the potential of compartmental modeling to capture the multifaceted aspects of whole-brain activity in a coherent and convenient manner [83,84]. Given the wide array of neuronal [9,10,79–81] and glial [1,2,14,18,58,60] modules documented in the literature, each characterized by unique and often complementary biophysical processes, the compartmental modeling scheme facilitates a flexible interchange of these components. This adaptability enables the customization of models to address specific phenomena or to achieve varying degrees of realism, according to theoretical and empirical needs. However, achieving an accurate bidirectional coupling between neuronal and glial compartments remains a complex endeavor, necessitating a judicious approach to model development, to preserve biological fidelity at the spatiotemporal scales of interest while maintaining computational feasibility. Additionally, a recurring question in modeling efforts, especially those aimed at interpreting neuroimaging data, concerns the optimal balance between model versatility and realism. Determining the necessary and sufficient data to constrain models and ensure their relevance to predict empirical observations remains an open challenge [9,10,74].

Indeed, the feasibility of validating dynamic whole-brain models using neuroimaging data critically depends on advancements in empirical data analysis, an area that is still developing [9,10,74]. The primary methodological challenges involve designing forward or inverse models that allow for a meaningful comparison between model outputs and empirical data [9,34,85–87]. From a neuron-glial perspective, the challenges are compounded by the lack of empirical methodologies capable of capturing both neuronal and glial activities simultaneously at the broader scales of population or whole-brain levels [5]. These limitations lead to a reliance on indirect datasets, such as those from MRI, positron emission tomography, or non-invasive electrophysiological measures [9]. These datasets can provide insights into neuron-glial interactions, although the transition to neuron-glial model outputs presents particular ambiguities, especially due to the traditionally neuron-centric nature of forward or inverse modeling [35–39,59,88,89]. Thus, precise mapping schemes to align neuron-glial model outputs with empirical data, while feasible, remain elusive until further research allows for the reinterpretation of neuroimaging datasets with an inclusive consideration of glial contributions [35–39,59,88,89]. A promising avenue for simulating hemodynamics within our whole-brain neuron-astrocyte modeling framework is suggested by the work in [12], which provides differential equations for simulating cerebral blood flow and neurovascular coupling within the mass modeling approach, while accounting for the modulation of vascular responses by astrocytes and neurons. Specifically, the work in [12] proposes that astrocytic populations, which play a predominant role in modulating blood flow, influence it indirectly based on their uptake dynamics of glutamate and GABA, through an impulse response that mimics slow-acting vasoactive mechanisms mediated by nitric oxide and epoxyeicosatrienoic acids, while neurons, with a more direct but relatively lesser impact, modulate blood flow based on their firing dynamics through a response function that mimics fast-acting vasoactive mechanisms mediated by dinoprostone, cyclooxygenase, and nitric oxide. The hemodynamic signal evoked by changes in cerebral blood flow could then be modeled using a balloon model [59,90]. Specific to astrocytes, calcium dynamics are posited as a key substrate for glial computations within the brain [5]. Our whole-brain model offers a pathway to integrate theoretical insights that establish a phenomenological link or incorporate a detailed biophysical description of astrocytic calcium dynamics [14]. This integration could offer partial means to constrain model outputs at mesoscopic scales, ensuring they more accurately reflect empirical observations, even considering the existing challenges in imaging astrocytic populations [5]. Last but not least, beyond the challenges of model outputs, the structural interconnections of astrocytic populations and the spatial heterogeneity of neuron-astrocyte distributions across the brain demand empirical elucidation as well (refer also to *S4 File* for a related discussion). For instance, astrocytic gap junction connectivity at the mesoscopic scales, or the effects of astrocyte-to-neuron ratios on population dynamics, are largely unexplored in existing literature [1,3,15,40]. To address this challenge, moleculo-cellular atlases like the *BigBrain Atlas* [91] and the *Allen Human Brain Atlas* [92], along with MRI, positron emission tomography, and electrophysiological data [50,93], hold promise for systematically unveiling these structural and functional aspects, contributing to a more comprehensive understanding of whole-brain network dynamics.

In summary, our framework introduces a broad platform for hypothesis generation and opens avenues to reassess the relationships between brain structure, function, electrophysiology, neurotransmission, neuromodulation, cerebral blood flow, and neurometabolism from a neuro-glio-vascular network perspective [1,6,12,19,40,53,57–59,88,89,94–96]. By incorporating vascular compartments [12], and a third structural layer representing vascular pathways [97], our model could offer integrative insights into the dynamic interplay between neural signaling, structural network topologies, vascular dynamics, and metabolic processes, applicable across health and disease states [51,98]. This approach aims to capture the essential, interdependent components of human brain functioning, reflecting the multifaceted network architectures within the brain. Furthermore, by developing a robust framework to bridge computational simulations with real-world neuroimaging and experimental data, for example through biophysics-constrained deep learning or probabilistic inference models [99], our whole-brain model could set the stage for unprecedented biologically faithful mesoscale and macroscale predictions. This approach is poised to profoundly advance our understanding of human brain dynamics across both health and disease states, significantly surpassing current modeling achievements.

## Methods

### Constraining dynamical regimes

This study utilized simulations to explore the contributions of astrocytic networks to whole-brain activity and the emergence of functional connectivity patterns. Central to this investigation was an analysis of how variations in the global astrocytic network coupling parameters, *ω*_Glu_ and *ω*_GABA_, influenced the dynamics of the network model. These parameters were pivotal in determining the impact of astrocytic network activity on glutamatergic and GABAergic neurotransmissions, which in turn affected neuronal firing rates.

To establish a biologically plausible exploration plane for the parameters (*ω*_Glu_;*ω*_GABA_), a set of criteria was defined ensuring that key model outputs such as LFP, Glu_e_, and GABA_e_ aligned with characteristics typically observed in empirical resting-state human data. The network parameterization was tailored to produce LFP dynamics with alpha band (8–13 Hz) oscillations [34], marked by waxing and waning patterns that facilitate amplitude and phase network synchronizations, while simultaneously preserving quasi-stationary slow (less than 0.5 Hz) fluctuations in Glu_e_ and GABA_e_ through the homeostatic regulation of neurotransmitter uptake and release rates.

The parameterization approach involved implementing a quasi-steady-state approximation to manage the slow temporal changes in Glu_e_ and GABA_e_ levels, which are markedly slower compared to the rapid dynamics of the neuronal compartment. This approximation simplified a numerical bifurcation analysis, focusing primarily on the neuronal compartment of the neuron-astrocyte mass model as detailed in section *S2*.*1* in *S2 File*. The goal was to illustrate that insights gained from this simplified bifurcation diagram could effectively predict the dynamical states of the neuron-astrocyte network model under conditions of weak-to-intermediate global neuronal network coupling and the influence of stochastic noise inputs (see also section *S2*.*2* in *S2 File*). This predictive capability was particularly relevant given that the structural matrices *Ω*_Pyr_ and *Ω*_Ast_ of the network model were probabilistically row-normalized. Furthermore, all nodes maintained stable periodic orbits within their neuronal compartments by sharing identical parameters, except each node received independently sampled stochastic noise inputs from the same normal distribution, via their parameter *q*. Importantly, to maintain a focus on neuron-astrocyte interactions and the structural layers of the model, rather than on the realism of whole-brain (neuronal) dynamics, delays due to axonal transmission were not implemented, and a homogeneous parameterization across network nodes was chosen.

The parameterization strategy was meticulously crafted, incorporating physiologically plausible parameter sets sourced from existing literature (detailed in *Table B* in *S1 File*) and a numerical bifurcation analysis of the neuron-astrocyte mass model to assess how variations in *v*_Glu_ and *v*_GABA_ could induce qualitative changes in neuronal dynamics (further explained in sections *S2*.*1* and *S2*.*2* in *S2 File*). This strategy enabled the following: (i) establish concentration intervals for Glu_e_ ([5; 15] μmol) and GABA_e_ ([5; 35] μmol) within which the neuronal compartment would show heightened sensitivity to modulatory impacts; (ii) constrain the neuronal compartment to display persistent oscillatory behaviors (stable periodic orbits) with frequencies within the [8; 13] Hz range and moderate peak-to-peak amplitudes; (iii) define an initial stable dynamical state proximate to a branch of supercritical Poincaré–Andronov–Hopf bifurcation points, ensuring the network model would exhibit baseline noise-modulated oscillatory activity; (iv) sample baseline neuronal firing rates *q*, independently for each node, from a normal distribution with a mean ± standard deviation of 240 ± 10 Hz to promote the emergence of chimera states and metastable synchrony, where nodes display transient and partially synchronized behaviors; (v) set *ω*_Pyr_ at 7.5 to facilitate amplitude and phase network synchronizations; and (vi) identify optimal pairs of values for *ω*_Glu_ ([2.90; 6.47] μmol^−1^) and *ω*_GABA_ ([0.14; 1.94] μmol^−1^), ensuring that variations in Glu_e_ and GABA_e_ would remain within the limits pre-specified in (i).

These methodological choices enabled the network model to generate a diverse array of spatially structured neuronal dynamical states. These states were characterized by a spectrum of stable periodic orbits with varying amplitude and frequency characteristics, alongside distinct profiles of spatially structured excitatory and inhibitory activities, as described in sections *S2*.*1*, *S2*.*2* and *S2*.*4* in *S2 File*. In particular, the interactions involving the heterogeneously specified stochastic noise inputs and the structural layers led to diverse clustered synchronized responses within the network. It is important to note that while the choices regarding the LFP frequency band, the concentration bounds for Glu_e_ and GABA_e_, and the initial state settings were easily generalizable, they came at the cost of reducing the physiological plausibility of certain neuronal compartment parameters, as discussed in section *S2*.*3* in *S2 File*.

### Defining structural layers

Empirical magnetic resonance imaging (MRI) data were utilized to construct the two structural connectivity matrices of the whole-brain network model: *Ω*_Pyr_ for neuronal populations and *Ω*_Ast_ for astrocytic populations. Both matrices were constructed within the anatomical constraints imposed by the *Lausanne-2018 surface-based atlas scale three* [100], which divides the cortex into 216 parcels (see also *S3 File*).

The matrix *Ω*_Pyr_ was derived from a state-of-the-art tractography-based connectome reconstruction pipeline applied to minimally preprocessed diffusion and structural MRI data from ten subjects in the *Human Connectome Project* (*Young Adult*) dataset [101,102]. This pipeline addressed streamline termination and quantification biases inherent in tractography [103]. It integrated three major components: (i) *Tractoflow* [104], a fully automated diffusion MRI processing pipeline; (ii) *SET*, or *Surface-Enhanced Tractography* [105], which uses cortical surface geometry priors to ensure that streamlines accurately intersect with the cortical surface, while also respecting the fanning patterns of fibers within the gyral blades; and (iii) *COMMIT-2*, or *a variant of convex optimization modeling for microstructure informed tractography* [106], which employs microstructural and anatomical priors to quantitatively filter and weight streamlines. Executed individually for each subject, this pipeline yielded quantitative connectomes, with connection weights reflecting an anatomical–microstructural measure of connectivity strength. The final matrix *Ω*_Pyr_ represented the average of these connectomes across subjects (detailed further in section *S4*.*1* in *S4 File*). As illustrated in Fig 10, the precuneus regions demonstrate the most substantial connectivity across this matrix, while the occipital regions display the fewest connections. Moreover, this matrix highlights strong interconnections within and between hemispheres in regions such as the frontal–cingulate–insula and parietal–occipital–temporal areas. Together, these connectivity patterns indicate distinct nodal importance and modular organization within the neuronal interconnection connectome *Ω*_Pyr_ (refer also to section *S4*.*3* in *S4 File*).

The matrix *Ω*_Ast_ was generated using a high-resolution tessellation of the mid-surface (the midpoint between the white and pial surfaces) of the *ICBM-2009c-asymmetric* template [107], processed with *FreeSurfer* [108]. Weights between adjacent parcels were calculated as the reciprocal of the geodesic distance between their mass centers, forming a lattice-like network that mirrors the brain’s geometrical embedding. This structure, representing immediate neighborhood connections along the cortical mantle, used physical proximity as a surrogate for astrocytic coupling facilitated by gap junctional densities (discussed further in sections *S4*.*2* and *S4*.*3* in *S4 File*).

Both *Ω*_Pyr_ and *Ω*_Ast_ matrices, illustrated in Fig 10, were normalized prior to initiating simulations: diagonal elements were set to zero, and each row was normalized so that the sum equals one. This normalization ensured that network inputs were proportionately distributed across each node, thereby facilitating balanced contributions and maintaining consistent input magnitudes within the network’s dynamics.

### Simulation scheme

To accurately model the complex, nonlinear interactions between glutamate and GABA dynamics, the simulation parameter plane defined by (*ω*_Glu_;*ω*_GABA_) was non-uniformly sampled. This strategy ensured that the whole-brain levels of Glu_e_ and GABA_e_ remained within the physiological ranges of [5; 15] μmol and [5; 35] μmol, respectively. A total of 1225 unique pairs of (*ω*_Glu_;*ω*_GABA_) were identified, which facilitated the uniform sampling of a grid defined by the whole-brain levels of (*v*_Glu_;*v*_GABA_). Detailed methodology is provided in section *S2*.*2* in *S2 File*.

The dynamic behavior of the whole-brain network model was governed by a system of coupled stochastic differential equations, consisting of 14 equations per node, resulting in a total of 3024 equations. These equations were numerically integrated using an in-house implementation of the stochastic Heun’s integration scheme, utilizing *MATLAB* version R2022a [109].

To ensure robustness and minimize biases introduced by transient dynamics, the simulation protocol was meticulously structured. Initially, a single calibration simulation lasting 370 seconds was conducted for each parameter pair (*ω*_Glu_;*ω*_GABA_) to establish a stable baseline. The stability and attainment of persistent oscillatory behaviors were visually confirmed in the final ten seconds of these calibration runs. These calibrated end states then served as the initial conditions for ten subsequent simulation batches, ensuring consistency and reliability across the dataset. For each parameter setting, ten 120-second simulations were conducted under distinct stochastic neuronal inputs (model parameter: *q*) and initial conditions, resulting in a total of 12250 simulations available for subsequent analyses. Further details are supplied in section *S2*.*2* in *S2 File*.

### Neuron-astrocyte network activity analysis

For each parameter pair (*ω*_Glu_;*ω*_GABA_), whole-brain values were derived from each of the ten simulation batches using the following approach: (i) instantaneous amplitude envelopes for the LFP dynamics were derived using a Hilbert transform, and whole-brain LFP peak-to-peak amplitude, or whole-brain LFP envelope peak-to-peak amplitude, was calculated as the mean of regional amplitudes; (ii) whole-brain LFP peak frequency was determined from the mean spectra of regional Welch’s power spectral density estimates; and (iii) whole-brain levels of Glu_e_ or GABA_e_, and the associated variables *v*_Glu_ or *v*_GABA_, were computed as the means of regional temporal means. These whole-brain quantities were chosen for analysis to validate the hypothesis that the network model, depending on the settings of *ω*_Glu_ and *ω*_GABA_, exhibits diverse neuronal dynamical states. These states are characterized by stable periodic orbits with varying peak-to-peak amplitudes and peak frequencies, along with distinct profiles of excitatory and inhibitory activities (refer to *S2 File* for more details).

To visualize the relationship between whole-brain quantities and the parameters (*ω*_Glu_;*ω*_GABA_) or the whole-brain levels of the variables (Glu_e_;GABA_e_), a two-dimensional natural neighbor interpolation method based on Delaunay triangulations was employed (*MATLAB*’s *scatteredInterpolant* function). This interpolation was performed for each simulation batch. Subsequently, the mean of the interpolated graphs across all simulation batches was calculated. Given that the whole-brain values of Glu_e_ and GABA_e_ showed minimal variation across batches, the mean of these interpolated graphs was found to be almost identical to individual graphs from each batch.

To perform hard clustering analysis, a Gaussian mixture model was applied to the dataset (*MATLAB*’s *fitgmdist* function). The variables analyzed included regional temporal standard deviations of LFP envelopes, and regional temporal means of Glu_e_ and GABA_e_ dynamics, with each neurophysiological data type comprising a total of 216 predictors. The dataset consisted of observations from all 12250 simulation runs. Each spatial profile of temporal standard deviations or temporal means was scaled to a range between zero and one, individually for LFP envelopes, Glu_e_ and GABA_e_, and separately for each simulation (see also section *S5*.*4* in *S5 File* for illustrations). The Gaussian mixture model was chosen for its ability to robustly capture the inherent spatial covariance among graph-based variables, present in the dataset across simulations. A full covariance structure common to all components was selected when fitting the mixture model to accurately model the interconnected nature of the data. This configuration effectively addressed the heterogeneity in cluster shapes and sizes. The model was independently fitted for potential groupings of four, five, or six components (see also section *S5*.*4* in *S5 File*). To ensure the stability and reliability of the clustering results, ten independent runs of the expectation-maximization algorithm were conducted for each component grouping. Each run was initialized using a *k*-means clustering heuristic. For each grouping scenario, the model configuration yielding the highest log-likelihood across the ten replicates was selected. Ultimately, the fitted model—either four, five, or six components—was chosen based on its ability to strike an optimal balance between minimizing the Akaike information criterion and maintaining model simplicity.

Visualization of the estimated clusters relative to the whole-brain levels of Glu_e_ and GABA_e_ was conducted by creating a heatmap. This heatmap was generated by first graphing the posterior probabilities of each component for each simulation batch using natural neighbor interpolations (*MATLAB*’s *scatteredInterpolant* function). The probabilities from these interpolations were then averaged across batches. Subsequently, cluster assignments for each observation were determined based on the highest posterior probability. It was confirmed that the mean interpolated heatmap accurately represented the individual batch graphs.

### Neuron-astrocyte network connectivity analysis

To effectively analyze functional connectivity, a multilayer network modeling approach was utilized, ideally suited for network systems with functional units that interact through diverse types of connection channels. For each parameter pair (*ω*_Glu_;*ω*_GABA_), a four-layered interconnected multiplex functional network was reconstructed from each of the ten simulation batches. In this multiplex network structure, the same brain region is represented across various layers, with each layer characterized by unique connectivity patterns that reflect distinct types of interactions. To maintain simplicity, inter-layer connectivity was uniformly defined by identity matrices, while each intra-layer was encoded with specific bivariate measures of functional connectivity [34,87]: (i) phase locking values of alpha-band-limited LFP dynamics (LFP-PLV), measuring consistency of phase differences, ranging from 0 for complete desynchronization to 1 for perfect phase synchronization; (ii) amplitude envelope Pearson-correlations of alpha-band-limited LFP dynamics (LFP-AEC); (iii) Pearson-correlations of Glu_e_ dynamics (Glu_e_-C); and (iv) Pearson-correlations of GABA_e_ dynamics (GABA_e_-C). This multilayer functional network was designed to elucidate the interplay between phase and amplitude couplings in LFP dynamics, driven by the spatially structured, slow fluctuations of Glu_e_ and GABA_e_ (further discussed in sections *S2*.*2* in *S2 File*, *S5* in *S5 File*, and *S6*.*3* in *S6 File*). Instantaneous phases and amplitude envelopes for the LFP dynamics were derived using a Hilbert transform. Subsequently, the amplitude envelopes and the dynamics of Glu_e_ and GABA_e_ were low-pass filtered using a 0.5 Hz cutoff frequency. Additionally, for simplicity, all Pearson-correlations were considered in absolute values. Exploratory analyses indicated that substituting Pearson-correlations with Spearman-correlations yielded similar findings and conclusions.

To enhance the clarity of visualizations, each pair of parameters (*ω*_Glu_;*ω*_GABA_) was analyzed across the ten simulation batches to generate a representative multilayer network, calculated as the mean across these batches. For the phase locking value layers, the arithmetic mean was used directly. In contrast, for the correlation layers, the arithmetic mean of Fisher’s z-transformed coefficients was back-transformed to compute the correlations [110]. Although visualizations were based on these averaged networks, the analyses systematically included both the original dataset, comprising 12250 individual networks, and the mean dataset, encompassing 1225 networks (see also section *S6*.*2* in *S6 File*). Prior to conducting multilayer network analyses, each functional layer within every multilayer network was thresholded to retain only the top 25 percent of the strongest connections.

The multilayer network analyses aimed to clarify the emerging functional connectivity patterns by quantifying various topological properties across *nodal*, *community*, and *global* scales. (i) At the nodal scale, the multilayer centrality of each node was evaluated using eigenvector versatility, a generalization of eigenvector centrality [111]. (ii) Multilayer community-level organizations were explored using the map equation method, which is grounded in information theory [112]. This method simulates the movement of a random walker and identifies multilayer communities by pinpointing groups of nodes among which the walker’s flow is optimally contained for extended periods. By minimizing the description length of the walker’s pathways, the map equation method effectively isolates groups of nodes that serve as information flow hubs within and across layers. (iii) At the global scale, the *global clustering coefficient* was calculated to measure network segregation, the *average path length* was assessed to determine network integration, the *global edge overlap* was analyzed to evaluate edge redundancy across the network layers, and the *code length*, derived from the map-equation-based community-level analysis, served as a quality index for assessing the effectiveness of community detection [113,114].

Structural reducibility analysis was implemented on each multilayer network to identify and merge similar layers. The quantum Jensen–Shannon divergence was used to quantify layer similarities, and arithmetic means were employed to aggregate similar layers. The topological properties of the resulting *reduced* networks were quantified in the same manner as those of the *original* networks. This approach effectively eliminated redundant or uninformative interactions within each reconstructed multilayer functional network [114], as further detailed in sections *S6*.*2* and *S6*.*4* in *S6 File*.

Hard clustering analyses utilized a Gaussian mixture model, following the approach outlined in the “*Neuron-astrocyte network activity analysis*” section, with modifications to the dataset and covariance structure (see also section *S6*.*2* in *S6 File*). The mixture model incorporated four standardized predictors—global clustering coefficient, average path length, global edge overlap, and code length—with a unique full covariance structure specified for each component.

Visualizations of both global topological properties and clusters, plotted relative to the whole-brain levels of Glu_e_ and GABA_e_, employed methodologies consistent with those applied in the “*Neuron-astrocyte network activity analysis*” section.

### Illustrations

To enhance data interpretation and ensure accessibility for readers with color-vision deficiencies, all visualizations were created using the *Scientific Colour Maps* package [115]. Brain maps and outlines were standardized using the *ICBM-2009c-asymmetric* template [107].

## Supporting information

S1 File Model variables and parameters. (PDF)

S2 File Constraining dynamical regimes. (PDF)

S3 File Parcellation. (PDF)

S4 File Structural layers. (PDF)

S5 File Neuron-astrocyte network activity analysis. (PDF)

S6 File Neuron-astrocyte network connectivity analysis. (PDF)

## References

[1] De Pittà M, Berry H. A Neuron–Glial Perspective for Computational Neuroscience. In: De Pittà M, Berry H, editors. Computational Glioscience [Internet]. Springer, Cham; 2019. p. 3–35. Available from: http://link.springer.com/10.1007/978-3-030-00817-8_1.

[2] De Pittà M. Neuron-Glial Interactions. In: Encyclopedia of Computational Neuroscience [Internet]. New York, NY: Springer New York; 2020. p. 1–30. Available from: http://link.springer.com/10.1007/978-1-4614-7320-6_100691-1.

[3] Vasile F, Dossi E, Rouach N. Human astrocytes: structure and functions in the healthy brain. Brain Struct Funct [Internet]. 2017 Jul 9;222(5):2017–29. Available from: http://link.springer.com/10.1007/s00429-017-1383-5. doi: 10.1007/s00429-017-1383-5 28280934 PMC5504258

[4] Goldberg M, De Pittà M, Volman V, Berry H, Ben-Jacob E. Nonlinear Gap Junctions Enable Long-Distance Propagation of Pulsating Calcium Waves in Astrocyte Networks. Gutkin BS, editor. PLoS Comput Biol [Internet]. 2010 Aug 26;6(8):e1000909. Available from: https://dx.plos.org/10.1371/journal.pcbi.1000909. 10.1371/journal.pcbi.1000909 PMC2928752 20865153

[5] Kastanenka K V., Moreno-Bote R, De Pittà M, Perea G, Eraso-Pichot A, Masgrau R, et al. A roadmap to integrate astrocytes into Systems Neuroscience. Glia [Internet]. 2020 Jan 6;68(1):5–26. Available from: https://onlinelibrary.wiley.com/doi/10.1002/glia.23632. 31058383 10.1002/glia.23632 PMC6832773

[6] Fields RD, Woo DH, Basser PJ. Glial Regulation of the Neuronal Connectome through Local and Long-Distant Communication. Neuron [Internet]. 2015 Apr;86(2):374–86. Available from: https://linkinghub.elsevier.com/retrieve/pii/S0896627315000409. doi: 10.1016/j.neuron.2015.01.014 25905811 PMC4426493

[7] Pacholko AG, Wotton CA, Bekar LK. Astrocytes—The Ultimate Effectors of Long-Range Neuromodulatory Networks? Front Cell Neurosci [Internet]. 2020 Sep 29;14(September):1–12. Available from: https://www.frontiersin.org/article/10.3389/fncel.2020.581075/full. doi: 10.3389/fncel.2020.581075 33192327 PMC7554522

[8] Marder E. Neuromodulation of Neuronal Circuits: Back to the Future. Neuron [Internet]. 2012 Oct;76(1):1–11. Available from: https://linkinghub.elsevier.com/retrieve/pii/S0896627312008173. doi: 10.1016/j.neuron.2012.09.010 23040802 PMC3482119

[9] Breakspear M. Dynamic models of large-scale brain activity. Nat Neurosci [Internet]. 2017 Mar 23;20(3):340–52. Available from: https://www.nature.com/articles/nn.4497. doi: 10.1038/nn.4497 28230845

[10] Griffiths JD, Bastiaens SP, Kaboodvand N. Whole-Brain Modelling: Past, Present, and Future. In: Advances in Experimental Medicine and Biology [Internet]. 2022. p. 313–55. Available from: https://link.springer.com/10.1007/978-3-030-89439-9_13. doi: 10.1007/978-3-030-89439-9_13 35471545

[11] Garnier A, Vidal A, Benali H. A Theoretical Study on the Role of Astrocytic Activity in Neuronal Hyperexcitability by a Novel Neuron-Glia Mass Model. The Journal of Mathematical Neuroscience [Internet]. 2016 Dec 21;6(1):10. Available from: http://mathematical-neuroscience.springeropen.com/articles/10.1186/s13408-016-0042-0. doi: 10.1186/s13408-016-0042-0 28004309 PMC5177605

[12] Blanchard S, Saillet S, Ivanov A, Benquet P, Bénar CG, Pélégrini-Issac M, et al. A New Computational Model for Neuro-Glio-Vascular Coupling: Astrocyte Activation Can Explain Cerebral Blood Flow Nonlinear Response to Interictal Events. Meuth SG, editor. PLoS One [Internet]. 2016 Feb 5;11(2):e0147292. Available from: doi: 10.1371/journal.pone.0147292 26849643 PMC4743967

[13] De Pittà M, Volman V, Berry H, Ben-Jacob E. A Tale of Two Stories: Astrocyte Regulation of Synaptic Depression and Facilitation. Gutkin BS, editor. PLoS Comput Biol [Internet]. 2011 Dec 1;7(12):e1002293. Available from: https://dx.plos.org/10.1371/journal.pcbi.1002293. 22162957 10.1371/journal.pcbi.1002293 PMC3228793

[14] Manninen T, Havela R, Linne ML. Computational Models of Astrocytes and Astrocyte–Neuron Interactions: Characterization, Reproducibility, and Future Perspectives. In: De Pittà M, Berry H, editors. Computational Glioscience [Internet]. Springer, Cham; 2019. p. 423–54. Available from: http://link.springer.com/10.1007/978-3-030-00817-8_16.

[15] Lallouette J, De Pittà M, Berry H. Astrocyte Networks and Intercellular Calcium Propagation. In: De Pittà M, Berry H, editors. Computational Glioscience [Internet]. Springer, Cham; 2019. p. 177–210. Available from: http://link.springer.com/10.1007/978-3-030-00817-8_7.

[16] Angulo MC, Le Meur K, Kozlov AS, Charpak S, Audinat E. GABA, a forgotten gliotransmitter. Prog Neurobiol [Internet]. 2008 Nov;86(3):297–303. Available from: https://linkinghub.elsevier.com/retrieve/pii/S0301008208000750. doi: 10.1016/j.pneurobio.2008.08.002 18786601

[17] Yoon BE, Lee CJ. GABA as a rising gliotransmitter. Front Neural Circuits [Internet]. 2014 Dec 17;8(DEC):1–8. Available from: http://journal.frontiersin.org/article/10.3389/fncir.2014.00141/abstract. doi: 10.3389/fncir.2014.00141 25565970 PMC4269106

[18] Volman V, Bazhenov M. Computational Models of Pathophysiological Glial Activation in CNS Disorders. In: De Pittà M, Berry H, editors. Computational Glioscience [Internet]. Springer, Cham; 2019. p. 289–305. Available from: http://link.springer.com/10.1007/978-3-030-00817-8_11.

[19] Kugler EC, Greenwood J, MacDonald RB. The “Neuro-Glial-Vascular” Unit: The Role of Glia in Neurovascular Unit Formation and Dysfunction. Front Cell Dev Biol [Internet]. 2021 Sep 27;9. Available from: https://www.frontiersin.org/articles/10.3389/fcell.2021.732820/full. 10.3389/fcell.2021.732820 PMC8502923 34646826

[20] Liddelow SA, Sofroniew M V. Astrocytes usurp neurons as a disease focus. Nat Neurosci [Internet]. 2019 Apr 11;22(4):512–3. Available from: https://www.nature.com/articles/s41593-019-0367-6. doi: 10.1038/s41593-019-0367-6 30858602

[21] Diao F, Elliott AD, Diao F, Shah S, White BH. Neuromodulatory connectivity defines the structure of a behavioral neural network. Elife [Internet]. 2017 Nov 22;6(1984):1–30. Available from: https://elifesciences.org/articles/29797. doi: 10.7554/eLife.29797 29165248 PMC5720592

[22] Del Guerra A, Ahmad S, Avram M, Belcari N, Berneking A, Biagi L, et al. TRIMAGE: A dedicated trimodality (PET/MR/EEG) imaging tool for schizophrenia. European Psychiatry [Internet]. 2018 Apr 1;50:7–20. Available from: https://www.cambridge.org/core/product/identifier/S0924933800007124/type/journal_article. doi: 10.1016/j.eurpsy.2017.11.007 29358016

[23] Shine JM. Neuromodulatory Influences on Integration and Segregation in the Brain. Trends Cogn Sci [Internet]. 2019 Jul;23(7):572–83. Available from: https://linkinghub.elsevier.com/retrieve/pii/S1364661319300944. doi: 10.1016/j.tics.2019.04.002 31076192

[24] Shine JM, Breakspear M, Bell PT, Ehgoetz Martens KA, Shine R, Koyejo O, et al. Human cognition involves the dynamic integration of neural activity and neuromodulatory systems. Nat Neurosci [Internet]. 2019 Feb 21;22(2):289–96. Available from: https://www.nature.com/articles/s41593-018-0312-0. doi: 10.1038/s41593-018-0312-0 30664771

[25] Kringelbach ML, Cruzat J, Cabral J, Knudsen GM, Carhart-Harris R, Whybrow PC, et al. Dynamic coupling of whole-brain neuronal and neurotransmitter systems. Proceedings of the National Academy of Sciences [Internet]. 2020 Apr 28;117(17):9566–76. Available from: https://pnas.org/doi/full/10.1073/pnas.1921475117. 32284420 10.1073/pnas.1921475117 PMC7196827

[26] Pierce S, Kadlaskar G, Edmondson DA, McNally Keehn R, Dydak U, Keehn B. Associations between sensory processing and electrophysiological and neurochemical measures in children with ASD: an EEG-MRS study. J Neurodev Disord [Internet]. 2021 Dec 6;13(1):5. Available from: https://jneurodevdisorders.biomedcentral.com/articles/10.1186/s11689-020-09351-0. doi: 10.1186/s11689-020-09351-0 33407072 PMC7788714

[27] Katz PS, Edwards DH. Metamodulation: the control and modulation of neuromodulation. In: Beyond Neurotransmission: Neuromodulation and its Importance for Information Processing [Internet]. Oxford University Press; 1999. p. 349–82. Available from: https://academic.oup.com/book/7917/chapter/153188048.

[28] Lea-Carnall CA, El-Deredy W, Stagg CJ, Williams SR, Trujillo-Barreto NJ. A mean-field model of glutamate and GABA synaptic dynamics for functional MRS. Neuroimage [Internet]. 2023 Feb;266(December 2022):119813. Available from: https://linkinghub.elsevier.com/retrieve/pii/S105381192200934X. doi: 10.1016/j.neuroimage.2022.119813 36528313 PMC7614487

[29] Sohal VS, Rubenstein JLR. Excitation-inhibition balance as a framework for investigating mechanisms in neuropsychiatric disorders. Mol Psychiatry [Internet]. 2019 Sep 14;24(9):1248–57. Available from: https://www.nature.com/articles/s41380-019-0426-0. doi: 10.1038/s41380-019-0426-0 31089192 PMC6742424

[30] Logothetis NK, Pauls J, Augath M, Trinath T, Oeltermann A. Neurophysiological investigation of the basis of the fMRI signal. Nature [Internet]. 2001 Jul 12;412(6843):150–7. Available from: https://www.nature.com/articles/35084005. doi: 10.1038/35084005 11449264

[31] Brookes MJ, Woolrich M, Luckhoo H, Price D, Hale JR, Stephenson MC, et al. Investigating the electrophysiological basis of resting state networks using magnetoencephalography. Proceedings of the National Academy of Sciences [Internet]. 2011 Oct 4;108(40):16783–8. Available from: https://pnas.org/doi/full/ doi: 10.1073/pnas.1112685108 21930901 PMC3189080

[32] Hipp JF, Hawellek DJ, Corbetta M, Siegel M, Engel AK. Large-scale cortical correlation structure of spontaneous oscillatory activity. Nat Neurosci [Internet]. 2012 Jun 6;15(6):884–90. Available from: https://www.nature.com/articles/nn.3101. doi: 10.1038/nn.3101 22561454 PMC3861400

[33] Ip IB, Berrington A, Hess AT, Parker AJ, Emir UE, Bridge H. Combined fMRI-MRS acquires simultaneous glutamate and BOLD-fMRI signals in the human brain. Neuroimage [Internet]. 2017 Jul;155(April):113–9. Available from: https://linkinghub.elsevier.com/retrieve/pii/S1053811917303233. doi: 10.1016/j.neuroimage.2017.04.030 28433623 PMC5519502

[34] Sadaghiani S, Brookes MJ, Baillet S. Connectomics of human electrophysiology. Neuroimage [Internet]. 2022 Feb;247(December 2021):118788. Available from: https://linkinghub.elsevier.com/retrieve/pii/S1053811921010600. doi: 10.1016/j.neuroimage.2021.118788 34906715 PMC8943906

[35] Figley CR, Stroman PW. The role(s) of astrocytes and astrocyte activity in neurometabolism, neurovascular coupling, and the production of functional neuroimaging signals. European Journal of Neuroscience [Internet]. 2011 Feb;33(4):577–88. Available from: https://onlinelibrary.wiley.com/doi/ doi: 10.1111/j.1460-9568.2010.07584.x 21314846

[36] Poskanzer KE, Yuste R. Astrocytes regulate cortical state switching in vivo. Proceedings of the National Academy of Sciences [Internet]. 2016 May 10;113(19):E2675–84. Available from: https://pnas.org/doi/full/ doi: 10.1073/pnas.1520759113 27122314 PMC4868485

[37] Wang M, He Y, Sejnowski TJ, Yu X. Brain-state dependent astrocytic Ca 2+ signals are coupled to both positive and negative BOLD-fMRI signals. Proceedings of the National Academy of Sciences [Internet]. 2018 Feb 13;115(7):E1647–56. Available from: https://pnas.org/doi/full/ doi: 10.1073/pnas.1711692115 29382752 PMC5816146

[38] Lu H, Jaime S, Yang Y. Origins of the Resting-State Functional MRI Signal: Potential Limitations of the “Neurocentric” Model. Front Neurosci [Internet]. 2019 Oct 23;13(October):1–8. Available from: https://www.frontiersin.org/article/10.3389/fnins.2019.01136/full. doi: 10.3389/fnins.2019.01136 31708731 PMC6819315

[39] Zimmer ER, Parent MJ, Souza DG, Leuzy A, Lecrux C, Kim HI, et al. [18F]FDG PET signal is driven by astroglial glutamate transport. Nat Neurosci [Internet]. 2017 Mar 30;20(3):393–5. Available from: https://www.nature.com/articles/nn.4492. doi: 10.1038/nn.4492 28135241 PMC5378483

[40] Verkhratsky A, Nedergaard M. Physiology of Astroglia. Physiol Rev [Internet]. 2018 Jan 1;98(1):239–389. Available from: https://www.physiology.org/doi/10.1152/physrev.00042.2016. 29351512 10.1152/physrev.00042.2016 PMC6050349

[41] González-Arias C, Perea G. Gliotransmission at Tripartite Synapses. In: De Pittà M, Berry H, editors. Computational Glioscience [Internet]. Springer, Cham; 2019. p. 213–26. Available from: http://link.springer.com/10.1007/978-3-030-00817-8_8.

[42] Lewis NE, Schramm G, Bordbar A, Schellenberger J, Andersen MP, Cheng JK, et al. Large-scale in silico modeling of metabolic interactions between cell types in the human brain. Nat Biotechnol [Internet]. 2010 Dec 21;28(12):1279–85. Available from: https://www.nature.com/articles/nbt.1711. doi: 10.1038/nbt.1711 21102456 PMC3140076

[43] Lawn T, Howard MA, Turkheimer F, Misic B, Deco G, Martins D, et al. From neurotransmitters to networks: Transcending organisational hierarchies with molecular-informed functional imaging. Neurosci Biobehav Rev [Internet]. 2023 Jul;150(April):105193. Available from: https://linkinghub.elsevier.com/retrieve/pii/S0149763423001628. doi: 10.1016/j.neubiorev.2023.105193 37086932 PMC10390343

[44] Jafarian A, Hughes LE, Adams NE, Lanskey JH, Naessens M, Rouse MA, et al. Neurochemistry-enriched dynamic causal models of magnetoencephalography, using magnetic resonance spectroscopy. Neuroimage [Internet]. 2023 Aug;276(May):120193. Available from: https://linkinghub.elsevier.com/retrieve/pii/S1053811923003440. doi: 10.1016/j.neuroimage.2023.120193 37244323

[45] Coronel-Oliveros C, Cofré R, Orio P. Cholinergic neuromodulation of inhibitory interneurons facilitates functional integration in whole-brain models. Marinazzo D, editor. PLoS Comput Biol [Internet]. 2021 Feb 18;17(2):e1008737. Available from: doi: 10.1371/journal.pcbi.1008737 33600402 PMC7924765

[46] Shine JM, Aburn MJ, Breakspear M, Poldrack RA. The modulation of neural gain facilitates a transition between functional segregation and integration in the brain. Elife [Internet]. 2018 Jan 29;7:1–16. Available from: https://elifesciences.org/articles/31130. doi: 10.7554/eLife.31130 29376825 PMC5818252

[47] Avery MC, Krichmar JL. Neuromodulatory Systems and Their Interactions: A Review of Models, Theories, and Experiments. Front Neural Circuits [Internet]. 2017 Dec 22;11(December):1–18. Available from: http://journal.frontiersin.org/article/10.3389/fncir.2017.00108/full. doi: 10.3389/fncir.2017.00108 29311844 PMC5744617

[48] Hansen JY, Shafiei G, Markello RD, Smart K, Cox SML, Nørgaard M, et al. Mapping neurotransmitter systems to the structural and functional organization of the human neocortex. Nat Neurosci [Internet]. 2022 Nov 27;25(11):1569–81. Available from: https://www.nature.com/articles/s41593-022-01186-3. doi: 10.1038/s41593-022-01186-3 36303070 PMC9630096

[49] Voigt K, Liang EX, Misic B, Ward PGD, Egan GF, Jamadar SD. Metabolic and functional connectivity provide unique and complementary insights into cognition-connectome relationships. Cerebral Cortex [Internet]. 2023 Feb 7;33(4):1476–88. Available from: https://academic.oup.com/cercor/article/33/4/1476/6570696. doi: 10.1093/cercor/bhac150 35441214 PMC9930619

[50] Shafiei G, Fulcher BD, Voytek B, Satterthwaite TD, Baillet S, Misic B. Neurophysiological signatures of cortical micro-architecture. Nat Commun [Internet]. 2023 Sep 26;14(1):6000. Available from: https://www.nature.com/articles/s41467-023-41689-6. doi: 10.1038/s41467-023-41689-6 37752115 PMC10522715

[51] Sweeney MD, Kisler K, Montagne A, Toga AW, Zlokovic B V. The role of brain vasculature in neurodegenerative disorders. Nat Neurosci [Internet]. 2018 Oct 24;21(10):1318–31. Available from: https://www.nature.com/articles/s41593-018-0234-x. doi: 10.1038/s41593-018-0234-x 30250261 PMC6198802

[52] St-Pierre MK, VanderZwaag J, Loewen S, Tremblay MÈ. All roads lead to heterogeneity: The complex involvement of astrocytes and microglia in the pathogenesis of Alzheimer’s disease. Front Cell Neurosci [Internet]. 2022 Aug 12;16(August). Available from: https://www.frontiersin.org/articles/10.3389/fncel.2022.932572/full. doi: 10.3389/fncel.2022.932572 36035256 PMC9413962

[53] Schaeffer S, Iadecola C. Revisiting the neurovascular unit. Nat Neurosci [Internet]. 2021 Sep 5;24(9):1198–209. Available from: https://www.nature.com/articles/s41593-021-00904-7. doi: 10.1038/s41593-021-00904-7 34354283 PMC9462551

[54] Liddelow SA, Barres BA. Reactive Astrocytes: Production, Function, and Therapeutic Potential. Immunity [Internet]. 2017 Jun;46(6):957–67. Available from: https://linkinghub.elsevier.com/retrieve/pii/S1074761317302340. doi: 10.1016/j.immuni.2017.06.006 28636962

[55] Lago-Baldaia I, Fernandes VM, Ackerman SD. More Than Mortar: Glia as Architects of Nervous System Development and Disease. Front Cell Dev Biol [Internet]. 2020 Dec 14;8(December):1–26. Available from: https://www.frontiersin.org/articles/10.3389/fcell.2020.611269/full. doi: 10.3389/fcell.2020.611269 33381506 PMC7767919

[56] Allen NJ, Lyons DA. Glia as architects of central nervous system formation and function. Science (1979) [Internet]. 2018 Oct 12;362(6411):181–5. Available from: https://www.science.org/doi/10.1126/science.aat0473. 10.1126/science.aat0473 PMC6292669 30309945

[57] Iadecola C. The Neurovascular Unit Coming of Age: A Journey through Neurovascular Coupling in Health and Disease. Neuron [Internet]. 2017 Sep;96(1):17–42. Available from: https://linkinghub.elsevier.com/retrieve/pii/S0896627317306529. doi: 10.1016/j.neuron.2017.07.030 28957666 PMC5657612

[58] Brazhe A, Verisokin A, Verveyko D, Postnov D. Astrocytes: new evidence, new models, new roles. Biophys Rev [Internet]. 2023 Oct 18;15(5):1303–33. Available from: https://link.springer.com/10.1007/s12551-023-01145-7. 10.1007/s12551-023-01145-7 PMC10643736 37975000

[59] Tesler F, Linne ML, Destexhe A. Modeling the relationship between neuronal activity and the BOLD signal: contributions from astrocyte calcium dynamics. Sci Rep [Internet]. 2023 Apr 20;13(1):6451. Available from: https://www.nature.com/articles/s41598-023-32618-0. doi: 10.1038/s41598-023-32618-0 37081004 PMC10119111

[60] Blum Moyse L, Berry H. Modelling the modulation of cortical Up-Down state switching by astrocytes. Migliore M, editor. PLoS Comput Biol [Internet]. 2022 Jul 21;18(7):e1010296. Available from: https://dx.plos.org/10.1371/journal.pcbi.1010296. doi: 10.1371/journal.pcbi.1010296 35862433 PMC9345492

[61] Sinha S, Patro N, Patro I. The Glial Perspective of Energy Homeostasis, Neuroinflammation, and Neuro-nutraceuticals. In: Patro I, Seth P, Patro N, Tandon PN, editors. The Biology of Glial Cells: Recent Advances [Internet]. Singapore: Springer Singapore; 2022. p. 627–52. Available from: https://link.springer.com/10.1007/978-981-16-8313-8_23.

[62] Garofalo S, Picard K, Limatola C, Nadjar A, Pascual O, Tremblay M. Role of Glia in the Regulation of Sleep in Health and Disease. In: Comprehensive Physiology [Internet]. Wiley; 2020. p. 687–712. Available from: https://onlinelibrary.wiley.com/doi/10.1002/cphy.c190022. 10.1002/cphy.c190022 32163207

[63] Oliveira JF, Araque A. Astrocyte regulation of neural circuit activity and network states. Glia [Internet]. 2022 Aug 22;70(8):1455–66. Available from: https://onlinelibrary.wiley.com/doi/10.1002/glia.24178. 35460131 10.1002/glia.24178 PMC9232995

[64] Salas IH, Burgado J, Allen NJ. Glia: victims or villains of the aging brain? Neurobiol Dis [Internet]. 2020 Sep;143(July):105008. Available from: https://linkinghub.elsevier.com/retrieve/pii/S0969996120302837. doi: 10.1016/j.nbd.2020.105008 32622920

[65] Augusto-Oliveira M, Arrifano GP, Takeda PY, Lopes-Araújo A, Santos-Sacramento L, Anthony DC, et al. Astroglia-specific contributions to the regulation of synapses, cognition and behaviour. Neurosci Biobehav Rev [Internet]. 2020 Nov;118(May):331–57. Available from: https://linkinghub.elsevier.com/retrieve/pii/S014976342030508X. 32768488 10.1016/j.neubiorev.2020.07.039

[66] Fields RD, Araque A, Johansen-Berg H, Lim SS, Lynch G, Nave KA, et al. Glial Biology in Learning and Cognition. The Neuroscientist [Internet]. 2014 Oct 11;20(5):426–31. Available from: http://journals.sagepub.com/doi/10.1177/1073858413504465. 24122821 10.1177/1073858413504465 PMC4161624

[67] Frank MG. The Role of Glia in Sleep Regulation and Function. In: Handbook of Experimental Pharmacology [Internet]. 2018. p. 83–96. Available from: http://link.springer.com/10.1007/164_2017_87. 10.1007/164_2017_87 29374835

[68] García-Cáceres C, Fuente-Martín E, Argente J, Chowen JA. Emerging role of glial cells in the control of body weight. Mol Metab [Internet]. 2012 Dec;1(1–2):37–46. Available from: https://linkinghub.elsevier.com/retrieve/pii/S221287781200004X. doi: 10.1016/j.molmet.2012.07.001 24024117 PMC3757650

[69] Kofuji P, Araque A. Astrocytes and Behavior. Annu Rev Neurosci [Internet]. 2021 Jul 8;44(1):49–67. Available from: https://www.annualreviews.org/doi/10.1146/annurev-neuro-101920-112225. 33406370 10.1146/annurev-neuro-101920-112225 PMC8257756

[70] Bernaus A, Blanco S, Sevilla A. Glia Crosstalk in Neuroinflammatory Diseases. Front Cell Neurosci [Internet]. 2020 Jul 29;14(July):1–17. Available from: https://www.frontiersin.org/article/10.3389/fncel.2020.00209/full. doi: 10.3389/fncel.2020.00209 32848613 PMC7403442

[71] De Domenico M. Multilayer modeling and analysis of human brain networks. Gigascience [Internet]. 2017 May 1;6(5):1–8. Available from: https://academic.oup.com/gigascience/article/doi/10.1093/gigascience/gix004/2968355. 28327916 10.1093/gigascience/gix004 PMC5437946

[72] Tewarie P, Hillebrand A, van Dijk BW, Stam CJ, O’Neill GC, Van Mieghem P, et al. Integrating cross-frequency and within band functional networks in resting-state MEG: A multi-layer network approach. Neuroimage [Internet]. 2016 Nov;142:324–36. Available from: https://linkinghub.elsevier.com/retrieve/pii/S1053811916303718. 10.1016/j.neuroimage.2016.07.057 27498371

[73] Tewarie P, Liuzzi L, O’Neill GC, Quinn AJ, Griffa A, Woolrich MW, et al. Tracking dynamic brain networks using high temporal resolution MEG measures of functional connectivity. Neuroimage [Internet]. 2019 Oct;200:38–50. Available from: https://linkinghub.elsevier.com/retrieve/pii/S1053811919304914. doi: 10.1016/j.neuroimage.2019.06.006 31207339

[74] Hallett M, de Haan W, Deco G, Dengler R, Di Iorio R, Gallea C, et al. Human brain connectivity: Clinical applications for clinical neurophysiology. Clinical Neurophysiology [Internet]. 2020 Jul;131(7):1621–51. Available from: https://linkinghub.elsevier.com/retrieve/pii/S1388245720301371. doi: 10.1016/j.clinph.2020.03.031 32417703

[75] Pang JC, Aquino KM, Oldehinkel M, Robinson PA, Fulcher BD, Breakspear M, et al. Geometric constraints on human brain function. Nature [Internet]. 2023 Jun 15;618(7965):566–74. Available from: https://www.nature.com/articles/s41586-023-06098-1. doi: 10.1038/s41586-023-06098-1 37258669 PMC10266981

[76] Roberts JA, Perry A, Lord AR, Roberts G, Mitchell PB, Smith RE, et al. The contribution of geometry to the human connectome. Neuroimage [Internet]. 2016 Jan;124:379–93. Available from: https://linkinghub.elsevier.com/retrieve/pii/S105381191500806X. 10.1016/j.neuroimage.2015.09.009 26364864

[77] Clusella P, Deco G, Kringelbach ML, Ruffini G, Garcia-Ojalvo J. Complex spatiotemporal oscillations emerge from transverse instabilities in large-scale brain networks. Gutkin BS, editor. PLoS Comput Biol [Internet]. 2023 Apr 12;19(4):e1010781. Available from: https://dx.plos.org/10.1371/journal.pcbi.1010781. doi: 10.1371/journal.pcbi.1010781 37043504 PMC10124884

[78] Forrester M, Crofts JJ, Sotiropoulos SN, Coombes S, O’Dea RD. The role of node dynamics in shaping emergent functional connectivity patterns in the brain. Network Neuroscience [Internet]. 2020 Jan;4(2):467–83. Available from: https://direct.mit.edu/netn/article/4/2/467-483/95823. doi: 10.1162/netn_a_00130 32537537 PMC7286301

[79] Liley DTJ. Neural Population Model. In: Jaeger D, Jung R, editors. Encyclopedia of Computational Neuroscience [Internet]. New York, NY: Springer New York; 2015. p. 1898–912. Available from: http://link.springer.com/10.1007/978-1-4614-6675-8_69.

[80] Chehelcheraghi M, van Leeuwen C, Steur E, Nakatani C. A neural mass model of cross frequency coupling. Cymbalyuk G, editor. PLoS One [Internet]. 2017 Apr 5;12(4):e0173776. Available from: https://dx.plos.org/10.1371/journal.pone.0173776. doi: 10.1371/journal.pone.0173776 28380064 PMC5381784

[81] Coombes S, Byrne Á. Next Generation Neural Mass Models. In: Corinto F, Torcini A, editors. Nonlinear Dynamics in Computational Neuroscience [Internet]. Springer International Publishing; 2019. p. 1–16. Available from: http://link.springer.com/10.1007/978-3-319-71048-8_1.

[82] Jansen BH, Rit VG. Electroencephalogram and visual evoked potential generation in a mathematical model of coupled cortical columns. Biol Cybern [Internet]. 1995 Sep;73(4):357–66. Available from: http://link.springer.com/10.1007/BF00199471. doi: 10.1007/BF00199471 7578475

[83] Sanz-Leon P, Knock SA, Spiegler A, Jirsa VK. Mathematical framework for large-scale brain network modeling in The Virtual Brain. Neuroimage [Internet]. 2015 May;111:385–430. Available from: https://linkinghub.elsevier.com/retrieve/pii/S1053811915000051. doi: 10.1016/j.neuroimage.2015.01.002 25592995

[84] Cook BJ, Peterson ADH, Woldman W, Terry JR. Neural Field Models: A mathematical overview and unifying framework. Mathematical Neuroscience and Applications [Internet]. 2022 Mar 19;Volume 2(2):1–67. Available from: https://mna.episciences.org/7284.

[85] Sotero RC, Trujillo-Barreto NJ. Biophysical model for integrating neuronal activity, EEG, fMRI and metabolism. Neuroimage [Internet]. 2008 Jan;39(1):290–309. Available from: https://linkinghub.elsevier.com/retrieve/pii/S1053811907007045. doi: 10.1016/j.neuroimage.2007.08.001 17919931

[86] Valdes-Sosa PA, Sanchez-Bornot JM, Sotero RC, Iturria-Medina Y, Aleman-Gomez Y, Bosch-Bayard J, et al. Model driven EEG/fMRI fusion of brain oscillations. Hum Brain Mapp [Internet]. 2009 Sep 15;30(9):2701–21. Available from: https://onlinelibrary.wiley.com/doi/10.1002/hbm.20704. 19107753 10.1002/hbm.20704 PMC6870602

[87] Palva JM, Wang SH, Palva S, Zhigalov A, Monto S, Brookes MJ, et al. Ghost interactions in MEG/EEG source space: A note of caution on inter-areal coupling measures. Neuroimage [Internet]. 2018 Jun;173(February):632–43. Available from: https://linkinghub.elsevier.com/retrieve/pii/S1053811918301290. 10.1016/j.neuroimage.2018.02.032 29477441

[88] Magistretti PJ, Allaman I. A Cellular Perspective on Brain Energy Metabolism and Functional Imaging. Neuron [Internet]. 2015 May;86(4):883–901. Available from: https://linkinghub.elsevier.com/retrieve/pii/S0896627315002597. doi: 10.1016/j.neuron.2015.03.035 25996133

[89] Jolivet R, Coggan JS, Allaman I, Magistretti PJ. Multi-timescale Modeling of Activity-Dependent Metabolic Coupling in the Neuron-Glia-Vasculature Ensemble. Graham L, editor. PLoS Comput Biol [Internet]. 2015 Feb;11(2):e1004036. Available from: https://dx.plos.org/10.1371/journal.pcbi.1004036. doi: 10.1371/journal.pcbi.1004036 25719367 PMC4342167

[90] Ritter P, Schirner M, McIntosh AR, Jirsa VK. The Virtual Brain Integrates Computational Modeling and Multimodal Neuroimaging. Brain Connect [Internet]. 2013 Apr;3(2):121–45. Available from: http://www.liebertpub.com/doi/10.1089/brain.2012.0120. 23442172 10.1089/brain.2012.0120 PMC3696923

[91] Amunts K, Lepage C, Borgeat L, Mohlberg H, Dickscheid T, Rousseau MÉ, et al. BigBrain: An Ultrahigh-Resolution 3D Human Brain Model. Science (1979) [Internet]. 2013 Jun 21;340(6139):1472–5. Available from: https://www.science.org/doi/ doi: 10.1126/science.1235381 23788795

[92] Arnatkevičiūtė A, Fulcher BD, Fornito A. A practical guide to linking brain-wide gene expression and neuroimaging data. Neuroimage [Internet]. 2019 Apr;189(July 2018):353–67. Available from: https://linkinghub.elsevier.com/retrieve/pii/S1053811919300114. doi: 10.1016/j.neuroimage.2019.01.011 30648605

[93] Frauscher B, von Ellenrieder N, Zelmann R, Doležalová I, Minotti L, Olivier A, et al. Atlas of the normal intracranial electroencephalogram: neurophysiological awake activity in different cortical areas. Brain [Internet]. 2018 Apr 1;141(4):1130–44. Available from: https://academic.oup.com/brain/article/141/4/1130/4915909. doi: 10.1093/brain/awy035 29506200

[94] Scimemi A. The Role of Astrocytes in Neurotransmitter Uptake and Brain Metabolism. In: De Pittà M, Berry H, editors. Computational Glioscience [Internet]. Springer, Cham; 2019. p. 309–28. Available from: http://link.springer.com/10.1007/978-3-030-00817-8_12

[95] Suárez LE, Markello RD, Betzel RF, Misic B. Linking Structure and Function in Macroscale Brain Networks. Trends Cogn Sci [Internet]. 2020 Apr;24(4):302–15. Available from: https://linkinghub.elsevier.com/retrieve/pii/S1364661320300267. doi: 10.1016/j.tics.2020.01.008 32160567

[96] Giaume C, Koulakoff A, Roux L, Holcman D, Rouach N. Astroglial networks: a step further in neuroglial and gliovascular interactions. Nat Rev Neurosci [Internet]. 2010 Feb;11(2):87–99. Available from: https://www.nature.com/articles/nrn2757. 10.1038/nrn2757 20087359

[97] Bernier M, Cunnane SC, Whittingstall K. The morphology of the human cerebrovascular system. Hum Brain Mapp [Internet]. 2018 Dec 28;39(12):4962–75. Available from: https://onlinelibrary.wiley.com/doi/10.1002/hbm.24337. 30265762 10.1002/hbm.24337 PMC6866388

[98] Schmahmann JD, Smith EE, Eichler FS, Filley CM. Cerebral White Matter. Ann N Y Acad Sci [Internet]. 2008 Oct 23;1142(1):266–309. Available from: https://nyaspubs.onlinelibrary.wiley.com/doi/10.1196/annals.1444.017. 18990132 10.1196/annals.1444.017 PMC3753195

[99] Wang HE, Triebkorn P, Breyton M, Dollomaja B, Lemarechal JD, Petkoski S, et al. Virtual brain twins: from basic neuroscience to clinical use. Natl Sci Rev [Internet]. 2024 Apr 3;11(5). Available from: doi: 10.1093/nsr/nwae079 38698901 PMC11065363

[100] Tourbier S, Rue-Queralt J, Glomb K, Aleman-Gomez Y, Mullier E, Griffa A, et al. Connectome Mapper 3: A Flexible and Open-Source Pipeline Software for Multiscale Multimodal Human Connectome Mapping. J Open Source Softw [Internet]. 2022 Jun 27;7(74):4248. Available from: https://joss.theoj.org/papers/10.21105/joss.04248.

[101] Van Essen DC, Smith SM, Barch DM, Behrens TEJ, Yacoub E, Ugurbil K. The WU-Minn Human Connectome Project: An overview. Neuroimage [Internet]. 2013 Oct;80:62–79. Available from: https://linkinghub.elsevier.com/retrieve/pii/S1053811913005351. doi: 10.1016/j.neuroimage.2013.05.041 23684880 PMC3724347

[102] Glasser MF, Sotiropoulos SN, Wilson JA, Coalson TS, Fischl B, Andersson JL, et al. The minimal preprocessing pipelines for the Human Connectome Project. Neuroimage [Internet]. 2013 Oct;80:105–24. Available from: https://linkinghub.elsevier.com/retrieve/pii/S1053811913005053. doi: 10.1016/j.neuroimage.2013.04.127 23668970 PMC3720813

[103] Yeh C, Jones DK, Liang X, Descoteaux M, Connelly A. Mapping Structural Connectivity Using Diffusion MRI: Challenges and Opportunities. Journal of Magnetic Resonance Imaging [Internet]. 2021 Jun 17;53(6):1666–82. Available from: https://onlinelibrary.wiley.com/doi/10.1002/jmri.27188. 32557893 10.1002/jmri.27188 PMC7615246

[104] Theaud G, Houde JC, Boré A, Rheault F, Morency F, Descoteaux M. TractoFlow: A robust, efficient and reproducible diffusion MRI pipeline leveraging Nextflow & Singularity. Neuroimage [Internet]. 2020 Sep;218 (April):116889. Available from: https://linkinghub.elsevier.com/retrieve/pii/S105381192030375X. doi: 10.1016/j.neuroimage.2020.116889 32447016

[105] St-Onge E, Daducci A, Girard G, Descoteaux M. Surface-enhanced tractography (SET). Neuroimage [Internet]. 2018 Apr;169(December 2017):524–39. Available from: https://linkinghub.elsevier.com/retrieve/pii/S1053811917310583. doi: 10.1016/j.neuroimage.2017.12.036 29258891

[106] Schiavi S, Ocampo-Pineda M, Barakovic M, Petit L, Descoteaux M, Thiran JP, et al. A new method for accurate in vivo mapping of human brain connections using microstructural and anatomical information. Sci Adv [Internet]. 2020 Jul 31;6(31):eaba8245. Available from: https://www.science.org/doi/10.1126/sciadv.aba8245. 32789176 10.1126/sciadv.aba8245 PMC7399649

[107] Fonov V, Evans AC, Botteron K, Almli CR, McKinstry RC, Collins DL. Unbiased average age-appropriate atlases for pediatric studies. Neuroimage [Internet]. 2011 Jan;54(1):313–27. Available from: https://linkinghub.elsevier.com/retrieve/pii/S1053811910010062. doi: 10.1016/j.neuroimage.2010.07.033 20656036 PMC2962759

[108] Fischl B. FreeSurfer. Neuroimage [Internet]. 2012 Aug;62(2):774–81. Available from: https://linkinghub.elsevier.com/retrieve/pii/S1053811912000389. doi: 10.1016/j.neuroimage.2012.01.021 22248573 PMC3685476

[109] MATLAB. Version 9.12.0 (R2022a). Natick, Massachusetts: The MathWorks Inc.; 2022.

[110] Corey DM, Dunlap WP, Burke MJ. Averaging Correlations: Expected Values and Bias in Combined Pearson r s and Fisher’s z Transformations. J Gen Psychol [Internet]. 1998 Jul;125(3):245–61. Available from: http://www.tandfonline.com/doi/abs/10.1080/00221309809595548.

[111] De Domenico M, Solé-Ribalta A, Omodei E, Gómez S, Arenas A. Ranking in interconnected multilayer networks reveals versatile nodes. Nat Commun [Internet]. 2015 Apr 23;6(1):6868. Available from: https://www.nature.com/articles/ncomms7868. doi: 10.1038/ncomms7868 25904405

[112] De Domenico M, Lancichinetti A, Arenas A, Rosvall M. Identifying Modular Flows on Multilayer Networks Reveals Highly Overlapping Organization in Interconnected Systems. Phys Rev X [Internet]. 2015 Mar 6;5(1):011027. Available from: https://link.aps.org/doi/10.1103/PhysRevX.5.011027.

[113] De Domenico M, Solé-Ribalta A, Cozzo E, Kivelä M, Moreno Y, Porter MA, et al. Mathematical Formulation of Multilayer Networks. Phys Rev X [Internet]. 2013 Dec 4;3(4):041022. Available from: https://link.aps.org/doi/10.1103/PhysRevX.3.041022.

[114] De Domenico M, Nicosia V, Arenas A, Latora V. Structural reducibility of multilayer networks. Nat Commun [Internet]. 2015 Apr 23;6(1):6864. Available from: https://www.nature.com/articles/ncomms7864. 10.1038/ncomms7864 25904309

[115] Crameri F, Shephard GE, Heron PJ. The misuse of colour in science communication. Nat Commun [Internet]. 2020 Oct 28;11(1):5444. Available from: https://www.nature.com/articles/s41467-020-19160-7. doi: 10.1038/s41467-020-19160-7 33116149 PMC7595127

