# Supplementary material for: Dialogue mechanisms between astrocytic and neuronal networks: A whole-brain modelling approach
Source: PLoS Comput Biol. 2025 Jan 13;21(1):e1012683. doi: 10.1371/journal.pcbi.1012683 (PMC11730384; doi:10.1371/journal.pcbi.1012683)
Supplement: S1 File — (PDF) [file pcbi.1012683.s001.pdf]

Supporting Information for "Dialogue mechanisms between astrocytic and neuronal networks: a whole-brain modelling approach"

Obaï Bin Ka'b Ali[1,2,*], Alexandre Vidal[3], Christophe Grova[4,5], Habib Benali[2,6]

1. Physics Department, Concordia University, Montreal, Canada
2. Electrical and Computer Engineering Department, Concordia University, Montreal, Canada
3. Laboratoire de Mathématiques et Modélisation d'Evry (LAMME), Université Evry, CNRS, Université Paris-Saclay, France
4. Multimodal Functional Imaging Lab, Department of Physics, Concordia School of Health, Concordia University, Montreal, Canada
5. Multimodal Functional Imaging Lab, Biomedical Engineering Department, McGill University, Montreal, Canada
6. INSERM U1146, Paris, France

* Corresponding author: ali.obaibk@gmail.com

Table of Contents

S1: Model variables and parameters 2

References 5

List of Tables

Table A. **Definitions of variables and abbreviations.** 2

Table B. **Simulation parameters.** 3

S1: Model variables and parameters

Table A. **Definitions of variables and abbreviations.** Units for variables are indicated in parentheses where applicable.

**Name**	**Description**
Pyr	Population of pyramidal cells
ExIn	Population of excitatory interneurons
InIn	Population of inhibitory interneurons
Ast	Population of astrocytes
Glu	Glutamate
GABA	Gamma-aminobutyric acid
E_{Pyr} (mV)	Average excitatory postsynaptic potentials from Pyr to ExIn and InIn
E_{ExIn} (mV)	Average excitatory postsynaptic potentials from ExIn to Pyr
E_{InIn} (mV)	Average inhibitory postsynaptic potentials from InIn to Pyr
LFP (mV)	Local field potentials, $\mathrm{LFP} \coloneqq E_{\mathrm{ExIn}} - E_{\mathrm{InIn}}$
J_{Glu} (μmol/s)	Extracellular Glu release rate of Pyr
$\mathrm{Glu_e}$ (μmol)	Extracellular Glu concentration
$\mathrm{Glu_{Ast}}$ (μmol)	Intracellular Glu concentration of Ast
J_{GABA} (μmol/s)	Extracellular GABA release rate of InIn
$\mathrm{GABA_e}$ (μmol)	Extracellular GABA concentration
$\mathrm{GABA_{Ast}}$ (μmol)	Intracellular GABA concentration of Ast
F_{Pyr} (Hz)	Firing rate from Pyr
F_{ExIn} (Hz)	Firing rate from ExIn
F_{InIn} (Hz)	Firing rate from InIn
v_{Pyr} (mV)	Excitability threshold of Pyr
v_{ExIn} (mV)	Excitability threshold of ExIn
v_{InIn} (mV)	Excitability threshold of InIn
v_{Glu} (mV)	Excitability threshold offset induced by $\mathrm{Glu_e}$ on Pyr and InIn
v_{GABA} (mV)	Excitability threshold offset induced by $\mathrm{GABA_e}$ on Pyr
Q_{Pyr} (Hz)	Neuronal network feedback on Pyr
$Q_{\mathrm{Glu}}^{\mathrm{Ast}}$ (Hz)	Astrocytic network feedback inducing Glu release from Pyr
$Q_{\mathrm{GABA}}^{\mathrm{Ast}}$ (Hz)	Astrocytic network feedback inducing GABA release from InIn

Table B. **Simulation parameters.** Parameters (40 nodal + 3 global + 2 structural connectivity matrices) are based on (Blanchard et al., 2016; Ferrat et al., 2018; Garnier et al., 2016), together with the analyses in sections *S2.1* and *S2.2* of *S2 File*, and sections *S4.1* and *S4.2* of *S4 File*. $\mathcal{N}$: normal distribution; SD: standard deviation; PSP: postsynaptic potentials; RTF: release transfer function.

Name	Description	Value
A	Tuning parameter of excitatory PSP maximal amplitude	$3.25\ \mathrm{mV}$
B	Tuning parameter of inhibitory PSP maximal amplitude	$22\ \mathrm{mV}$
a	Reciprocal of time constant of excitatory PSP	$100\ \mathrm{s}^{-1}$
b	Reciprocal of time constant of inhibitory PSP	$50\ \mathrm{s}^{-1}$
v_{max}	Maximal neuronal firing rate	$5\ \mathrm{Hz}$
r	Neuronal excitability rate	$0.56\ \mathrm{mV}^{-1}$
v_0	Baseline neuronal excitability threshold	$6\ \mathrm{mV}$
q	Baseline neuronal firing rate	$\mathcal{N}\begin{pmatrix}\mathrm{mean} = 240\ \mathrm{Hz} \\ \mathrm{SD} = 10\ \mathrm{Hz}\end{pmatrix}$
$C^{\mathrm{Pyr}\to\mathrm{ExIn}}$	Nodal synaptic connection strength from Pyr to ExIn	135
$C^{\mathrm{ExIn}\to\mathrm{Pyr}}$	Nodal synaptic connection strength from ExIn to Pyr	108
$C^{\mathrm{Pyr}\to\mathrm{InIn}}$	Nodal synaptic connection strength from Pyr to InIn	33.75
$C^{\mathrm{InIn}\to\mathrm{Pyr}}$	Nodal synaptic connection strength from InIn to Pyr	33.75
W	Tuning parameter of Glu RTF gain	$4.9\ \mu\mathrm{mol/s}$
w_{r}	Reciprocal of rise time constant of Glu RTF	$90\ \mathrm{s}^{-1}$
w_{d}	Reciprocal of decay time constant of Glu RTF	$33\ \mathrm{s}^{-1}$
$V_{\mathrm{Glu}}^{\mathrm{e}\to\mathrm{Ast}}$	Maximal astrocytic Glu uptake rate	$4.5\ \mu\mathrm{mol/s}$
$V_{\mathrm{Glu}}^{\mathrm{e}\to\mathrm{Pyr}}$	Maximal neuronal Glu uptake rate	$0.5\ \mu\mathrm{mol/s}$
$r_{\mathrm{Glu}}^{\mathrm{e}\to\mathrm{Ast,Pyr}}$	Rate parameter of Glu uptake rate sigmoid	$0.5\ \mu\mathrm{mol}^{-1}$
$\theta_{\mathrm{Glu}}^{\mathrm{e}\to\mathrm{Ast,Pyr}}$	Threshold parameter of Glu uptake rate sigmoid	$9\ \mu\mathrm{mol}$
$\tau_{\mathrm{Glu}}^{\mathrm{Ast}}$	Time constant of astrocytic Glu degradation	$1/9\ \mathrm{s}$
Z	Tuning parameter of GABA RTF gain	$50.6\ \mu\mathrm{mol/s}$
z_{r}	Reciprocal of rise time constant of GABA RTF	$90\ \mathrm{s}^{-1}$
z_{d}	Reciprocal of decay time constant of GABA RTF	$33\ \mathrm{s}^{-1}$
$V_{\mathrm{GABA}}^{\mathrm{e}\to\mathrm{Ast}}$	Maximal astrocytic GABA uptake rate	$2\ \mu\mathrm{mol/s}$
$V_{\mathrm{GABA}}^{\mathrm{e}\to\mathrm{InIn}}$	Maximal neuronal GABA uptake rate	$5\ \mu\mathrm{mol/s}$
$K_{\mathrm{GABA}}^{\mathrm{e}\to\mathrm{Ast}}$	Michaelis–Menten concentration parameter for astrocytic GABA uptake rate	$8\ \mu\mathrm{mol}$
$K_{\mathrm{GABA}}^{\mathrm{e}\to\mathrm{InIn}}$	Michaelis–Menten concentration parameter for neuronal GABA uptake rate	$24\ \mu\mathrm{mol}$
$\tau_{\mathrm{GABA}}^{\mathrm{Ast}}$	Time constant of astrocytic GABA degradation	$1/9\ \mathrm{s}$
$m_{\mathrm{Glu}}^{\mathrm{Pyr}}$	Maximal excitability threshold induced by $\mathrm{Glu_e}$ on Pyr	$0.8\ \mathrm{mV}$

Name	Description	Value
$r_{\mathrm{Glu}}^{\mathrm{Pyr,InIn}}$	Rate parameter of nodal $\mathrm{Glu_e}$ feedback sigmoid	$0.5\ \mu\mathrm{mol}^{-1}$
$\theta_{\mathrm{Glu}}^{\mathrm{Pyr,InIn}}$	Threshold parameter of nodal $\mathrm{Glu_e}$ feedback sigmoid	$10\ \mu\mathrm{mol}$
$\delta_{\mathrm{Glu}}^{\mathrm{Pyr}}$	Baseline shift parameter of nodal $\mathrm{Glu_e}$ feedback sigmoid	0.4 mV
$\mu_{\mathrm{Glu}}^{\mathrm{InIn/Pyr}}$	Maximal excitability threshold induced by $\mathrm{Glu_e}$ on InIn divided by $m_{\mathrm{Glu}}^{\mathrm{Pyr}}$	0.5
$m_{\mathrm{GABA}}^{\mathrm{Pyr}}$	Maximal excitability threshold induced by $\mathrm{GABA_e}$ on Pyr	4.2 mV
$r_{\mathrm{GABA}}^{\mathrm{Pyr}}$	Rate parameter of nodal $\mathrm{GABA_e}$ feedback sigmoid	$0.25\ \mu\mathrm{mol}^{-1}$
$\theta_{\mathrm{GABA}}^{\mathrm{Pyr}}$	Threshold parameter of nodal $\mathrm{GABA_e}$ feedback sigmoid	$20\ \mu\mathrm{mol}$
$\delta_{\mathrm{GABA}}^{\mathrm{Pyr}}$	Baseline shift parameter of nodal $\mathrm{GABA_e}$ feedback sigmoid	2.1 mV
ω_{Pyr}	Gain of neuronal network feedback	7.5
ω_{Glu}	Diffusion coefficient for Glu-induced astrocytic network feedback on Glu release rates	$[2.90; 6.47]\ \mu\mathrm{mol}^{-1}$
ω_{GABA}	Diffusion coefficient for Glu-induced astrocytic network feedback on GABA release rates	$[0.14; 1.94]\ \mu\mathrm{mol}^{-1}$
$m_{\mathrm{Glu}}^{\mathrm{Ast}}$	Maximal amplitude of Glu-induced astrocytic network feedback sigmoid	$4.5\ \mu\mathrm{mol/s}$
$r_{\mathrm{Glu}}^{\mathrm{Ast}}$	Rate parameter of Glu-induced astrocytic network feedback sigmoid	$0.5\ \mu\mathrm{mol}^{-1}$
$\theta_{\mathrm{Glu}}^{\mathrm{Ast}}$	Threshold parameter of Glu-induced astrocytic network feedback sigmoid	$9\ \mu\mathrm{mol}$
Ω_{Pyr}	Network connectivity weights between Pyr	$[0; 1]$
Ω_{Ast}	Network connectivity weights between Ast	$[0; 1]$

References

Blanchard, S., Saillet, S., Ivanov, A., Benquet, P., Bénar, C.-G., Pélégrini-Issac, M., Benali, H., & Wendling, F. (2016). A New Computational Model for Neuro-Glio-Vascular Coupling: Astrocyte Activation Can Explain Cerebral Blood Flow Nonlinear Response to Interictal Events. *PLOS ONE*, *11*(2), e0147292. https://doi.org/10.1371/journal.pone.0147292

Ferrat, L. A., Goodfellow, M., & Terry, J. R. (2018). Classifying dynamic transitions in high dimensional neural mass models: A random forest approach. *PLOS Computational Biology*, *14*(3), e1006009. https://doi.org/10.1371/journal.pcbi.1006009

Garnier, A., Vidal, A., & Benali, H. (2016). A Theoretical Study on the Role of Astrocytic Activity in Neuronal Hyperexcitability by a Novel Neuron-Glia Mass Model. *The Journal of Mathematical Neuroscience*, *6*(1), 10. https://doi.org/10.1186/s13408-016-0042-0
