# Supplementary material for: Dialogue mechanisms between astrocytic and neuronal networks: A whole-brain modelling approach
Source: PLoS Comput Biol. 2025 Jan 13;21(1):e1012683. doi: 10.1371/journal.pcbi.1012683 (PMC11730384; doi:10.1371/journal.pcbi.1012683)
Supplement: S2 File — (PDF) [file pcbi.1012683.s002.pdf]

Supporting Information for "Dialogue mechanisms between astrocytic and neuronal networks: a whole-brain modelling approach"

Obaï Bin Ka'b Ali[1,2,*], Alexandre Vidal[3], Christophe Grova[4,5], Habib Benali[2,6]

1. Physics Department, Concordia University, Montreal, Canada
2. Electrical and Computer Engineering Department, Concordia University, Montreal, Canada
3. Laboratoire de Mathématiques et Modélisation d'Evry (LAMME), Université Evry, CNRS, Université Paris-Saclay, France
4. Multimodal Functional Imaging Lab, Department of Physics, Concordia School of Health, Concordia University, Montreal, Canada
5. Multimodal Functional Imaging Lab, Biomedical Engineering Department, McGill University, Montreal, Canada
6. INSERM U1146, Paris, France

* Corresponding author: ali.obaibk@gmail.com

Table of Contents

S2: Constraining dynamical regimes 2

S2.1 Network model parameterization through bifurcation analysis 2
S2.2 Exploration parameter space 7
S2.3 Summary and generalization 9
S2.4 Simulated time series 10

References 12

List of Figures

Fig A. **Two-parameter bifurcation diagrams.** 3
Fig B. **Two-parameter bifurcation diagram.** 5
Fig C. **Simulation parameter planes.** 7
Fig D. **Simulated mean states.** 8
Fig E. **Physiological whole-brain network simulation, bridging electrophysiology and neurotransmission.** 10

List of Tables

Table A. **Descriptive statistics of energy distribution across frequency bands for extracellular glutamate and GABA dynamics.** 11

S2: Constraining dynamical regimes

In this study, all bifurcation analyses were conducted numerically, primarily using *MatCont* (Dhooge et al., 2008), version 7.3 available at https://gitlab.utwente.nl/m7686441/matcont, and *PyDSTool* (Clewley, 2012), version 0.91.0 accessible at https://github.com/robclewley/pydstool.

S2.1 Network model parameterization through bifurcation analysis

Constraining LFP is facilitated by leveraging the neuronal compartment of our network model, which extends the Jansen–Rit model (Jansen & Rit, 1995). The Jansen–Rit model is well-regarded for replicating biologically plausible neuronal population activities. It has been effectively used in numerous studies to mirror essential features of electrophysiological recordings, notably the alpha band oscillatory patterns seen during rest (Griffiths et al., 2022). In contrast, our understanding of the astrocytic and extracellular compartments in the network model is based on more recent studies (Blanchard et al., 2016; Garnier et al., 2016). The primary challenge here is the scarcity of methodologies or empirical data for fine-tuning these model aspects (De Pittà & Berry, 2019; Kastanenka et al., 2020). However, bifurcation analysis at the nodal level provides a feasible method for setting nodal parameters within realistic bounds, while accommodating network feedback terms (Garnier et al., 2016).

The core concept of our parameterization is built around two key observations within the nodal neuron-astrocyte mass model: firstly, the neuronal subsystem exhibits dynamics on a much shorter time scale compared to other subsystems, enabling the application of time scale separation techniques; secondly, the neuronal subsystem is influenced exclusively by the variations in $\mathrm{Glu_e}$ and $\mathrm{GABA_e}$, which occur on significantly slower time scales compared to neuronal firing rates (further discussed in section *S2.4*), affecting neuronal excitability levels through v_{Glu} and v_{GABA}. By utilizing a quasi-steady-state approximation given the slow changes in $\mathrm{Glu_e}$ and $\mathrm{GABA_e}$, we can simplify the mass model to focus on the neuronal compartment. This reduction allows us to conduct a bifurcation analysis, exploring how changes in v_{Glu} and v_{GABA} can lead to qualitative changes in neuronal dynamics. Moreover, by adopting a scenario where identical nodes are interconnected, forming a *homogeneous* network (where the values of $F_{\mathrm{Pyr}_{[n]}}$ are equal for all n), the neuronal network feedback simplifies to $Q_{\mathrm{Pyr}_{[n]}} = \omega_{\mathrm{Pyr}} F_{\mathrm{Pyr}_{[n]}}$, since the matrix Ω_{Pyr} has rows that all sum to one. In this homogeneous setup, the global neuronal coupling parameter ω_{Pyr} effectively acts as a nodal self-feedback parameter on pyramidal cells. Consequently, within such a framework, the values of ω_{Pyr} can be determined by examining the dynamics of an individual node, thereby circumventing the complexities associated with bifurcation analyses in a *heterogeneous*, high-dimensional nonlinear network model, an ongoing mathematical challenge.

Sourcing physiologically plausible set of scalar parameters from the existing literature, allows us to focus on the global network coupling parameters ω_{Pyr}, ω_{Glu}, and ω_{GABA}, along with the following nodal parameters: q, $m_{\mathrm{Glu}}^{\mathrm{Pyr}}$, $r_{\mathrm{Glu}}^{\mathrm{Pyr,InIn}}$, $\theta_{\mathrm{Glu}}^{\mathrm{Pyr,InIn}}$, $\delta_{\mathrm{Glu}}^{\mathrm{Pyr}}$, $m_{\mathrm{GABA}}^{\mathrm{Pyr}}$, $r_{\mathrm{GABA}}^{\mathrm{Pyr}}$, $\theta_{\mathrm{GABA}}^{\mathrm{Pyr}}$, $\delta_{\mathrm{GABA}}^{\mathrm{Pyr}}$, W, and Z. In this study, all parameters except for ω_{Glu}, ω_{GABA}, and q are fixed at specific scalar values.

The singular points of the neuronal compartment are then computed numerically using Equation *(A)*, given a value E_{Pyr} within the interval $]0; v_{\max} A/a[$:

$$q - \frac{a}{A} v_{\mathrm{GABA}} - \frac{a}{A}\left(v_0 - v_{\mathrm{Glu}} - \frac{1}{r}\ln\left(\frac{A v_{\max}}{a E_{\mathrm{Pyr}}} - 1\right) - \frac{A}{a} C^{\mathrm{ExIn}\to\mathrm{Pyr}} S\left(C^{\mathrm{Pyr}\to\mathrm{ExIn}} E_{\mathrm{Pyr}}, v_{\max}, r, v_0, 0\right) - \omega_{\mathrm{Pyr}} E_{\mathrm{Pyr}} + \frac{B}{b} C^{\mathrm{InIn}\to\mathrm{Pyr}} S\left(C^{\mathrm{Pyr}\to\mathrm{InIn}} E_{\mathrm{Pyr}}, v_{\max}, r, v_0 - \mu_{\mathrm{Glu}}^{\mathrm{InIn/Pyr}} v_{\mathrm{Glu}}, 0\right)\right) = 0 \tag{A}$$

From Equation *(A)*, we observe that v_{GABA} and q linearly combine, through the constant a/A. This linear combination facilitates a comprehensive understanding of the neuronal compartment's dynamics within the nodal mass model by enabling the construction of a codimension-2 bifurcation diagram with v_{Glu} and $p = q - v_{\mathrm{GABA}}\, a/A$ as parameters. It is important to note that negative values for p become biologically plausible, even when q and v_{GABA} remain positive. Moreover, if an additional excitatory feedback term was introduced on inhibitory cells (e.g., through long-range pyramidal projections), an additional term proportional to $(aB)/(Ab)$ would sum linearly with p, further influencing the inhibitory dynamics. Additionally, the Jansen–Rit model is retrieved, with q as its usual bifurcation parameter, when $v_{\mathrm{Glu}} = v_{\mathrm{GABA}} = 0$ mV and $\omega_{\mathrm{Pyr}} = 0$. Our approach thus reinterprets the bifurcation features of the Jansen–Rit model from a broader perspective, focusing on the influences of glutamatergic and GABAergic neurotransmissions. This bifurcation analysis is simplified by keeping q constant along the p-axis and allowing v_{GABA} to account fully for the variations of p, without loss of generality.

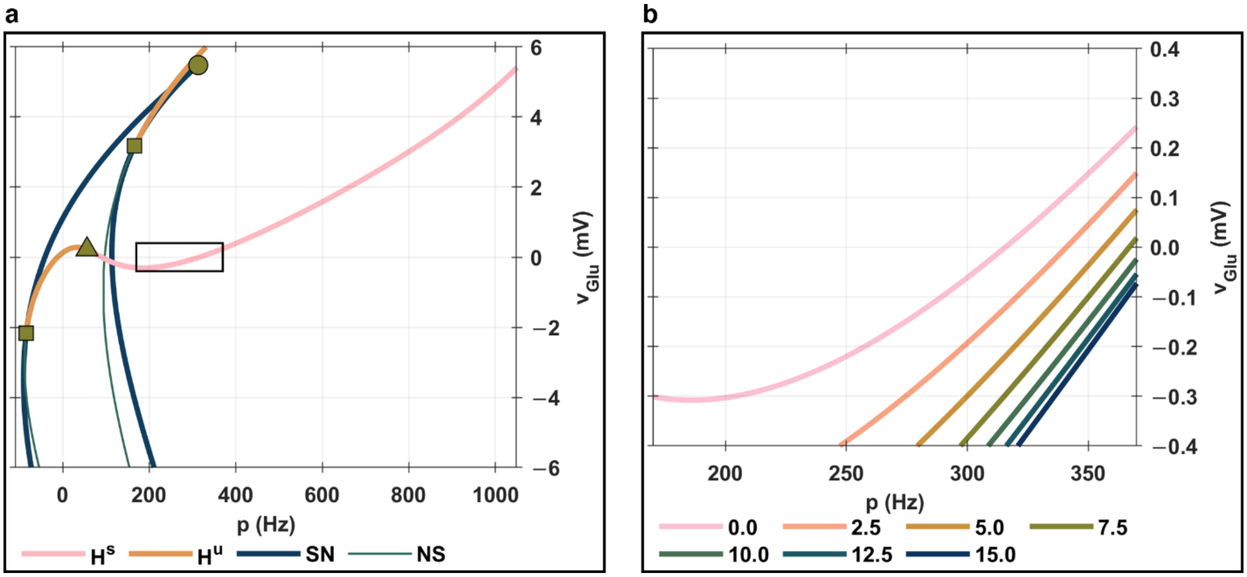

Fig A. **Two-parameter bifurcation diagrams.** In panel **(a)**, drawn with $\omega_{\mathrm{Pyr}} = 0$, $\mathrm{H^s}$ refers to supercritical Poincaré–Andronov–Hopf points, $\mathrm{H^u}$ refers to subcritical Hopf points, SN refers to saddle-node points, and NS refers to neutral saddle points (i.e., points associated with two real eigenvalues summing to zero). Saddle-node branches meet at a cusp bifurcation point represented as a circular green dot; Hopf branches meet at a Bautin (generalized Hopf) bifurcation point represented as a triangular green dot; and saddle-node, Hopf, and neutral saddle branches connect at Bogdanov–Takens bifurcation points represented as rectangular green dots. The rectangular black border highlights the dynamic regimes under consideration, where $p \in [170; 370]$ Hz and $v_{\mathrm{Glu}} \in [-0.4; 0.4]$ mV. In panel **(b)**, branches of supercritical Hopf bifurcations are drawn within the $(v_{\mathrm{Glu}}; p)$ plane for $\omega_{\mathrm{Pyr}} \in \{0; 2.5; 5; 7.5; 10; 12.5; 15\}$.

In the bifurcation diagram shown in Fig A(a), the specified ranges for v_{Glu} and p are designed to confine the neuronal compartment of the nodal model to primarily exhibit stable periodic orbits, particularly when behaving independently from its network counterparts. These orbits are strategically maintained at a distance from the regions associated with saddle nodes, bi-stability, and cusp catastrophes, thereby avoiding *epileptic-like* oscillatory activities while promoting what can be characterized as *physiological* oscillatory behaviors. Furthermore, these ranges encompass the traditional Jansen–Rit parametrization (when $v_{\text{Glu}} = 0$ mV), and they ensure that both glutamatergic pyramidal and GABAergic inhibitory neuron populations operate closer to the linear segments of their sigmoidal firing rate functions, which is of biophysical interest.

The settings for $m_{\text{Glu}}^{\text{Pyr}}$, $r_{\text{Glu}}^{\text{Pyr,InIn}}$, $\theta_{\text{Glu}}^{\text{Pyr,InIn}}$, $\delta_{\text{Glu}}^{\text{Pyr}}$, $m_{\text{GABA}}^{\text{Pyr}}$, $r_{\text{GABA}}^{\text{Pyr}}$, $\theta_{\text{GABA}}^{\text{Pyr}}$, and $\delta_{\text{GABA}}^{\text{Pyr}}$ are established by selecting physiologically realistic ranges for the excitability thresholds v_{Pyr} and v_{InIn}, and the extracellular neurotransmitter concentrations Glu_{e} and GABA_{e}. We opted for the interval [3.5; 8.5] mV for both v_{Pyr} and v_{InIn} (Ferrat et al., 2018), and established approximate concentration ranges of [5; 15] µmol for Glu_{e} and [5; 35] µmol for GABA_{e} (Blanchard et al., 2016). The validity of the chosen intervals for Glu_{e} and GABA_{e} is rationalized by analyzing the uptake rate functions $\text{Glu}_{\text{e}} \mapsto \mathrm{S}\left(\text{Glu}_{\text{e}}, V_{\text{Glu}}^{\text{e}\to\text{Ast}} + V_{\text{Glu}}^{\text{e}\to\text{Pyr}}, r_{\text{Glu}}^{\text{e}\to\text{Ast,Pyr}}, \theta_{\text{Glu}}^{\text{e}\to\text{Ast,Pyr}}, 0\right)$ and $\text{GABA}_{\text{e}} \mapsto \mathrm{H}\left(\text{GABA}_{\text{e}}, V_{\text{GABA}}^{\text{e}\to\text{Ast}}, K_{\text{GABA}}^{\text{e}\to\text{Ast}}\right) + \mathrm{H}\left(\text{GABA}_{\text{e}}, V_{\text{GABA}}^{\text{e}\to\text{InIn}}, K_{\text{GABA}}^{\text{e}\to\text{InIn}}\right)$. For Glu_{e}, the range [5; 15] µmol aligns primarily with the linear portion of the Glu_{e} uptake sigmoidal functions (rather than their saturation regions) where the nodal model most effectively equilibrates release and uptake rates. Similarly, the selected [5; 35] µmol range for GABA_{e} represents the optimal zone for balancing release and uptake rates. Although the GABA_{e} uptake functions are defined by rational polynomials, allowing for potentially higher upper bounds, setting these bounds excessively high could prolong simulation durations unless initial conditions are precisely defined to expedite equilibriums between neurotransmitter releases and uptakes.

The specified range for v_{Pyr} and v_{InIn} facilitated straightforward constraints for $m_{\text{Glu}}^{\text{Pyr}}$, $\delta_{\text{Glu}}^{\text{Pyr}}$, $m_{\text{GABA}}^{\text{Pyr}}$, and $\delta_{\text{GABA}}^{\text{Pyr}}$, as elucidated by the following inequalities:

$$\begin{gathered}(v_0 - 8.5)/\mu_{\text{Glu}}^{\text{InIn/Pyr}} \leq v_{\text{Glu}} \leq (v_0 - 3.5)/\mu_{\text{Glu}}^{\text{InIn/Pyr}} \\ 3.5 + v_{\text{Glu}}^{\text{sup}} - v_0 \leq v_{\text{GABA}} \leq 8.5 + v_{\text{Glu}}^{\text{inf}} - v_0 \\ 0 \leq v_{\text{Glu}}^{\text{sup}} - v_{\text{Glu}}^{\text{inf}} \leq 5\end{gathered} \tag{B}$$

where $v_{\text{Glu}}^{\text{inf}}$ and $v_{\text{Glu}}^{\text{sup}}$ denote the infimum and supremum values of v_{Glu}, respectively.

For instance, setting $-v_{\text{Glu}}^{\text{inf}} = v_{\text{Glu}}^{\text{sup}} = 0.4$ mV, such that $v_{\text{Glu}} \in [-0.4; 0.4]$ mV, results in $v_{\text{GABA}} \in [-2.1; 2.1]$ mV, or equivalently $v_{\text{GABA}}\, a/A \in [-65; 65]$ Hz, which subsequently restricts q to fall within [235; 305] Hz, given the p constraint of [170; 370] Hz. Notably, widening the interval for v_{Glu} results in a corresponding narrowing of the range for v_{GABA}. This wider range for v_{GABA} relative to v_{Glu} is intended to capture typical behaviors observed in many Jansen–Rit-based studies, which frequently explore variations along the p-axis.

With these ranges established the parameters for v_{Glu} and v_{GABA} are set as follows: $m_{\text{Glu}}^{\text{Pyr}} = 0.8$ mV, $r_{\text{Glu}}^{\text{Pyr,InIn}} = 0.5\ \mu\text{mol}^{-1}$, $\theta_{\text{Glu}}^{\text{Pyr,InIn}} = 10$ µmol, $\delta_{\text{Glu}}^{\text{Pyr}} = 0.4$ mV, $m_{\text{GABA}}^{\text{Pyr}} = 4.2$ mV, $r_{\text{GABA}}^{\text{Pyr}} = 0.25\ \mu\text{mol}^{-1}$, $\theta_{\text{GABA}}^{\text{Pyr}} = 20$ µmol, and $\delta_{\text{GABA}}^{\text{Pyr}} = 2.1$ mV (see also *Table B* in *S1 File*).

The bifurcation diagram shown in Fig A(a) was initially constructed under the assumption of fully independent identical nodes. However, this diagram can be expanded by incorporating the effects of the parameter ω_{Pyr}, which facilitates an exploration of network interactions between nodes within the limited context of a homogeneous network.

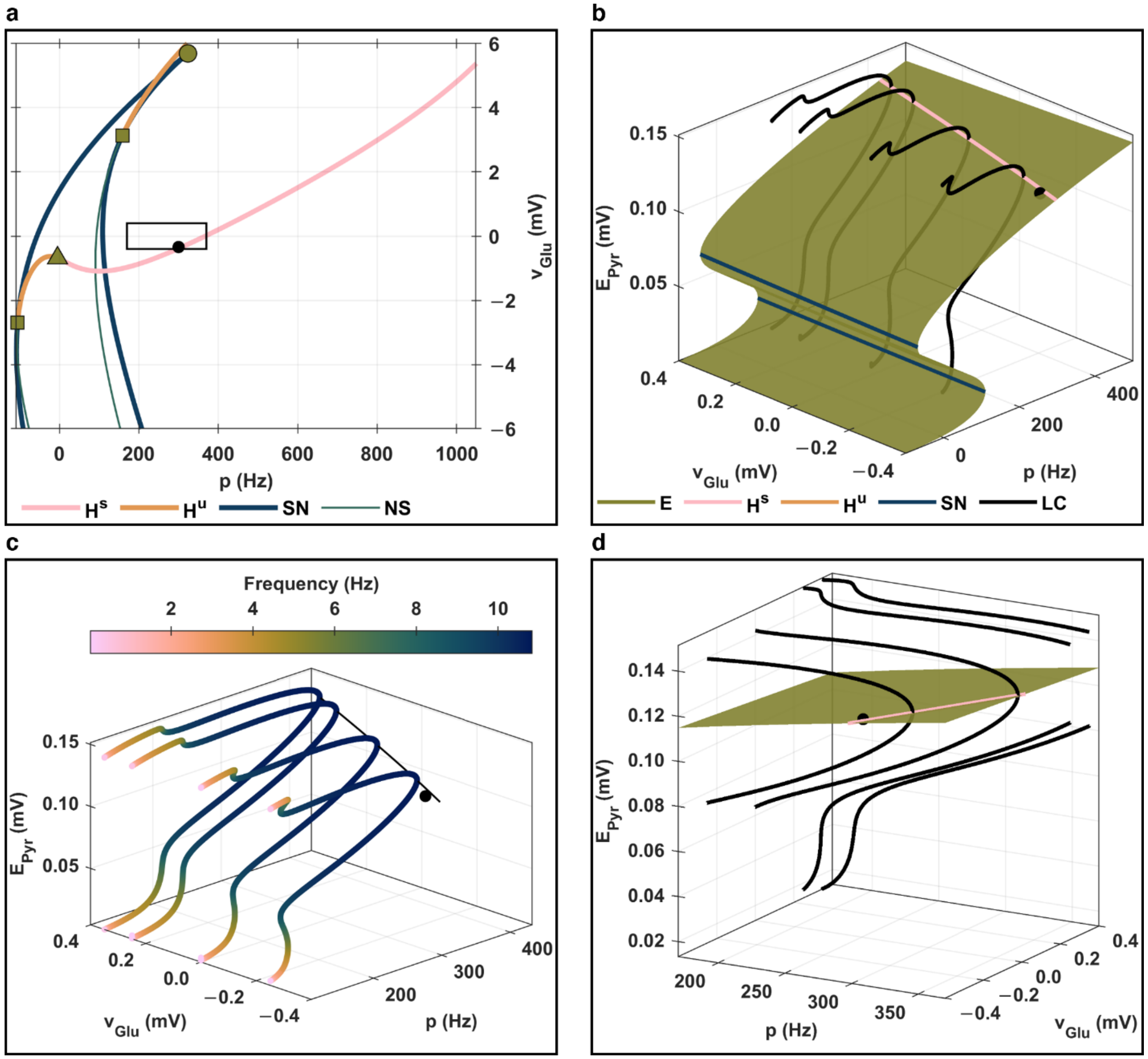

Fig B. **Two-parameter bifurcation diagram.** Drawn with $\omega_{\mathrm{Pyr}} = 7.5$. Panel **(a)**: $\mathrm{H^s}$ (supercritical Hopf points); $\mathrm{H^u}$ (subcritical Hopf points); SN (saddle-node points); NS (neutral saddle points); circular green dot (cusp bifurcation point); triangular green dot (Bautin bifurcation point); rectangular green dot (Bogdanov–Takens bifurcation point); rectangular black border (dynamic regimes under consideration, where $p \in [170; 370]$ Hz and $v_{\mathrm{Glu}} \in [-0.4; 0.4]$ mV). In panel **(b)**, a three-dimensional view of the diagram in panel **(a)** is shown, maintaining the range $v_{\mathrm{Glu}} \in [-0.4; 0.4]$ mV. The green surface represents singular points (E), the black solid lines represent maximal and minimal E_{Pyr} values along limit cycles (LC), and the other curves are the same as in panel **(a)**. Limit cycle curves are represented only for $v_{\mathrm{Glu}} \in \{-0.25; 0; 0.25; 0.35\}$ mV for clarity, and neutral saddle curves are not drawn because they do not have a dynamic meaning for general equilibria. In panel **(c)**, the same limit cycles from panel **(b)** are displayed alongside their respective frequencies, and the black solid thin lines represent Hopf bifurcations. Panel **(d)** magnifies the area within the black rectangular outline from panel **(a)**, focusing on p values between 170 and 370 Hz, to provide a closer examination of the selected dynamic regimes. This panel counts also as a magnification of panel

(b). The circular black dot in panels **(a)–(d)** represent the initial state selected for this study (as detailed in the accompanying text).

In the context of this expanded framework, the parameter plane highlighted earlier in Fig A(a), where $v_{\mathrm{Glu}} \in [-0.4; 0.4]$ mV and $p \in [170; 370]$ Hz, presents notable dynamics. As illustrated in Fig A(b), varying ω_{Pyr} maintains a supercritical Hopf bifurcation branch that shifts as ω_{Pyr} increases. This bifurcation branch divides the parameter space into two distinct regions: an oscillatory region that expands as ω_{Pyr} increases, characterized by stable periodic orbits, and a contracting non-oscillatory region marked by stable equilibria. The specific selection of ω_{Pyr}, was informed by the equilibria landscape depicted in Fig A(a) and was also based on the previous range analysis for q. From Equation *(A)*, the equilibria landscapes, whether ω_{Pyr} is zero or not, are linked by a shift along the p-axis, transforming $(E_{\mathrm{Pyr}}; p; v_{\mathrm{Glu}})$ to $(E_{\mathrm{Pyr}}; p + \omega_{\mathrm{Pyr}} E_{\mathrm{Pyr}}\, a/A\,; v_{\mathrm{Glu}})$. Since it was determined previously that q could range from 235 Hz to 305 Hz, by setting $q = 240$ Hz, we ensured that $p + Q_{\mathrm{Pyr}} = p + \omega_{\mathrm{Pyr}} E_{\mathrm{Pyr}}\, a/A$ remained within the [170; 370] Hz interval, by assessing the bounds on E_{Pyr}, leading to $\omega_{\mathrm{Pyr}} \leq (370 - q + \inf(v_{\mathrm{GABA}}\, a/A))/\sup(E_{\mathrm{Pyr}}\, a/A) \approx 16.7$. We selected $\omega_{\mathrm{Pyr}} = 7.5$ as in Fig B.

Upon setting $\omega_{\mathrm{Pyr}} = 7.5$, we defined an *initial* state for the simulations of the *Main Manuscript*. This state is illustrated in Fig B as a circular black dot near a supercritical Hopf bifurcation locus, where the influence of astrocytic network activity on the *baseline* levels of $\mathrm{Glu_e}$ and $\mathrm{GABA_e}$, compared to neuronal activity, is minimal. Specifically, the initial conditions were set as ${\omega_{\mathrm{Glu}}}^{\mathrm{initial}} = {\omega_{\mathrm{GABA}}}^{\mathrm{initial}} = 0.01\ \mu\mathrm{mol}^{-1}$, ${v_{\mathrm{Glu}}}^{\mathrm{initial}} = -0.34$ mV (equivalently, ${\mathrm{Glu_e}}^{\mathrm{initial}} \approx 4.98$ μmol), and ${v_{\mathrm{GABA}}}^{\mathrm{initial}} = -1.95$ mV (equivalently, ${\mathrm{GABA_e}}^{\mathrm{initial}} \approx 6.82$ μmol).

The insignificance of astrocytic network activity contributions relative to neuronal activity at this initial state is underscored by the following inequalities:

$$\begin{aligned} {\omega_{\mathrm{Glu}}}^{\mathrm{initial}} \mathrm{S}\left({\mathrm{Glu_e}}^{\mathrm{initial}}, m_{\mathrm{Glu}}^{\mathrm{Ast}}, r_{\mathrm{Glu}}^{\mathrm{Ast}}, \theta_{\mathrm{Glu}}^{\mathrm{Ast}}, 0\right) &\coloneqq {Q_{\mathrm{Glu}}^{\mathrm{Ast}}}^{\mathrm{initial}} \ll {F_{\mathrm{Pyr}}}^{\mathrm{initial}} \\ {\omega_{\mathrm{GABA}}}^{\mathrm{initial}} \mathrm{S}\left({\mathrm{Glu_e}}^{\mathrm{initial}}, m_{\mathrm{Glu}}^{\mathrm{Ast}}, r_{\mathrm{Glu}}^{\mathrm{Ast}}, \theta_{\mathrm{Glu}}^{\mathrm{Ast}}, 0\right) &\coloneqq {Q_{\mathrm{GABA}}^{\mathrm{Ast}}}^{\mathrm{initial}} \ll {F_{\mathrm{InIn}}}^{\mathrm{initial}} \end{aligned} \tag{C}$$

The establishment of an initial state also led to fixed values for W and Z. These calculated W and Z values are instrumental in sustaining the selected baseline concentrations at the initial state, factoring in the contributions from non-zero astrocytic network activity. The values were determined through steady-state calculations under the assumption that neurotransmitter uptake and release rates are in equilibrium:

$$\begin{aligned} W &= w_{\mathrm{d}} \frac{\mathrm{S}\left({\mathrm{Glu_e}}^{\mathrm{initial}}, V_{\mathrm{Glu}}^{\mathrm{e\to Ast}} + V_{\mathrm{Glu}}^{\mathrm{e\to Pyr}}, r_{\mathrm{Glu}}^{\mathrm{e\to Ast,Pyr}}, \theta_{\mathrm{Glu}}^{\mathrm{e\to Ast,Pyr}}, 0\right)}{{F_{\mathrm{Pyr}}}^{\mathrm{initial}} + {Q_{\mathrm{Glu}}^{\mathrm{Ast}}}^{\mathrm{initial}}} \approx 4.9\ \mu\mathrm{mol/s} \\ Z &= z_{\mathrm{d}} \frac{\mathrm{H}\left({\mathrm{GABA_e}}^{\mathrm{initial}}, V_{\mathrm{GABA}}^{\mathrm{e\to Ast}}, K_{\mathrm{GABA}}^{\mathrm{e\to Ast}}\right) + \mathrm{H}\left({\mathrm{GABA_e}}^{\mathrm{initial}}, V_{\mathrm{GABA}}^{\mathrm{e\to InIn}}, K_{\mathrm{GABA}}^{\mathrm{e\to InIn}}\right)}{{F_{\mathrm{InIn}}}^{\mathrm{initial}} + {Q_{\mathrm{GABA}}^{\mathrm{Ast}}}^{\mathrm{initial}}} \\ &\approx 50.6\ \mu\mathrm{mol/s} \end{aligned} \tag{D}$$

To conclude, the parameter setting defined in this section *S2.1* were designed to enable the emergence of chimera states and metastable synchrony, where nodes display transient and partially synchronized behaviors. Specifically, the strength of the global neuronal network coupling

parameter, ω_{Pyr}, was calibrated to ensure measurable amplitude and phase network synchronizations. Yet, ω_{Pyr} was kept low enough to prevent nodes from exhibiting behaviors that were either too homogeneous or too independent compared to their stochastic baseline neuronal firing rates q. These baseline rates were sampled from a normal distribution with a mean and standard deviation of 240 ± 10 Hz.

S2.2 Exploration parameter space

The preceding section *S2.1* refined the parameterization of our network model to focus on the two global astrocytic network coupling parameters: ω_{Glu} and ω_{GABA}. To effectively explore these parameters, we established a biologically relevant grid for $(\omega_{\text{Glu}}; \omega_{\text{GABA}})$ by sampling the domain defined by $(v_{\text{Glu}}; v_{\text{GABA}}) \in [-0.300; 0.150] \times [-1.625; 1.625]$ mV × mV using a 35×35 uniform grid. This gridding approach allowed $(\omega_{\text{Glu}}; \omega_{\text{GABA}})$ to take values within $[2.90; 6.47] \times [0.14; 1.94]$ $\mu\text{mol}^{-1} \times \mu\text{mol}^{-1}$, derived through steady-state calculations.

Consequently, an increase in ω_{Glu} typically resulted in an elevation of Glu_{e} from its baseline up to a maximum of 15 µmol, predominantly influencing the v_{Glu}-axis. Likewise, an elevation in ω_{GABA} generally raised GABA_{e} from its baseline to a maximum of 35 µmol, primarily impacting the p-axis or v_{GABA}-axis.

This parameterization produced a grid comprising 1225 unique $(\omega_{\text{Glu}}; \omega_{\text{GABA}})$ pairings, which were utilized for the simulations of the *Main Manuscript*. Fig C(a) graphically represents the simulation parameter plane defined by these 1225 unique pairs. Fig C(b) displays the uniform grid on the parameter plane defined by $(v_{\text{Glu}}; v_{\text{GABA}})$, which was used for deriving the specific $(\omega_{\text{Glu}}; \omega_{\text{GABA}})$ pairings. As discussed below, despite the relatively sparser sampling of lower ω_{GABA} values compared to higher ones, additional simulations targeting lower ω_{GABA} values were not deemed beneficial for the aims of this study.

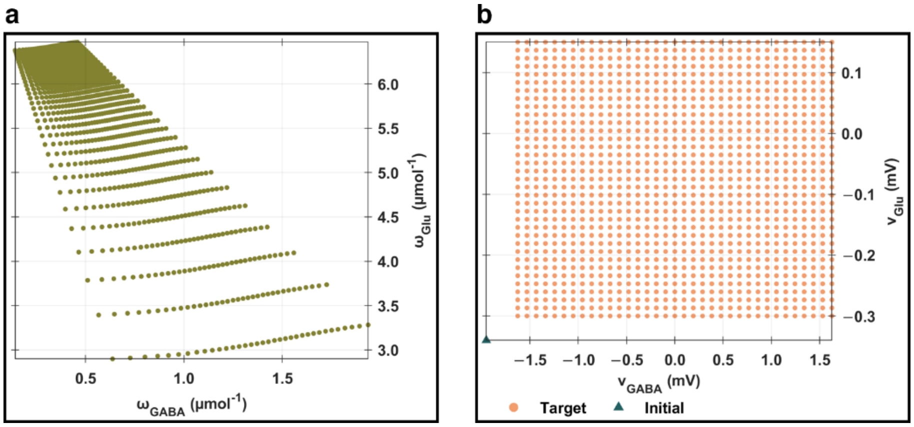

Fig C. **Simulation parameter planes. (a)** Simulation parameter plane defined by $(\omega_{\text{Glu}}; \omega_{\text{GABA}})$. **(b)** The underlying parameter plane from which the panel **(a)** was derived. The green triangular dot (on the bottom left corner) represents the initial state chosen for this study. To clarify, this initial state corresponds to $\omega_{\text{Glu}}{}^{\text{initial}} = \omega_{\text{GABA}}{}^{\text{initial}} = 10^{-2}$ μmol^{-1}, although it is not drawn in panel **(a)**.

Fig D(a) illustrates, in the plane $(v_{\text{Glu}}; v_{\text{GABA}})$, the whole-brain and regional mean states over the last ten seconds of the calibrated simulations, while Fig D(b) displays these mean states as calculated from the ten simulation batches. To clarify, before conducting the ten simulation batches that underpinned the analyses in the *Main Manuscript*, a single calibrated simulation lasting 370 seconds was run for each parameter pair $(\omega_{\text{Glu}}; \omega_{\text{GABA}})$. Each of these simulations was visually

inspected to confirm that steady states were achieved in the final ten seconds. These last ten seconds of the calibrated simulations were then used to set the initial conditions for all subsequent simulations across the ten batches.

Notably, the network states illustrated in Fig D(a) and Fig D(b) bear a striking resemblance to the target states shown in Fig C(b), except in regions of the parameter plane characterized by high values of both v_{Glu} and v_{GABA}. This observation suggests that the constraints applied to the network model were effective in aligning the simulated states with the desired target states, except under conditions of high astrocytic network coupling strengths where small deviations were noted. Given the 120-second duration of the simulations across the ten batches, less variance in regional states was anticipated when comparing Fig D(b) to Fig D(a).

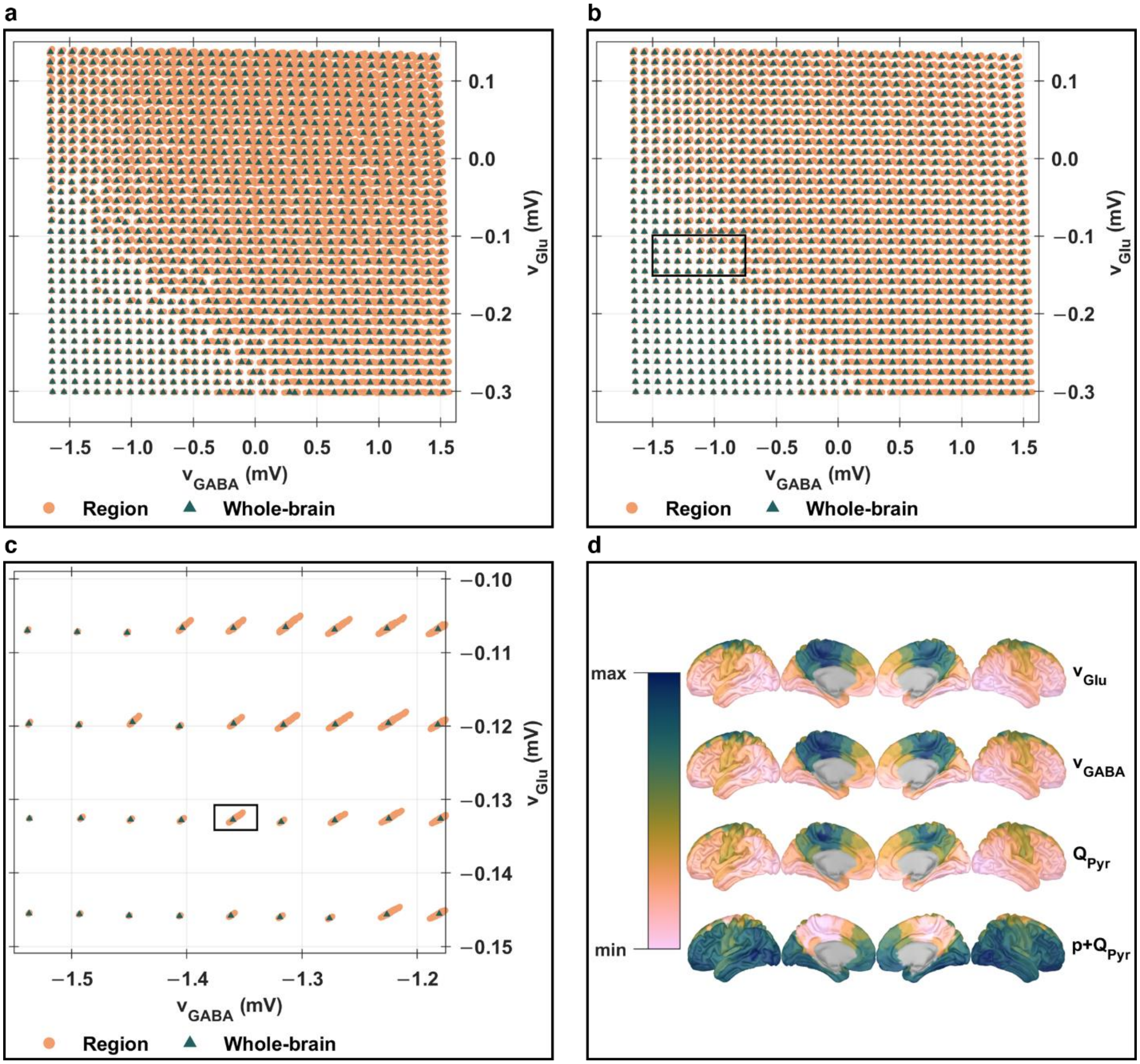

Fig D. **Simulated mean states. (a)** Displays simulated mean states at both whole-brain and regional levels within the (v_{Glu}; v_{GABA}) plane, as estimated from the calibrated simulations. The green dots indicate whole-brain values, totaling 1225, while the orange dots represent regional values, with 216 orange dots for each green dot. **(b)** Similar to panel **(a)**, but here the simulated mean states at the whole-brain and regional levels are derived from the ten simulation

batches, rather than the calibrated simulations. A black rectangular outline in this panel is further explored in panel **(c)**. **(d)** Shows the simulated mean states at the regional level corresponding to the simulation outlined in panel **(c)**. Details on the brain parcellation are available in *S3 File*.

As previously stated, in our analysis, we found no meaningful benefit in conducting additional simulations with lower ω_{GABA} values. This conclusion is illustrated in Fig D(b–c), which displays the spread of regional states around the whole-brain state. This spread highlights the inherent heterogeneity of the simulated networks. Notably, the simulations conducted at the lowest ω_{GABA} values, corresponding to areas on the parameter plane with low v_{Glu} and v_{GABA}, exhibited the least regional variability and displayed noisy spatial structures. In contrast, simulations like the one depicted in Fig D(d), with higher ω_{GABA}, highlight a different scenario where the network model demonstrates evident heterogeneity and features non-random spatial structures.

Moreover, Fig D(a–c), in conjunction with Fig B, reveal that a line equidistant from the Hopf bifurcation locus effectively approximates the onset of interesting dynamical network states. This approximation becomes evident where regional and whole-brain values begin to diverge markedly, indicating a network-level bifurcation-like phenomenon. As elaborated in *S5 File*, this abrupt shift in regional variability across the simulation parameter plane is primarily attributed to interactions driven by white noise within the network model. These interactions engage the heterogeneously specified stochastic components q, the structural layers Ω_{Pyr} and Ω_{Ast}, and the periodic orbit bifurcation landscapes of the neuronal compartments, leading to diverse responses across different nodes. The complexity of these interactions suggests that the behavior of a single node or a homogeneously parametrized network cannot trivially or fully explain these dynamics.

It is important to note that while the mean states depicted in Fig D(a–c), based on whole-brain values, correspond uniquely to pairs $(\omega_{\mathrm{Glu}}; \omega_{\mathrm{GABA}})$, there are overlaps between states due to the variance in the stochastic white noise components q, where the standard deviation was set at 10 Hz. Moreover, Fig D(b–c) reveal that interpolation artifacts may occur, particularly near areas of the parameter plane where simulations exhibit markedly divergent states. Additionally, it is unnecessary to simulate v_{Glu} values higher than 0.15 mV in our study, as these are far from the Hopf bifurcation locus and result in network dynamics and states that are highly redundant across the rest of the parameter plane.

Finally, observations from Fig D(d) show interesting spatial correlations in the simulation data: there is a positive correlation across regions between v_{Glu}, v_{GABA}, and Q_{Pyr}, and a negative correlation between v_{Glu} and the combined quantity $p + Q_{\mathrm{Pyr}}$. These findings align with our simulation design and suggest that regions with elevated mean levels of $\mathrm{Glu_e}$ also exhibit higher mean levels of $\mathrm{GABA_e}$ and Q_{Pyr}, and conversely. Besides, the fluctuations in $\mathrm{GABA_e}$ predominantly influence the mean levels of the quantity $p + Q_{\mathrm{Pyr}} = q - v_{\mathrm{GABA}}\, a/A + Q_{\mathrm{Pyr}}$ through a monotonically decreasing relationship.

S2.3 Summary and generalization

In summary, we established a biologically relevant exploration grid for the parameters $(\omega_{\mathrm{Glu}}; \omega_{\mathrm{GABA}})$, ensuring that $\mathrm{Glu_e}$ spans the range [5; 15] µmol and $\mathrm{GABA_e}$ covers [5; 35] µmol. The interplay between ω_{Glu} and ω_{GABA} enables our network model to explore a variety of neuronal dynamic states. These states are typified by stable periodic orbits, which exhibit variable peak–peak amplitudes and alpha band frequencies, along with distinct patterns of excitatory and inhibitory activity. This variability contributes to a spectrum of functional network architectures.

It is important to note that the key parameterization criteria such as the LFP frequency band, the concentration boundaries for $\mathrm{Glu_e}$ and $\mathrm{GABA_e}$, and the initial network state are all flexible and adjustable. For example, changes to the LFP frequency band can be achieved by scaling the parameters A, a, B, and b, which determine the neuronal postsynaptic potential impulse response functions, while preserving the ratios A/a and B/b. Although such modifications do not affect singular points, as demonstrated in Equation *(A)*, they do adjust periodic orbit frequencies through scaling. However, the biological realism of these adjusted parameters remains subject to debate (Chehelcheraghi et al., 2016; David & Friston, 2003). Moreover, the specified concentration ranges for $\mathrm{Glu_e}$ and $\mathrm{GABA_e}$ can be altered by adjusting the parameters governing the sigmoidal or Michaelis–Menten functions. This would also necessitate corresponding modifications to the network model's parameters involved in uptake, release, and feedback mechanisms to maintain internal consistency.

S2.4 Simulated time series

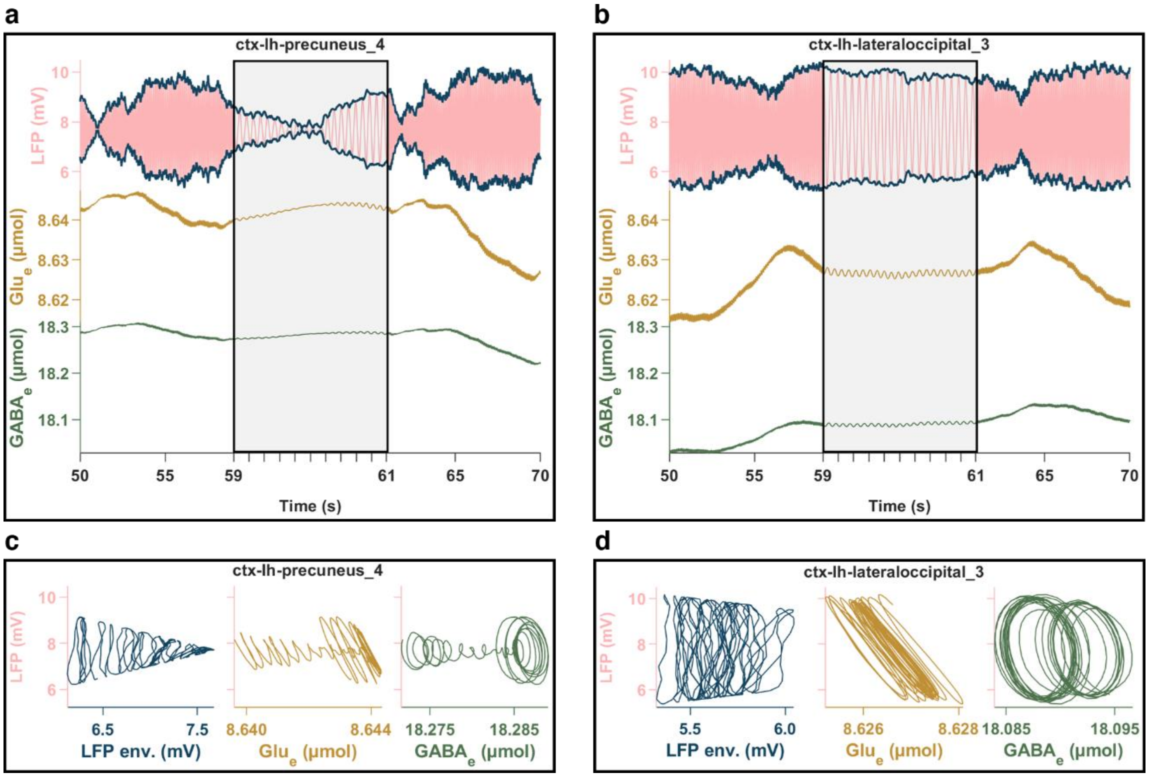

Fig E. **Physiological whole-brain network simulation, bridging electrophysiology and neurotransmission.** This figure illustrates a simulation with $\omega_{\mathrm{Glu}} = 5.64\ \mu\mathrm{mol}^{-1}$ and $\omega_{\mathrm{GABA}} = 0.52\ \mu\mathrm{mol}^{-1}$, corresponding to whole-brain levels of $\upsilon_{\mathrm{Glu}} = -0.13$ mV and $\upsilon_{\mathrm{GABA}} = -0.49$ mV. Panel **(a)** displays the neural activity within a specific region of the left precuneus cortex, while panel **(b)** focuses on a region within the left lateral occipital cortex. Details on the brain parcellation are provided in *S3 File*. The depicted time series cover the interval from 50 to 70 seconds, with a black rectangular frame highlighting a detailed view of the activity from 59 to 61 seconds. Panels **(c)** and **(d)** show phase spaces corresponding to the detailed view of activity from 59 to 61 seconds in panels **(a)** and **(b)**, respectively. In panels **(c)** and **(d)**, "LFP env." refers to the lower envelope.

Fig E showcases an illustrative simulation (selected from a total of 12250) as detailed in the *Main Manuscript*, featuring a network model composed of 216 nodes, with parameters set according to *Table B* in *S1 File*. The figure illustrates that the LFPs in this simulation exhibit amplitude modulations at frequencies substantially lower than their primary peak frequency, of

approximately 10.5 Hz. Concurrently, Glu_e and $GABA_e$ display mean quasi-stationary slow fluctuations (less than 0.5 Hz, as shown in Table A), which are positively correlated. These fluctuations reflect certain characteristics of the LFP envelopes, both upper and lower, suggesting a complex interaction between faster neuronal activities and slower astrocytic processes within the network.

Table A. **Descriptive statistics of energy distribution across frequency bands for extracellular glutamate and GABA dynamics.** The table presents the percentage of total energy contained within each frequency band, as determined by the maximal overlap discrete wavelet transform (*MATLAB*'s modwt function) applied to the Glu_e and $GABA_e$ signals across all simulations and all network nodes. In this analysis, the demeaned simulated signals were sampled at a frequency of 256 Hz and the wavelet transform was applied using *Symlet* wavelets with four vanishing moments across eight decomposition levels. The columns represent the different frequency bands, while the rows show various descriptive statistics with "*Q*" indicating quantiles. On average, more than 95% of the energies are concentrated within the 0–0.5 Hz frequency band (the approximation coefficients at the eighth level), confirming that the analyzed signals are predominantly composed of low-frequency components below 0.5 Hz. There are only minor power contributions in the 8–16 Hz band due to the low-pass filtering effect of the neurotransmitter release rate dynamics on neuronal firing rates.

	Glu_e				$GABA_e$			
	**≤0.5 Hz**	**0.5–8 Hz**	**8–16 Hz**	**≥16 Hz**	**≤0.5 Hz**	**0.5–8 Hz**	**8–16 Hz**	**≥16 Hz**
mean	97.9	0.6	1.4	0.1	98.8	0.4	0.7	0.1
Q-1%	84.5	0.1	0.1	0.0	90.9	0.0	0.0	0.0
Q-5%	88.9	0.1	0.1	0.0	94.4	0.0	0.1	0.0
Q-25%	98.7	0.2	0.2	0.0	99.0	0.1	0.1	0.0
median	99.3	0.3	0.3	0.0	99.5	0.2	0.2	0.0
Q-75%	99.6	0.7	0.6	0.1	99.8	0.6	0.4	0.0
Q-95%	99.7	2.5	7.9	0.7	99.9	1.4	4.1	0.4
Q-99%	99.8	3.5	11.2	1.0	99.9	2.1	6.7	0.6

References

Blanchard, S., Saillet, S., Ivanov, A., Benquet, P., Bénar, C.-G., Pélégrini-Issac, M., Benali, H., & Wendling, F. (2016). A New Computational Model for Neuro-Glio-Vascular Coupling: Astrocyte Activation Can Explain Cerebral Blood Flow Nonlinear Response to Interictal Events. *PLOS ONE*, *11*(2), e0147292. https://doi.org/10.1371/journal.pone.0147292

Chehelcheraghi, M., Nakatani, C., Steur, E., & van Leeuwen, C. (2016). A neural mass model of phase–amplitude coupling. *Biological Cybernetics*, *110*(2–3), 171–192. https://doi.org/10.1007/s00422-016-0687-5

Clewley, R. (2012). Hybrid Models and Biological Model Reduction with PyDSTool. *PLoS Computational Biology*, *8*(8), e1002628. https://doi.org/10.1371/journal.pcbi.1002628

David, O., & Friston, K. J. (2003). A neural mass model for MEG/EEG: coupling and neuronal dynamics. *NeuroImage*, *20*(3), 1743–1755. https://doi.org/10.1016/j.neuroimage.2003.07.015

De Pittà, M., & Berry, H. (2019). A Neuron–Glial Perspective for Computational Neuroscience. In M. De Pittà & H. Berry (Eds.), *Computational Glioscience* (pp. 3–35). Springer, Cham. https://doi.org/10.1007/978-3-030-00817-8_1

Dhooge, A., Govaerts, W., Kuznetsov, Y. A., Meijer, H. G. E., & Sautois, B. (2008). New features of the software MatCont for bifurcation analysis of dynamical systems. *Mathematical and Computer Modelling of Dynamical Systems*, *14*(2), 147–175. https://doi.org/10.1080/13873950701742754

Ferrat, L. A., Goodfellow, M., & Terry, J. R. (2018). Classifying dynamic transitions in high dimensional neural mass models: A random forest approach. *PLOS Computational Biology*, *14*(3), e1006009. https://doi.org/10.1371/journal.pcbi.1006009

Garnier, A., Vidal, A., & Benali, H. (2016). A Theoretical Study on the Role of Astrocytic Activity in Neuronal Hyperexcitability by a Novel Neuron-Glia Mass Model. *The Journal of Mathematical Neuroscience*, *6*(1), 10. https://doi.org/10.1186/s13408-016-0042-0

Griffiths, J. D., Bastiaens, S. P., & Kaboodvand, N. (2022). Whole-Brain Modelling: Past, Present, and Future. In *Advances in Experimental Medicine and Biology* (Vol. 1359, pp. 313–355). https://doi.org/10.1007/978-3-030-89439-9_13

Jansen, B. H., & Rit, V. G. (1995). Electroencephalogram and visual evoked potential generation in a mathematical model of coupled cortical columns. *Biological Cybernetics*, *73*(4), 357–366. https://doi.org/10.1007/BF00199471

Kastanenka, K. V., Moreno-Bote, R., De Pittà, M., Perea, G., Eraso-Pichot, A., Masgrau, R., Poskanzer, K. E., & Galea, E. (2020). A roadmap to integrate astrocytes into Systems Neuroscience. *Glia*, *68*(1), 5–26. https://doi.org/10.1002/glia.23632
