# Supplementary material for: Dialogue mechanisms between astrocytic and neuronal networks: A whole-brain modelling approach
Source: PLoS Comput Biol. 2025 Jan 13;21(1):e1012683. doi: 10.1371/journal.pcbi.1012683 (PMC11730384; doi:10.1371/journal.pcbi.1012683)
Supplement: S3 File — (PDF) [file pcbi.1012683.s003.pdf]

Supporting Information for "Dialogue mechanisms between astrocytic and neuronal networks: a whole-brain modelling approach"

Obaï Bin Ka'b Ali[1,2,*], Alexandre Vidal[3], Christophe Grova[4,5], Habib Benali[2,6]

1. Physics Department, Concordia University, Montreal, Canada
2. Electrical and Computer Engineering Department, Concordia University, Montreal, Canada
3. Laboratoire de Mathématiques et Modélisation d'Evry (LAMME), Université Evry, CNRS, Université Paris-Saclay, France
4. Multimodal Functional Imaging Lab, Department of Physics, Concordia School of Health, Concordia University, Montreal, Canada
5. Multimodal Functional Imaging Lab, Biomedical Engineering Department, McGill University, Montreal, Canada
6. INSERM U1146, Paris, France

* Corresponding author: ali.obaibk@gmail.com

Table of Contents

S3: Parcellation **2**

References **5**

List of Figures

Fig A. **Lausanne-2018 atlas scale three.** 2

List of Tables

Table A. **Lausanne-2018 atlas scale three.** 2

S3: Parcellation

Fig A illustrates the cortical parcellation used in this study, specifically the scale three of the Lausanne-2018 surface-based atlas with 216 parcels (Tourbier et al., 2022), version 3.0.3 available at https://github.com/connectomicslab/connectomemapper3. For ease of reference and analysis, these parcels were categorically assigned, regardless of their hemispheric location, to one of six lobes: frontal, cingulate, parietal, occipital, temporal, and insula. Table A complements Fig A by providing a detailed tabulation of how these parcels were organized.

In subsequent figures where these parcels are depicted, they will follow a consistent color-coding as shown in Fig A. The orientation convention is as follows: when parcels are displayed horizontally, those from the left hemisphere are positioned on the left and those from the right hemisphere on the right; when displayed vertically, parcels from the left hemisphere appear at the top, while those from the right hemisphere are placed at the bottom.

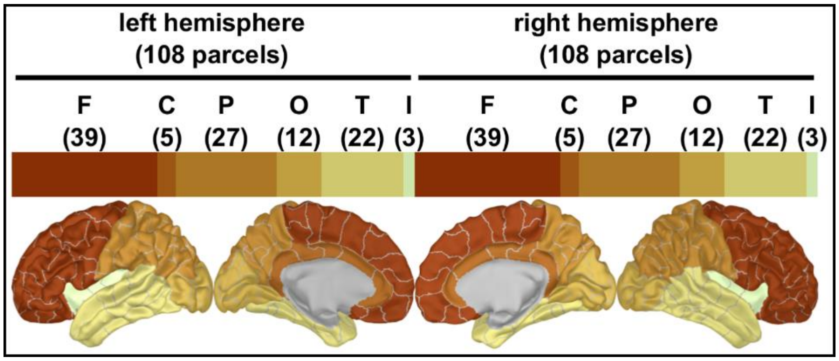

Fig A. **Lausanne-2018 atlas scale three.** Each lobe (F: frontal; C: cingulate; P: parietal; O: occipital; T: temporal; I: insula) is distinctly color-coded, ranging from dark red to yellow. The numbers in parentheses indicate the count of parcels contained within each lobe for each hemisphere.

Table A. **Lausanne-2018 atlas scale three.** Parcels in the left hemisphere are prefixed by "lh" and those in the right hemisphere by "rh". The numbers in brackets represent the sequential order of the parcels, arranged in ascending order.

**Parcel**	**Lobe**
lh.lateralorbitofrontal.[1–4]	frontal
lh.parsorbitalis.1	frontal
lh.frontalpole.1	frontal
lh.medialorbitofrontal.[1–3]	frontal
lh.parstriangularis.[1–2]	frontal
lh.parsopercularis.[1–2]	frontal
lh.rostralmiddlefrontal.[1–6]	frontal
lh.superiorfrontal.[1–8]	frontal
lh.caudalmiddlefrontal.[1–3]	frontal
lh.precentral.[1–6]	frontal
lh.paracentral.[1–3]	frontal
lh.rostralanteriorcingulate.1	cingulate
lh.caudalanteriorcingulate.1	cingulate

**Parcel**	**Lobe**
lh.posteriorcingulate.[1–2]	cingulate
lh.isthmuscingulate.1	cingulate
lh.postcentral.[1–5]	parietal
lh.supramarginal.[1–4]	parietal
lh.superiorparietal.[1–7]	parietal
lh.inferiorparietal.[1–6]	parietal
lh.precuneus.[1–5]	parietal
lh.cuneus.[1–2]	occipital
lh.pericalcarine.[1–2]	occipital
lh.lateraloccipital.[1–5]	occipital
lh.lingual.[1–3]	occipital
lh.fusiform.[1–4]	temporal
lh.parahippocampal.1	temporal
lh.entorhinal.1	temporal
lh.temporalpole.1	temporal
lh.inferiortemporal.[1–4]	temporal
lh.middletemporal.[1–4]	temporal
lh.bankssts.1	temporal
lh.superiortemporal.[1–5]	temporal
lh.transversetemporal.1	temporal
lh.insula.[1–3]	insula
rh.lateralorbitofrontal.[1–4]	frontal
rh.parsorbitalis.1	frontal
rh.frontalpole.1	frontal
rh.medialorbitofrontal.[1–3]	frontal
rh.parstriangularis.[1–2]	frontal
rh.parsopercularis.[1–2]	frontal
rh.rostralmiddlefrontal.[1–6]	frontal
rh.superiorfrontal.[1–8]	frontal
rh.caudalmiddlefrontal.[1–3]	frontal
rh.precentral.[1–6]	frontal
rh.paracentral.[1–3]	frontal
rh.rostralanteriorcingulate.1	cingulate
rh.caudalanteriorcingulate.1	cingulate
rh.posteriorcingulate.[1–2]	cingulate
rh.isthmuscingulate.1	cingulate

**Parcel**	**Lobe**
rh.postcentral.[1–5]	parietal
rh.supramarginal.[1–4]	parietal
rh.superiorparietal.[1–7]	parietal
rh.inferiorparietal.[1–6]	parietal
rh.precuneus.[1–5]	parietal
rh.cuneus.[1–2]	occipital
rh.pericalcarine.[1–2]	occipital
rh.lateraloccipital.[1–5]	occipital
rh.lingual.[1–3]	occipital
rh.fusiform.[1–4]	temporal
rh.parahippocampal.1	temporal
rh.entorhinal.1	temporal
rh.temporalpole.1	temporal
rh.inferiortemporal.[1–4]	temporal
rh.middletemporal.[1–4]	temporal
rh.bankssts.1	temporal
rh.superiortemporal.[1–5]	temporal
rh.transversetemporal.1	temporal
rh.insula.[1–3]	insula

References

Tourbier, S., Rue-Queralt, J., Glomb, K., Aleman-Gomez, Y., Mullier, E., Griffa, A., Schöttner, M., Wirsich, J., Tuncel, M. A., Jancovic, J., Cuadra, M. B., & Hagmann, P. (2022). Connectome Mapper 3: A Flexible and Open-Source Pipeline Software for Multiscale Multimodal Human Connectome Mapping. *Journal of Open Source Software*, *7*(74), 4248. https://doi.org/10.21105/joss.04248
