# Supplementary material for: Dialogue mechanisms between astrocytic and neuronal networks: A whole-brain modelling approach
Source: PLoS Comput Biol. 2025 Jan 13;21(1):e1012683. doi: 10.1371/journal.pcbi.1012683 (PMC11730384; doi:10.1371/journal.pcbi.1012683)
Supplement: S4 File — (PDF) [file pcbi.1012683.s004.pdf]

Supporting Information for “Dialogue mechanisms between astrocytic and neuronal networks: a whole-brain modelling approach”

Obaï Bin Ka’b Ali[1,2,*], Alexandre Vidal[3], Christophe Grova[4,5], Habib Benali[2,6]

1. Physics Department, Concordia University, Montreal, Canada
2. Electrical and Computer Engineering Department, Concordia University, Montreal, Canada
3. Laboratoire de Mathématiques et Modélisation d’Evry (LAMME), Université Evry, CNRS, Université Paris-Saclay, France
4. Multimodal Functional Imaging Lab, Department of Physics, Concordia School of Health, Concordia University, Montreal, Canada
5. Multimodal Functional Imaging Lab, Biomedical Engineering Department, McGill University, Montreal, Canada
6. INSERM U1146, Paris, France

* Corresponding author: ali.obaibk@gmail.com

Table of Contents

S4: Structural layers **2**

S4.1 Neuronal connectome reconstruction pipeline 2
S4.2 Astrocytic connectome reconstruction pipeline 3
S4.3 Analyses 4

Reference **5**

List of Figures

Fig A. **Basic topological properties of the structural layers.** 4

S4: Structural layers

S4.1 Neuronal connectome reconstruction pipeline

Diffusion and structural 3 T MRI data for ten subjects (101107, 105923, 108323, 111514, 116726, 140117, 146129, 156334, 158136, and 257845) were obtained from the *Human Connectome Project* (*Young Adult*) dataset, as detailed in (Van Essen et al., 2013). The diffusion data were acquired using a 2D spin-echo echo-planar imaging sequence at a high spatial resolution of 1.25 mm isotropic. Diffusion sensitization was applied with three *b*-values (1000, 2000, and 3000 s/mm^2) across 90 directions per *b*-shell, alongside 18 non-diffusion-weighted (b = 0 s/mm^2) images. T1-weighted images were acquired using a 3D magnetization-prepared rapid gradient-echo sequence at a resolution of 0.7 mm resolution. We used minimally processed diffusion-weighted images (DWI) that were corrected for susceptibility-induced distortions, eddy currents, and subject motion using a non-parametric framework based on Gaussian processes (Glasser et al., 2013). The structural data, also minimally processed, included *FreeSurfer* derivatives and T1-weighted images sampled at the same resolution as the diffusion data (Glasser et al., 2013).

For volumetric-based Lausanne-2018 parcellations for each subject (Tourbier et al., 2022), as depicted in *S3 File*, we employed the *fsaverage-FreeSurfer* template (Fischl, 2012), version 6.0.0 available at https://github.com/freesurfer/freesurfer. This was used as an intermediate reference for surface-to-surface (*mri_surf2surf*) and subsequently surface-to-volume (*mri_aparc2aseg*) transformations.

Our tractography-based connectome reconstruction pipeline was designed to address streamline termination and quantification biases inherent in tractography (Yeh et al., 2021). The pipeline comprises six main steps.

(i) The initial step involved running *Tractoflow* (Theaud et al., 2020), version 2.2.1 available at https://github.com/scilus/tractoflow. This fully automatic diffusion MRI tractography pipeline was used to estimate brain maps for each subject, including fractional anisotropy, fiber orientation distribution function, and segmented T1 tissues. Configurations for *Tractoflow* used default parameters unless specified otherwise and were set to include the following steps: DWI brain extraction, DWI denoising, DWI N4 bias correction, DTI metrics derived from *b*-shells at 0 and 1000 s/mm^2, fODF metrics calculated from *b*-shells at 1000, 2000, and 3000 s/mm^2 with manually fixed fiber response function in mm^2/s at (15; 4; 4) $\times 10^{-4}$, T1 brain extraction, T1 N4 bias correction, and T1 tissue segmentation.

(ii) Following *Tractoflow*, the generated maps were input into *SET* (St-Onge et al., 2018), version 1.0 available at https://set-documentation.readthedocs.io. *SET (surface-enhanced tractography)* mitigates streamline termination biases by integrating cortical surface geometry priors into tractography. It imposes surface flow constraints on reconstructed streamlines, ensuring intersection with the brain's white surface to enhance cortical coverage and reduce gyral bias. This approach produced surface-based tractograms for each subject. The *freesurfer_basic* pipeline profile was used, one million random seeds were initiated for probabilistic tracking, and streamlines outside the 10–300 mm length range were excluded from the tractograms. All other default settings were retained, except for deactivating the warping from T1 to diffusion space.

(iii) The tractograms derived from *SET* were then analyzed using the *Scilpy* Python library, version 1.3.0 available at https://github.com/scilus/scilpy. The tractograms were

decomposed on a parcel-to-parcel basis (*scil_decompose_connectivity.py*), constrained by the scale three of the Lausanne-2018 parcellation atlas. This step included filtering criteria such as streamline length, curvature, and winding angle to eliminate invalid streamlines (*scil_remove_invalid_streamlines.py*; *scil_detect_streamlines_loops.py*).

(iv) To further refine the tractograms and assign a quantitative weight to each streamline in a biologically plausible manner, we applied *COMMIT-2* (Schiavi et al., 2020), accessible at https://github.com/daducci/COMMIT and through https://github.com/scilus/scilpy. *COMMIT-2 (a variant of convex optimization modeling for microstructure informed tractography)* employs a linear forward model of tissue microstructure, optimizing the anatomical assumption that axons are organized into bundles. A penalty coefficient was set at 0.005 for all subjects to target an average connectome density of approximately 25 percent across the cohort. The microstructural model used included a *stick* model to represent the anisotropic contributions of the axonal streamlines and a *ball* model to address potential CSF contaminations, applying default diffusivities (*scil_run_commit.py --ball_stick*).

(v) From the *COMMIT-2* optimized tractograms, connectomes were derived for each subject by defining weights between any two parcels as the sum of the *COMMIT-2* weights for each connecting streamline (*scil_compute_connectivity.py*). These connectomes are quantitative, with each weight representing a measure of connectivity strength that reflects anatomical and microstructural characteristics.

(vi) Finally, the parameter matrix Ω_{Pyr} was computed as the mean of these connectomes across subjects. Weights less than or equal to 10^{-6} were considered negligible. Prior to initiating simulations, Ω_{Pyr} was normalized to ensure that the sum of coefficients equaled one for each row.

It is important to note that the matrix Ω_{Pyr} is symmetric by construction since diffusion-MRI-based tractograms are inherently undirected. However, long-range projections between neuronal populations are predominantly directed. Promising approaches using axonal tract-tracing datasets to infer directionality, as well as to correct for frequently misreconstructed connections between homologous brain regions due to crossing fibers in diffusion MRI, are currently being explored and are planned to be integrated into future studies (Bezgin et al., 2017; Shen et al., 2019). Such adjustments are vital for improving the anatomical accuracy and biological plausibility of structural connectomes, significantly enhancing the congruence between structural and functional connectivity (Suárez et al., 2020).

S4.2 Astrocytic connectome reconstruction pipeline

To reconstruct cortical surfaces for the *ICBM-2009c-asymmetric* template (Fonov et al., 2011), we employed *FreeSurfer*. The reconstruction process (*recon-all -all*) involved both T1-weighted and T2-weighted images. A mid-surface was generated by expanding the native white surfaces outward to 50 percent of the cortical thickness (*mris_expand -thickness*). Utilizing this derived mid-surface, which comprised approximately 160,000 vertices per hemisphere, we calculated the coefficients of the parameter matrix Ω_{Ast}. Coefficients between any two adjacent parcels were determined based on the shortest path distances, weighted by Euclidean lengths. Parcels were defined by integrating the scale three of the Lausanne-2018 atlas into the *ICBM-2009c-asymmetric* template through surface-to-surface mappings (*mri_surf2surf*), using the *fsaverage-FreeSurfer* template as an intermediary reference. Prior to initiating simulations, Ω_{Ast} was normalized to ensure that the sum of coefficients equaled one for each row.

The matrix Ω_{Ast} simulates the attenuation of intercellular signaling across distances via gap junctions among astrocytic populations (Lallouette et al., 2019; Vasile et al., 2017), using geodesic distances as a coarse approximation for modeling the strength of astrocytic coupling based on physical proximity. Looking forward, future studies could extend this framework to include vascular networks, which offer a promising avenue to elucidate the interaction pathways among astrocytic populations as well as between astrocytic and neuronal populations, an approach especially relevant given the resolution limits of current neuroimaging techniques (Hösli et al., 2022; Kugler et al., 2021).

S4.3 Analyses

In our network model, we tasked the structural layers, Ω_{Pyr} and Ω_{Ast}, with defining all spatial patterns, necessitating an analysis of their basic topological properties. Fig A illustrates the community organization, participation coefficient, and degree distributions within these layers. We analyzed these layers, prior to their normalization, using the default routines provided by the *Brain Connectivity Toolbox*, 2019-03-03 release available at https://www.nitrc.org/projects/bct. Community detection was performed using a spectral optimization algorithm (*modularity_und.m*), and the participation coefficients (*participation_coef.m*) were calculated from the identified communities.

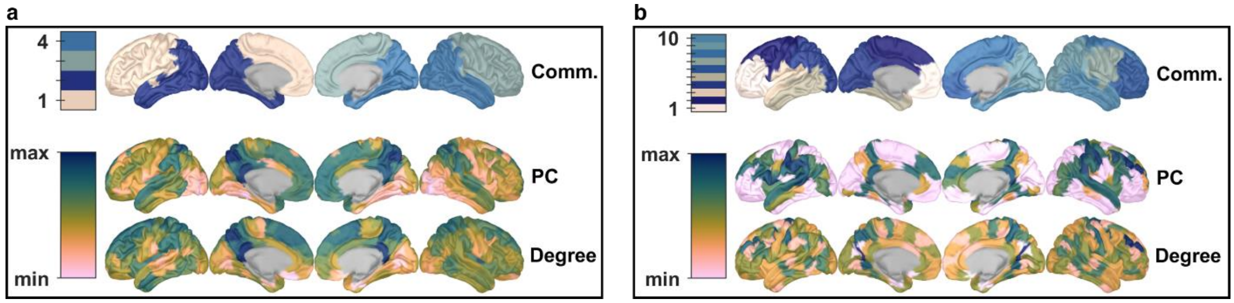

Fig A. **Basic topological properties of the structural layers.** Panel **(a)**: Ω_{Pyr}. Panel **(b)**: Ω_{Ast}. Panels **(a)–(b)**: Comm. (community structures); PC (participation coefficient).

Fig A(a), focusing on Ω_{Pyr}, highlights that the frontal–cingulate–insula and parietal–occipital–temporal regions form two distinct communities. Notably, the precuneus regions exhibit substantial connectivity with the broader brain network, as evidenced by high participation coefficients and degrees, in contrast to the occipital regions, which display lower participation and fewer connections. Fig A(b), focusing on Ω_{Ast}, indicates that the lobar domains of the brain approximately mirror the geometry of the brain's cortical surface. It is interesting to observe that while the communities in Ω_{Pyr} largely recur in Ω_{Ast}, they are divided into smaller modules.

A comprehensive graph-theoretical analysis of these layers was beyond the scope of this study, and as such, we have refrained from presenting extended details. However, it is essential to acknowledge that our findings, particularly regarding Ω_{Ast} (but less so for Ω_{Pyr}), depend heavily on multiple factors such as parcellation and community detection methods. This introduces potential biases, underscored by the critical impact of the choice of parcellation scheme on neuroimaging studies (Arslan et al., 2018; Messé, 2020). For instance, our use of a surface-based anatomical atlas with 216 regions to define network nodes could yield different results if alternative atlases (whether functional, multimodal, volumetric, or varying in spatial resolutions) were employed.

Reference

Arslan, S., Ktena, S. I., Makropoulos, A., Robinson, E. C., Rueckert, D., & Parisot, S. (2018). Human brain mapping: A systematic comparison of parcellation methods for the human cerebral cortex. *NeuroImage*, *170*(April 2017), 5–30. https://doi.org/10.1016/j.neuroimage.2017.04.014

Bezgin, G., Solodkin, A., Bakker, R., Ritter, P., & McIntosh, A. R. (2017). Mapping complementary features of cross-species structural connectivity to construct realistic "Virtual Brains." *Human Brain Mapping*, *38*(4), 2080–2093. https://doi.org/10.1002/hbm.23506

Breakspear, M. (2017). Dynamic models of large-scale brain activity. *Nature Neuroscience*, *20*(3), 340–352. https://doi.org/10.1038/nn.4497

De Pittà, M., & Berry, H. (2019). A Neuron–Glial Perspective for Computational Neuroscience. In M. De Pittà & H. Berry (Eds.), *Computational Glioscience* (pp. 3–35). Springer, Cham. https://doi.org/10.1007/978-3-030-00817-8_1

Fields, R. D., Woo, D. H., & Basser, P. J. (2015). Glial Regulation of the Neuronal Connectome through Local and Long-Distant Communication. *Neuron*, *86*(2), 374–386. https://doi.org/10.1016/j.neuron.2015.01.014

Fischl, B. (2012). FreeSurfer. *NeuroImage*, *62*(2), 774–781. https://doi.org/10.1016/j.neuroimage.2012.01.021

Fonov, V., Evans, A. C., Botteron, K., Almli, C. R., McKinstry, R. C., & Collins, D. L. (2011). Unbiased average age-appropriate atlases for pediatric studies. *NeuroImage*, *54*(1), 313–327. https://doi.org/10.1016/j.neuroimage.2010.07.033

Glasser, M. F., Sotiropoulos, S. N., Wilson, J. A., Coalson, T. S., Fischl, B., Andersson, J. L., Xu, J., Jbabdi, S., Webster, M., Polimeni, J. R., Van Essen, D. C., & Jenkinson, M. (2013). The minimal preprocessing pipelines for the Human Connectome Project. *NeuroImage*, *80*, 105–124. https://doi.org/10.1016/j.neuroimage.2013.04.127

Griffiths, J. D., Bastiaens, S. P., & Kaboodvand, N. (2022). Whole-Brain Modelling: Past, Present, and Future. In *Advances in Experimental Medicine and Biology* (Vol. 1359, pp. 313–355). https://doi.org/10.1007/978-3-030-89439-9_13

Hösli, L., Zuend, M., Bredell, G., Zanker, H. S., Porto de Oliveira, C. E., Saab, A. S., & Weber, B. (2022). Direct vascular contact is a hallmark of cerebral astrocytes. *Cell Reports*, *39*(1), 110599. https://doi.org/10.1016/j.celrep.2022.110599

Kugler, E. C., Greenwood, J., & MacDonald, R. B. (2021). The "Neuro-Glial-Vascular" Unit: The Role of Glia in Neurovascular Unit Formation and Dysfunction. *Frontiers in Cell and Developmental Biology*, *9*. https://doi.org/10.3389/fcell.2021.732820

Lallouette, J., De Pittà, M., & Berry, H. (2019). Astrocyte Networks and Intercellular Calcium Propagation. In M. De Pittà & H. Berry (Eds.), *Computational Glioscience* (pp. 177–210). Springer International Publishing. https://doi.org/10.1007/978-3-030-00817-8_7

Magistretti, P. J., & Allaman, I. (2015). A Cellular Perspective on Brain Energy Metabolism and Functional Imaging. *Neuron*, *86*(4), 883–901. https://doi.org/10.1016/j.neuron.2015.03.035

Messé, A. (2020). Parcellation influence on the connectivity-based structure–function relationship in the human brain. *Human Brain Mapping*, *41*(5), 1167–1180. https://doi.org/10.1002/hbm.24866

Schiavi, S., Ocampo-Pineda, M., Barakovic, M., Petit, L., Descoteaux, M., Thiran, J.-P., & Daducci, A. (2020). A new method for accurate in vivo mapping of human brain connections using microstructural and anatomical information. *Science Advances*, *6*(31), eaba8245. https://doi.org/10.1126/sciadv.aba8245

Shen, K., Bezgin, G., Schirner, M., Ritter, P., Everling, S., & McIntosh, A. R. (2019). A macaque connectome for large-scale network simulations in TheVirtualBrain. *Scientific Data*, *6*(1), 123. https://doi.org/10.1038/s41597-019-0129-z

St-Onge, E., Daducci, A., Girard, G., & Descoteaux, M. (2018). Surface-enhanced tractography (SET). *NeuroImage*, *169*(December 2017), 524–539. https://doi.org/10.1016/j.neuroimage.2017.12.036

Suárez, L. E., Markello, R. D., Betzel, R. F., & Misic, B. (2020). Linking Structure and Function in Macroscale Brain Networks. *Trends in Cognitive Sciences*, *24*(4), 302–315. https://doi.org/10.1016/j.tics.2020.01.008

Theaud, G., Houde, J.-C., Boré, A., Rheault, F., Morency, F., & Descoteaux, M. (2020). TractoFlow: A robust, efficient and reproducible diffusion MRI pipeline leveraging Nextflow & Singularity. *NeuroImage*, *218*(April), 116889. https://doi.org/10.1016/j.neuroimage.2020.116889

Tourbier, S., Rue-Queralt, J., Glomb, K., Aleman-Gomez, Y., Mullier, E., Griffa, A., Schöttner, M., Wirsich, J., Tuncel, M. A., Jancovic, J., Cuadra, M. B., & Hagmann, P. (2022). Connectome Mapper 3: A Flexible and Open-Source Pipeline Software for Multiscale Multimodal Human Connectome Mapping. *Journal of Open Source Software*, *7*(74), 4248. https://doi.org/10.21105/joss.04248

Van Essen, D. C., Smith, S. M., Barch, D. M., Behrens, T. E. J., Yacoub, E., & Ugurbil, K. (2013). The WU-Minn Human Connectome Project: An overview. *NeuroImage*, *80*, 62–79. https://doi.org/10.1016/j.neuroimage.2013.05.041

Vasile, F., Dossi, E., & Rouach, N. (2017). Human astrocytes: structure and functions in the healthy brain. *Brain Structure and Function*, *222*(5), 2017–2029. https://doi.org/10.1007/s00429-017-1383-5

Yeh, C., Jones, D. K., Liang, X., Descoteaux, M., & Connelly, A. (2021). Mapping Structural Connectivity Using Diffusion <scp>MRI</scp> : Challenges and Opportunities. *Journal of Magnetic Resonance Imaging*, *53*(6), 1666–1682. https://doi.org/10.1002/jmri.27188
