# Supplementary material for: Dialogue mechanisms between astrocytic and neuronal networks: A whole-brain modelling approach
Source: PLoS Comput Biol. 2025 Jan 13;21(1):e1012683. doi: 10.1371/journal.pcbi.1012683 (PMC11730384; doi:10.1371/journal.pcbi.1012683)
Supplement: S5 File — (PDF) [file pcbi.1012683.s005.pdf]

Supporting Information for “Dialogue mechanisms between astrocytic and neuronal networks: a whole-brain modelling approach”

Obaï Bin Ka’b Ali[1,2,*], Alexandre Vidal[3], Christophe Grova[4,5], Habib Benali[2,6]

1. Physics Department, Concordia University, Montreal, Canada
2. Electrical and Computer Engineering Department, Concordia University, Montreal, Canada
3. Laboratoire de Mathématiques et Modélisation d’Evry (LAMME), Université Evry, CNRS, Université Paris-Saclay, France
4. Multimodal Functional Imaging Lab, Department of Physics, Concordia School of Health, Concordia University, Montreal, Canada
5. Multimodal Functional Imaging Lab, Biomedical Engineering Department, McGill University, Montreal, Canada
6. INSERM U1146, Paris, France

* Corresponding author: ali.obaibk@gmail.com

Table of Contents

S5: Neuron-astrocyte network activity analysis 2

S5.1 Interconnections between neuronal post-synaptic potential dynamics and periodic orbit features of bifurcation diagrams 2
S5.2 Links between neurotransmission and electrophysiological amplitude modulations 2
S5.3 Characterisation of global heterogeneity among networks nodes 3
S5.4 Applying clustering analysis to explore spatial relationships between neurotransmission and amplitude modulations of bioelectrical activity 5

List of Figures

Fig A. **Analysis of post-synaptic potential dynamics through bifurcation diagram features.** 2
Fig B. **Correlations between neurotransmission and amplitude modulations of bioelectrical activity.** 3
Fig C. **Analysis of global network heterogeneity.** 4
Fig D. **Clustering analysis of whole-brain network activity patterns.** 5
Fig E. **Input data for clustering analysis based on Gaussian mixture models.** 6
Fig F. **Means of Gaussian mixture model.** 6
Fig G. **Correlation matrix of Gaussian mixture model.** 7

S5: Neuron-astrocyte network activity analysis

S5.1 Interconnections between neuronal post-synaptic potential dynamics and periodic orbit features of bifurcation diagrams

In Fig A(a), which is identical to *Fig 3b* in the *Main Manuscript*, the distributions of LFP peak–peak amplitude and peak frequency, especially their local extrema, are shown to closely align with the contour lines of periodic orbit LFP peak–peak amplitudes. In Fig A(b), a refined analysis on a narrower section of the heatmaps highlights, in particular, the abrupt shifts in LFP peak–peak amplitude values, indicating sharp changes in the surface gradient flows across the first, second, and third isolines. Fig A(c) offers a simplified view of some local extrema for LFP peak–peak amplitude and peak frequency by illustrating the changes in the sign of scalar products between the two surface gradients and two specific vectors. The vectors represent an assumed general flow direction, and local extrema are pinpointed, along isolines, at the points of sign change.

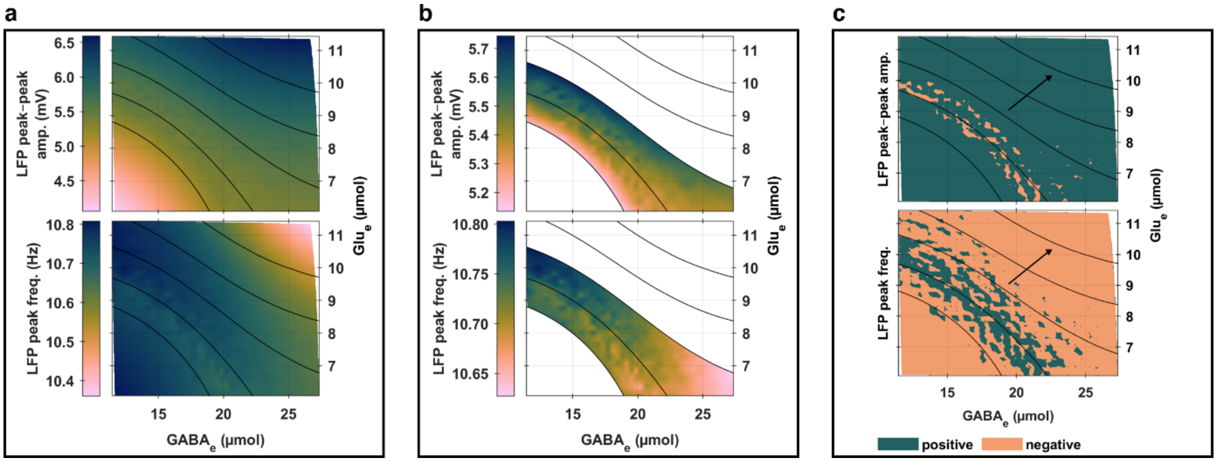

Fig A. **Analysis of post-synaptic potential dynamics through bifurcation diagram features. (a)–(b)** Whole-brain levels of LFP peak–peak amplitudes (top tile) and peak frequencies (bottom tile) as functions of whole-brain levels of Glu_e (vertical axis) and $GABA_e$ (horizontal axis). **(c)** Sign of scalar products between surface gradients (obtained from panel **(a)**) and the vectors drawn in black. **(a)–(c)** The black solid curves represent contour lines of periodic orbit LFP peak–peak amplitudes. These isolines pass through specific (Glu_e; $GABA_e$) coordinates (in μmol × μmol): (8; 15), (9; 15), (10; 15), (11; 15), and (11; 20).

It is pertinent to note that the LFP peak frequency patterns depicted in Fig A are not fully accounted for by the periodic orbit LFP peak–peak amplitude isolines. This discrepancy arises partly because the amplitude and frequency properties of periodic orbits do not consistently exhibit a perfect linear correlation, and the LFP dynamics are nonlinearly interlinked with other aspects of the network model's dynamics. In exploring this further, we determined that different contour lines from other state variables (such as E_{Pyr} or E_{InIn}) and dynamic properties (like periodic orbit frequencies, or when applicable, mean amplitudes of periodic orbits) better explained the patterns observed across certain sections of the parameter plane.

S5.2 Links between neurotransmission and electrophysiological amplitude modulations

Fig B summarizes the whole-brain spatial Pearson-correlation patterns between the temporal fluctuations of the LFP envelope, Glu_e, and $GABA_e$, derived as the median of regional values for each simulation independently. The panels (a)–(c) illustrate the correlation patterns between LFP

envelope and Glu_e (a), LFP envelope and $GABA_e$ (b), and Glu_e and $GABA_e$ (c), respectively. Across these analyses, marked shifts in correlation values were observed along critical isolines, where notable changes in other network characteristics occur. Outside these critical zones, the correlation patterns appeared smoother and consistently mirrored the variations in astrocytic network coupling strengths. It is important to note that the correlation values between LFP envelope fluctuations and both Glu_e and $GABA_e$ were generally in the low-to-medium range. This observation persisted even when employing Spearman-correlations. These findings reinforce the discussions in the *Main Manuscript*, emphasizing that the dynamic links between neurotransmission and the amplitude modulations of bioelectrical neuronal activity are inherently nonlinear. These dynamics are further influenced by additional network dynamics and stochastic factors, shaping the observed correlation patterns.

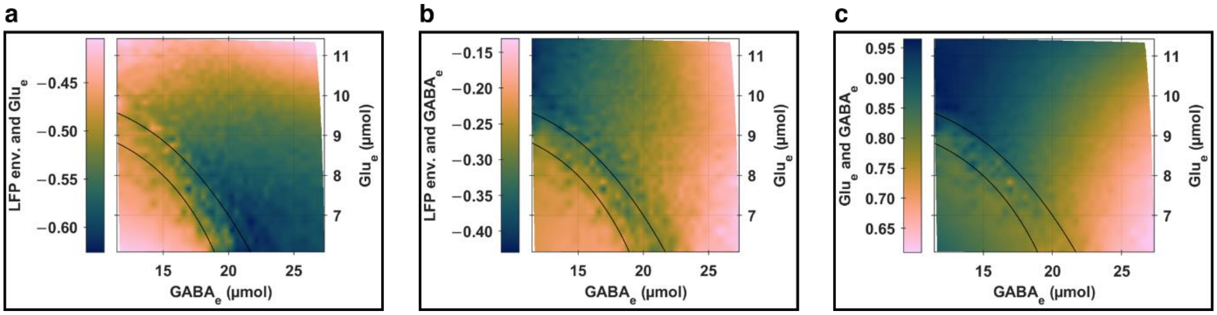

Fig B. **Correlations between neurotransmission and amplitude modulations of bioelectrical activity.** Whole-brain Pearson-correlation coefficients between **(a)** LFP envelope and Glu_e temporal fluctuations, **(b)** LFP envelope and $GABA_e$ temporal fluctuations, and **(c)** Glu_e and $GABA_e$ temporal fluctuations, as functions of whole-brain levels of Glu_e (vertical axis) and $GABA_e$ (horizontal axis). In all panels, darker colors indicate stronger absolute values of correlation coefficients. The black solid curves represent contour lines of periodic orbit LFP peak–peak amplitudes, consistent with those in *Fig 6a* of the *Main Manuscript*. This alignment facilitates comparisons with clustering analysis results of network activity patterns. Each isoline passes through specific $(Glu_e; GABA_e)$ coordinates in (µmol) × (µmol): (8; 15), or (7; 20).

S5.3 Characterisation of global heterogeneity among networks nodes

We confirmed that the patterns observed in Fig A and Fig B, or *Fig 3b* and *Fig 5* of the *Main Manuscript*, especially the abrupt transitions, were primarily a consequence of the heterogeneous white-noise-driven dynamics within our network model which promote chimera states and metastable synchrony. This is particularly true under the structural constraints of the neuronal layer Ω_{Pyr}, as evidenced by an index reflecting global heterogeneity among network nodes. For example, this index could be defined using statistical estimates of the self-coupling gains of pyramidal neurons' firing, calculated a posteriori from the simulated data. The hypothesis suggests that in a heterogeneous stochastic network, distinct nodes are unlikely to mirror each other's state unless they share identical parameters and initial conditions, particularly when the global coupling parameter ω_{Pyr} remains low-to-moderate. Furthermore, for any given node n, part of its neuronal feedback input $Q_{\mathrm{Pyr}_{[n]}}$ might predict or be predicted by its output pyramidal firing rate $F_{\mathrm{Pyr}_{[n]}}$, with the residual input acting as an independent source of stochastic variation along with the baseline neuronal firing rate $q_{[n]}$. For instance, using linear statistical methods, it is possible to estimate the pyramidal self-coupling gain of the node n, a posteriori, to represent the proportion of shared information between $Q_{\mathrm{Pyr}_{[n]}}$ and $F_{\mathrm{Pyr}_{[n]}}$. This estimation facilitates understanding node n's neuronal behavior using a codimension-2 bifurcation diagram, akin to the analyses in section *S2.1* in *S2 File*, but incorporating the estimated nodal self-coupling gain to reflect stochastic

network influences. Typically, the estimated self-coupling gain will not exceed ω_{Pyr}, supporting the view that a network's state spans a continuum between independent nodes and identical nodes. Thus, a whole-brain estimate of self-coupling gains, whether via multilevel regression modeling or descriptive statistics, offers a measure of global network heterogeneity.

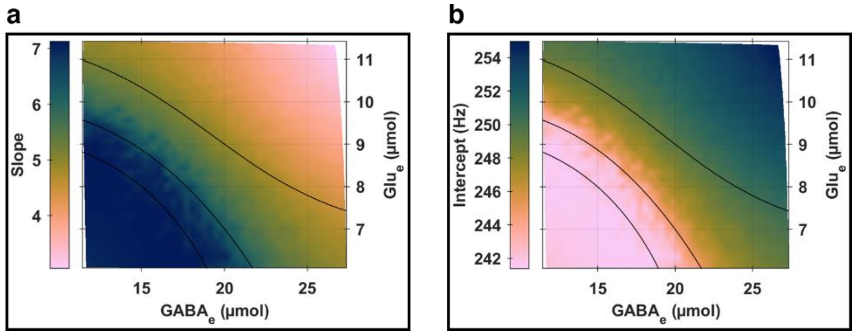

Fig C. **Analysis of global network heterogeneity. (a)** Whole-brain levels of slope estimates as functions of whole-brain levels of $\mathrm{Glu_e}$ (vertical axis) and $\mathrm{GABA_e}$ (horizontal axis). The *t*-statistics testing the null hypothesis that the slope is zero against the alternative that it is different from zero ranged from 14.5 to 98.8, affirming that all slope values were statistically significant. **(b)** Similar to panel **(a)**, but displaying intercept estimates. Here, *t*-statistics for testing the null hypothesis that the intercept is zero ranged from 451 to 1234, indicating that and all intercept values were statistically significant. **(a)–(b)** The black solid curves represent contour lines of periodic orbit LFP peak–peak amplitudes, consistent with those in *Fig 6a* of the *Main Manuscript*. This alignment facilitates comparisons with clustering analysis results of network activity patterns. Each isoline passes through specific $(\mathrm{Glu_e}; \mathrm{GABA_e})$ coordinates in (μmol) × (μmol): (8; 15), (7; 20), or (9; 20).

Fig C links global heterogeneity to whole-brain $\mathrm{Glu_e}$ and $\mathrm{GABA_e}$ levels, by presenting the outcomes of a linear regression model (using *MATLAB*'s *robustfit* function), formulated for each node n as:

$$q_{[n]} + Q_{\mathrm{Pyr}_{[n]}} = \mathrm{slope}_{[n]} F_{\mathrm{Pyr}_{[n]}} + \mathrm{intercept}_{[n]} + \mathrm{residual}_{[n]} \tag{5.1}$$

where q, Q_{Pyr}, and F_{Pyr} are from the simulated data. Fig C(a) illustrates the whole-brain average slopes (interpreted as a posteriori pyramidal self-coupling gains), while Fig C(b) shows the whole-brain average intercepts (interpreted as a posteriori q values). The slopes predominantly stay below $\omega_{\mathrm{Pyr}} = 7.5$ (see also *Table B* in *S1 File*), demonstrating a clear transition from slope ≈ 7.5 at lower neurotransmitter concentrations to a sharp decline at higher concentrations. The intercepts are consistently above $\mathrm{mean}(q) = 240$ Hz (see also *Table B* in *S1 File*) as they incorporate the effects of additive excitatory network feedback, and they exhibit a variation pattern akin to that of the slopes. Besides, slope and intercept patterns are in close alignment with the periodic orbit LFP peak–peak amplitude isolines.

We ascertained that the phenomena depicted in Fig A and Fig C did not manifest under homogeneous model parameterization, or when ω_{Glu} and ω_{GABA} spanned differing value ranges compared to the ones specified in *Table B* in *S1 File*. Our analysis traced the origins of these phenomena to the specified standard deviation of the baseline neuronal firing rates ($\mathrm{SD}(q) = 10$ Hz, as outlined in *Table B* in *S1 File*) and to the properties of the periodic orbit bifurcation landscapes shown in the *Fig 4* of the *Main Manuscript*. Indeed, single-node simulations focused solely on the neuronal compartment have proven instructive. We set the parameters ω_{Pyr} (which, as discussed in section *S2.1* in *S2 File*, acts as a nodal self-feedback parameter on pyramidal cells in the context of a network composed of a single node or a homogeneous network), $\mathrm{mean}(q)$,

v_{Glu}, and v_{GABA} using Fig C, and we adjusted the remaining parameters (A, B, a, b, $v_{\max}$, v_0^{Pyr}, v_0^{ExIn}, v_0^{InIn}, r, $\mathrm{SD}(q)$, $C^{\mathrm{Pyr}\to\mathrm{ExIn}}$, $C^{\mathrm{ExIn}\to\mathrm{Pyr}}$, $C^{\mathrm{Pyr}\to\mathrm{InIn}}$, and $C^{\mathrm{InIn}\to\mathrm{Pyr}}$) according to *Table B* in *S1 File*. These simulations produced heatmaps virtually identical to those in Fig A(a), thus replicating the whole-brain states of the heterogeneously stochastic network model.

These findings underscore the occurrence of distinct oscillatory dynamic behaviors in heterogeneous networks, behaviors that are absent in both non-stochastic and homogeneously stochastic settings. However, these dynamics are predictable through the analysis of *high-order* bifurcation diagram features, which in this context refer to complex characteristics such as periodic orbit peak–peak amplitude isolines. These features, seldom explored in conventional bifurcation analyses, provide a novel perspective on stochastic dynamical analysis, extending beyond traditional bifurcation theory. This approach also lays the groundwork for more intricate investigations, especially in network scenarios where statistically summarizing network states at a whole-brain level is inadequate.

S5.4 Applying clustering analysis to explore spatial relationships between neurotransmission and amplitude modulations of bioelectrical activity

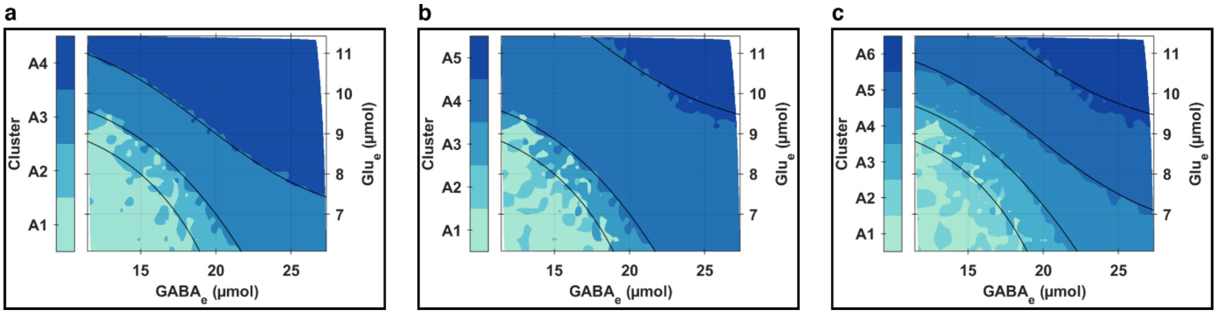

Fig D. **Clustering analysis of whole-brain network activity patterns.** This figure extends the *Fig 6a* of the *Main Manuscript* using a Gaussian mixture model with **(a)** four, **(b)** five, and **(c)** six components. The black solid curves represent contour lines of periodic orbit LFP peak–peak amplitudes, with each isoline visually selected to align with cluster frontiers.

The panel (a) of Fig D replicates *Fig 6a* of the *Main Manuscript* where clusters were initially identified using a Gaussian mixture model with four components, while the panels (b) and (c) present clusters derived from models with five and six components, respectively. All clusters, regardless of the model, largely maintain spatial contiguity. Cluster A2 from panel (a) displays consistency across the other models. However, cluster A1 from (a) experiences subtle fragmentations in the other models, and cluster A4 from (a) eventually divides into two distinct clusters in (c). These results corroborate the global network heterogeneity profile discussed in section *S5.1*, where it was determined that within cluster A1, nodes exhibited homogeneous behaviors, leading to a lack of clear spatial structures and higher noise levels. In contrast, at the frontiers between clusters A1 and A2 and beyond, nodes displayed increasingly heterogeneous behaviors.

Fig E displays the three spatial patterns used as input for clustering analysis: normalized temporal standard deviations of LFP envelopes, and normalized temporal means of $\mathrm{Glu_e}$ and $\mathrm{GABA_e}$.

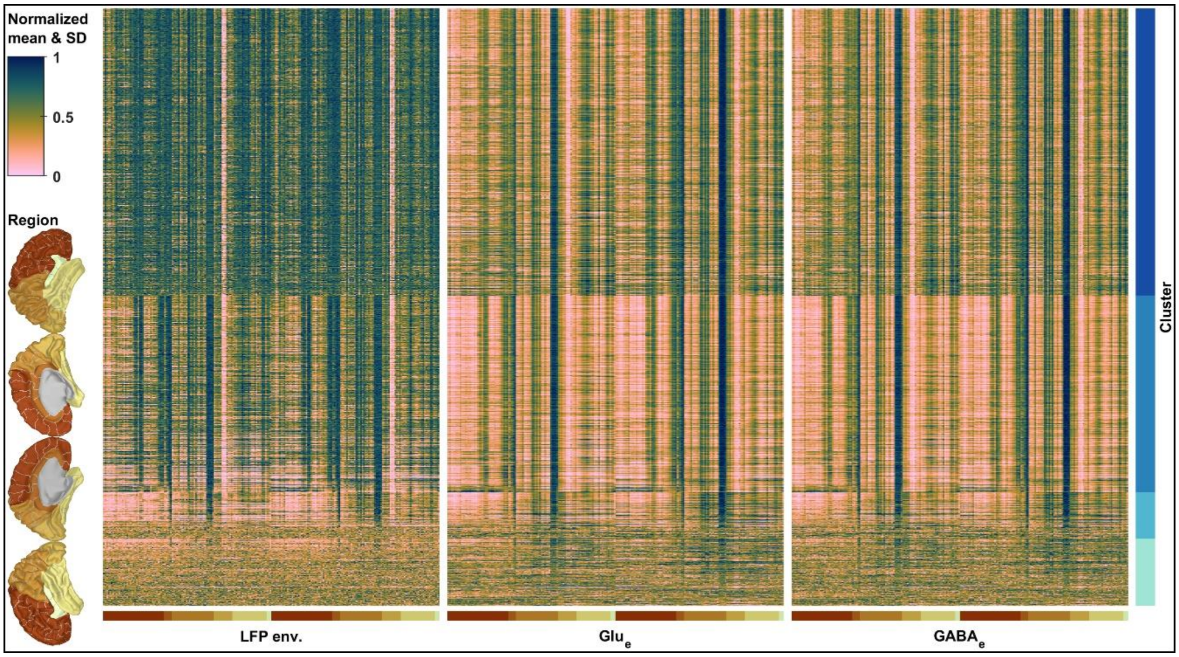

Fig E. **Input data for clustering analysis based on Gaussian mixture models.** This figure displays the spatial patterns of normalized temporal standard deviations (SD) for LFP envelopes and normalized temporal means for Glu$_e$ and GABA$_e$ across 216 brain regions, resulting in a matrix of 216×3 columns. The parcellation and regions are delineated according to the conventions specified in *S3 File*. Each row corresponds to one of the 10×1225 total simulations, organized according to their cluster assignments determined by a Gaussian mixture model with four components, as shown in *Fig 6a* of *Main Manuscript*. The color map on the right, ranging from light blue (bottom, cluster A1) to dark blue (top, cluster A4), indicates the cluster membership of each simulation.

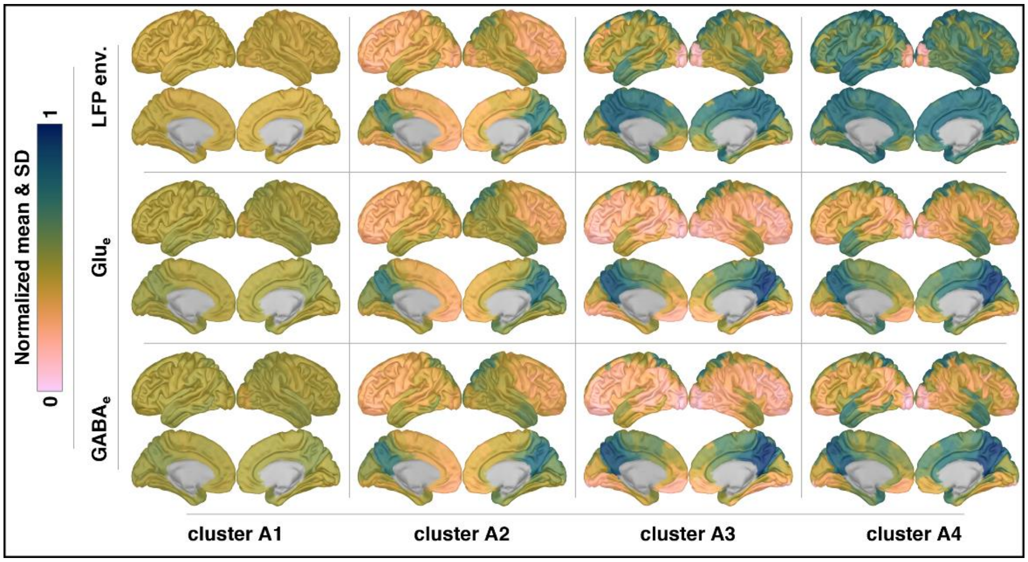

Fig F. **Means of Gaussian mixture model.** This figure presents the cluster means of normalized temporal standard deviations (SD) for LFP envelopes and normalized temporal means for Glu$_e$ and GABA$_e$, as fitted by a Gaussian mixture model with four components.

Fig F extends *Fig 7* of the *Main Manuscript*, illustrating the mean values of clusters as fitted by the Gaussian mixture model with four components. A clear correspondence is observed with the input data from Fig E, particularly highlighting regions like the precuneus cortices within the parietal lobe, which exhibit very high values across clusters A2, A3, and A4.

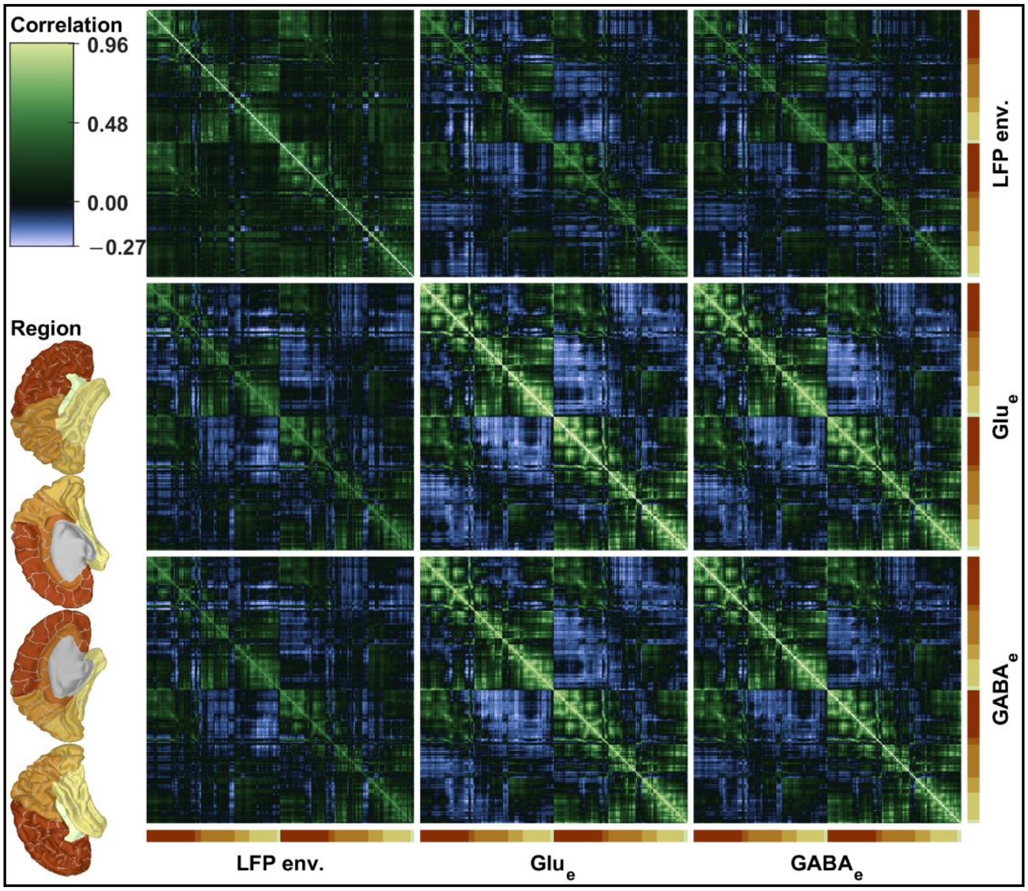

Fig G. **Correlation matrix of Gaussian mixture model.** This figure illustrates the correlation matrix for the normalized temporal standard deviations of LFP envelopes and the normalized temporal means of Glu_e and $GABA_e$, fitted by a Gaussian mixture model with four components. Diagonal values are omitted and the background appears in white. This matrix represents a full covariance structure (dimension 216×3) that is shared among all four Gaussian components. The parcellation and regions are delineated according to the conventions specified in *S3 File*.

Fig G presents the correlation matrix derived from the Gaussian mixture model with four components. This figure emphasizes the high degree of similarity between the correlation submatrices for LFP envelopes, Glu_e, and $GABA_e$, supporting the assumption of synchronous activity patterns across these three variables. Moreover, the influence of the neuronal layer Ω_{Pyr} is visible across all correlation submatrices, validating the spatial consistency of the network dynamics. Interestingly, the anti-correlation patterns differentiate frontal–cingulate–insula from parietal–occipital–temporal lobes (which correspond to two communities within the neuronal layer Ω_{Pyr} as discussed in section *S4.3* in *S4 File*).
