# Supplementary material for: Dialogue mechanisms between astrocytic and neuronal networks: A whole-brain modelling approach
Source: PLoS Comput Biol. 2025 Jan 13;21(1):e1012683. doi: 10.1371/journal.pcbi.1012683 (PMC11730384; doi:10.1371/journal.pcbi.1012683)
Supplement: S6 File — (PDF) [file pcbi.1012683.s006.pdf]

Supporting Information for “Dialogue mechanisms between astrocytic and neuronal networks: a whole-brain modelling approach”

Obaï Bin Ka’b Ali[1,2,*], Alexandre Vidal[3], Christophe Grova[4,5], Habib Benali[2,6]

1. Physics Department, Concordia University, Montreal, Canada
2. Electrical and Computer Engineering Department, Concordia University, Montreal, Canada
3. Laboratoire de Mathématiques et Modélisation d’Evry (LAMME), Université Evry, CNRS, Université Paris-Saclay, France
4. Multimodal Functional Imaging Lab, Department of Physics, Concordia School of Health, Concordia University, Montreal, Canada
5. Multimodal Functional Imaging Lab, Biomedical Engineering Department, McGill University, Montreal, Canada
6. INSERM U1146, Paris, France

* Corresponding author: ali.obaibk@gmail.com

Table of Contents

S6: Neuron-astrocyte network connectivity analysis **2**

S6.1 Interconnections between global topological properties of multilayer functional networks and periodic orbit features of bifurcation diagrams 2

S6.2 Applying clustering analysis to explore patterns of global topological properties of multilayer functional networks 2

S6.3 Interplays between amplitude and phase couplings 3

S6.4 Structural reducibility analysis of multilayer functional networks 6

References **8**

List of Figures

Fig A. **Analysis of global topological properties of multilayer functional networks through bifurcation diagram features.** 2

Fig B. **Clustering analysis results of global topological properties of multilayer functional networks.** 3

Fig C. **Mean multilayer functional networks across four simulations.** 4

Fig D. **Structural reducibility metrics.** 6

Fig E. **Global topological properties of reduced multilayer functional networks.** 7

S6: Neuron-astrocyte network connectivity analysis

All graph theoretical analyses conducted in this study were facilitated by methodologies derived from *MuxVix* (De Domenico, Porter, et al., 2015), version 3.1 available at https://github.com/manlius/muxViz, and the *Brain Connectivity Toolbox*, 2019-03-03 release accessible at https://www.nitrc.org/projects/bct.

S6.1 Interconnections between global topological properties of multilayer functional networks and periodic orbit features of bifurcation diagrams

Fig A extends *Fig 8* of the *Main Manuscript* by introducing three additional properties of multilayer networks. The figure includes Von Neumann entropy (De Domenico, Nicosia, et al., 2015), which adapts Shannon information entropy to graph structures. It also features code length savings (Neuman et al., 2022), a relative measure assessing the efficiency of data compression achievable when a network is optimally organized into modules, as opposed to a single-module organization. Finally, the figure showcases modularity (Clauset et al., 2004), defined as the discrepancy between the fraction of within-community edges and the fraction expected by chance. Consistent with the observations in the *Main Manuscript*, these network property patterns closely align with periodic orbit E_{Pyr} peak–peak amplitude isolines.

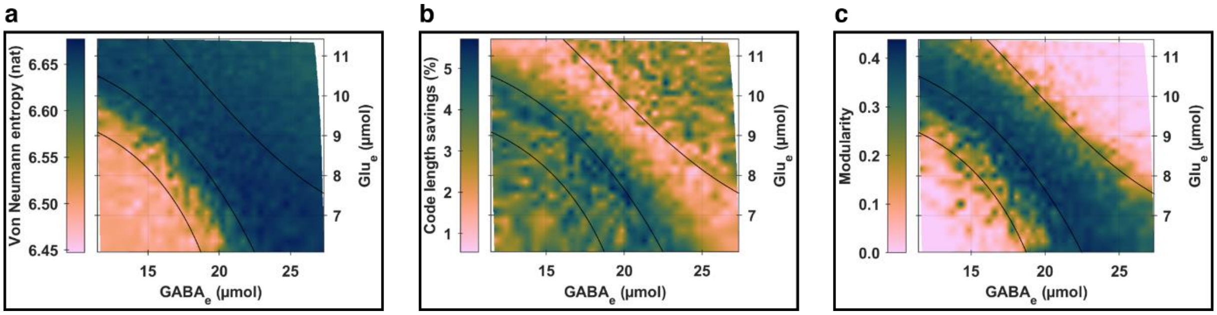

Fig A. **Analysis of global topological properties of multilayer functional networks through bifurcation diagram features. (a) Von Neumann entropy. (b) Code length savings. (c) Modularity. (a)–(c)** The black solid curves represent contour lines of periodic orbit E_{Pyr} peak–peak amplitudes, consistent with those in *Fig 6b* of the *Main Manuscript*. This alignment facilitates comparisons with clustering analysis results of global topological property patterns. Each isoline passes through specific $(\mathrm{Glu_e}; \mathrm{GABA_e})$ coordinates in (µmol) × (µmol): (8.5; 14.0), (8.50; 18.25), or (9.5; 21.0).

S6.2 Applying clustering analysis to explore patterns of global topological properties of multilayer functional networks

Fig B, extends *Fig 6b* of the *Main Manuscript* by exploring the impact of varying the number of components (four, five, or six) in Gaussian mixture models, with a focus on the comparison between networks analyzed over all ten simulation batches (totaling 10×1225 networks) and those subjected to structural reductions (10×1225 networks) or averaged across simulation batches (1225 networks). The analysis investigates clustering coefficient, path length, edge overlap, and code length, like in the *Main Manuscript*. The results revealed consistent clustering across different mixture models, with the cluster boundaries in reduced networks more accurately reflected by periodic orbit E_{InIn} peak–peak amplitude isolines rather than E_{Pyr} isolines. This underscores the interplay between excitatory and inhibitory dynamics in shaping different aspects of functional connectivity patterns. Additionally, the analysis of reduced multilayer networks confirmed that essential topological information was preserved despite reduction, ensuring that our findings are

largely unaffected by biases caused by redundant data (like between Glu_e-C and $GABA_e$-C layers). Similarly, analyzing averaged networks instead of the full dataset yielded comparable results, suggesting effective stochastic modeling. Importantly, all mixture models consistently pinpointed the critical isolines identified in *S5 File* as areas where network characteristics undergo profound transformations. This consistency reinforces the conclusions drawn in *S5 File*, particularly regarding the role of the cluster C2 delineated in Fig B(a).

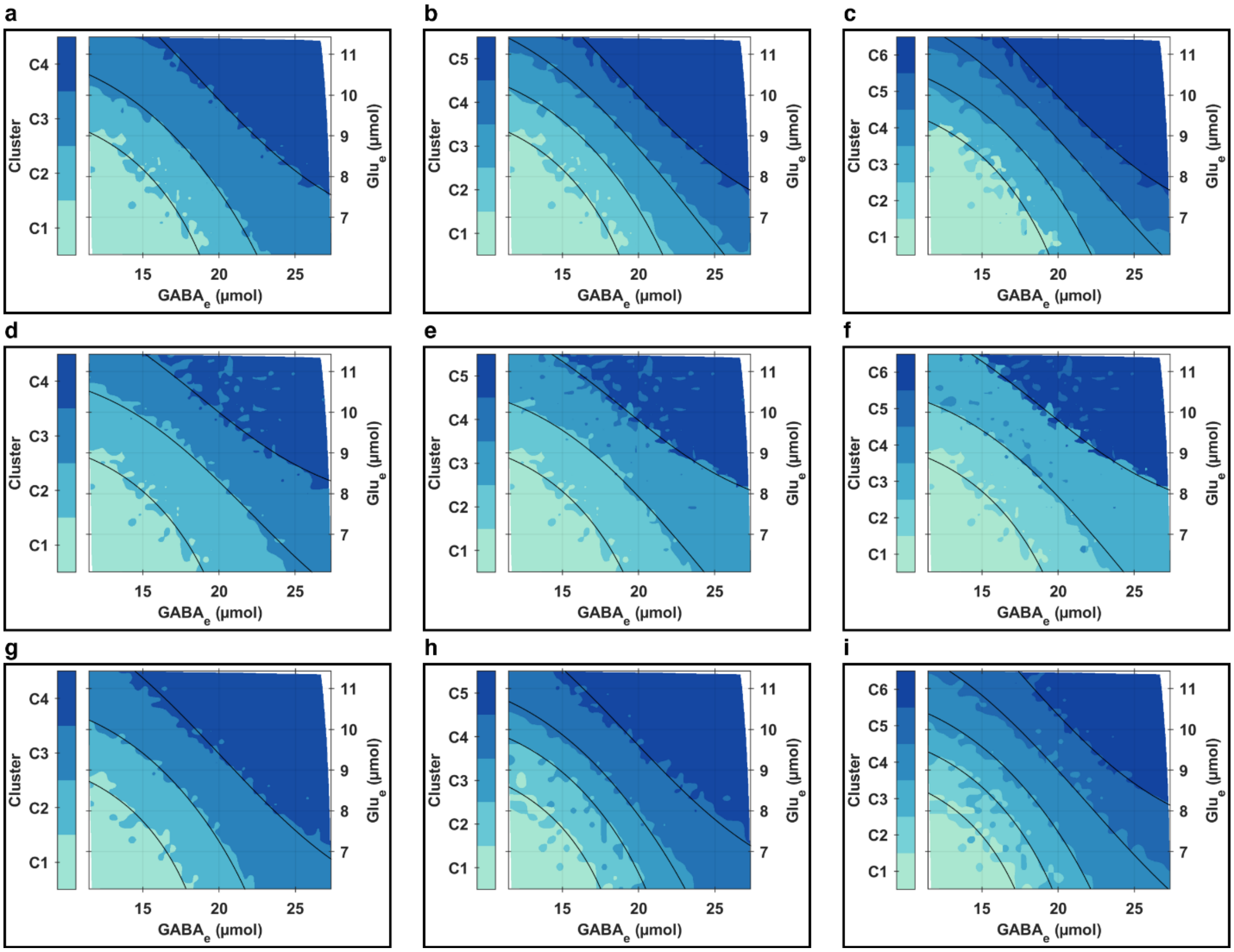

Fig B. **Clustering analysis results of global topological properties of multilayer functional networks.** This analysis examines clustering coefficient, path length, edge overlap, and code length, employing Gaussian mixture models with differing number of components across the three columns: four components in the first column **(a, d, g)**, five components in the second column **(b, e, h)**, and six components in the third column **(c, f, i)**. The first row panels **(a–c)** display results from analyzing all multilayer networks across the ten simulation batches, totaling 10×1225 networks. The second row panels **(d–f)** derive from a structural reducibility analysis of the same multilayer networks (10×1225 networks). The third row panels **(g–i)** show analyses of batch-averaged multilayer networks (1225 networks). The black solid curves in **(a–c)** and **(g–i)** depict periodic orbit E_{Pyr} peak–peak amplitude isolines, while those in **(d–f)** depict E_{InIn} isolines, with each isoline visually selected to align with cluster frontiers.

S6.3 Interplays between amplitude and phase couplings

Fig C, aligned with *Fig 9* of the *Main Manuscript*, analyses the mean multilayer networks derived from four simulations, by depicting their adjacency matrices, eigenvector versatilities, and community structures.

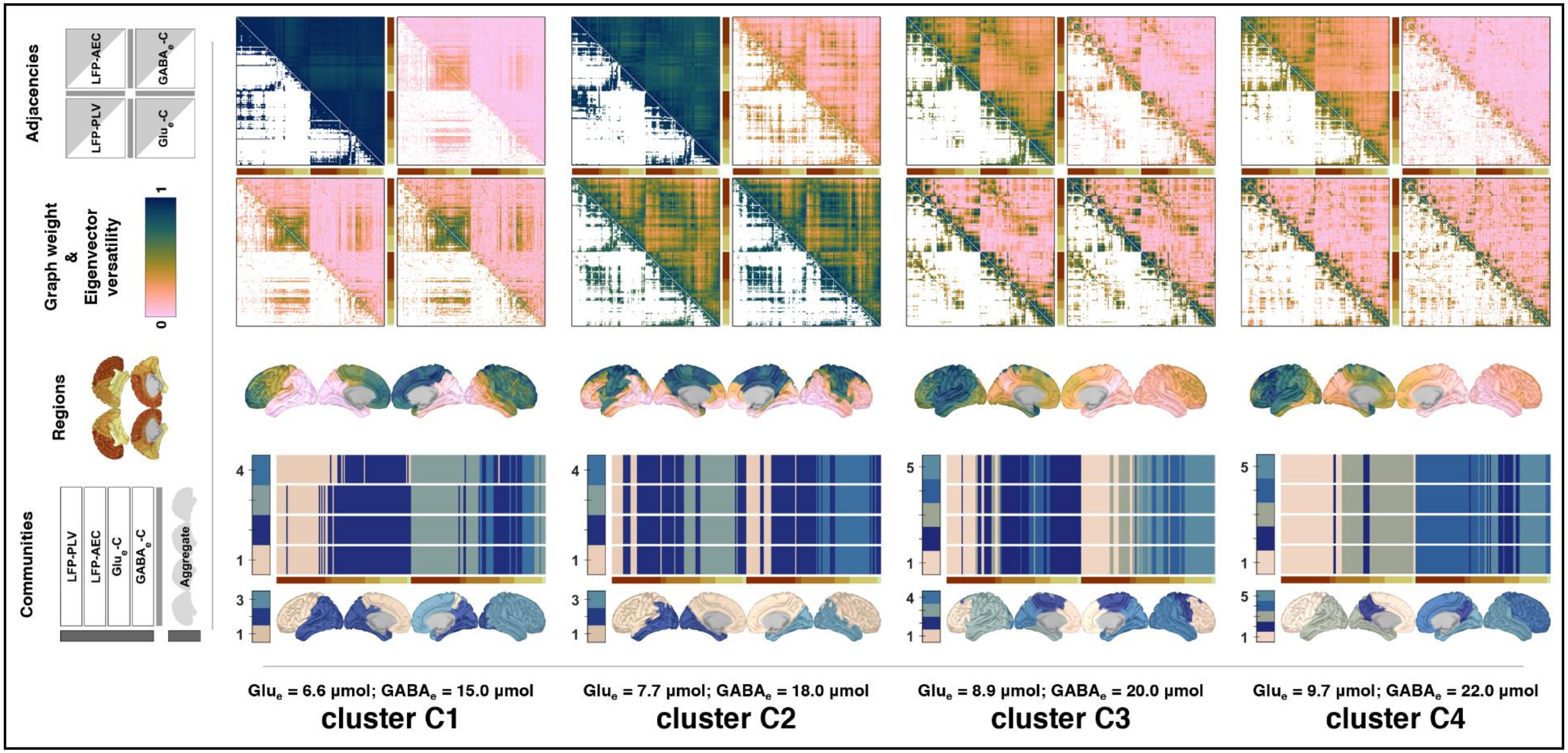

Fig C. **Mean multilayer functional networks across four simulations.** This figure displays the mean networks derived from four distinct simulations, each representing a unique cluster and identified by specific whole-brain neurotransmitter levels ($\mathrm{Glu_e}$; $\mathrm{GABA_e}$). The figure comprises three types of visual information. **Adjacency matrices**, located at the top of each column panel, these matrices represent four functional connectivity layers (LFP-PLV, LFP-AEC, $\mathrm{Glu_e}$-C, or $\mathrm{GABA_e}$-C) for each simulation. The legend on the top-left clarifies the type corresponding to each matrix. A thresholded version of each layer, retaining the top 25% of connectivity weights, is shown in the lower diagonal portions of the matrices. For simplicity and to focus on intra-layer connections, only the diagonal blocks of the rank-2 tensor representation of the multilayer networks are shown, each with a dimension of 216. This exclusion is noted, although it is important to mention that all inter-layer connections are identity matrices, forming a clique among the state nodes of a single physical node. **Eigenvector versatilities**, displayed in the middle of each column panel are brain maps that depict the nodal importance of each brain region. **Community structures**, presented at the bottom of each column panel are heatmaps and brain maps illustrating community profiles within each network. Two types of community profiles are shown for each simulation: one for the multilayer functional network and another for the aggregate functional network, as detailed in the legend schematic on the bottom-left. Communities are identified by color bars that specify the number and color of each community. **Adjacency matrices** and **Community structures**: The parcellation and regional definitions used in the adjacency matrices and community profiles are delineated according to the conventions specified in *S3 File*.

The comparative study of the adjacency matrices revealed that while the Glu_e-C and $GABA_e$-C layers exhibited a high degree of spatial similarity, their edge weight distributions had slightly different means across the parameter plane. These layers were similarly patterned to the LFP-AEC layers, although the LFP-AEC layers exhibited more narrowly concentrated weight distributions around the mean. This suggests a more pronounced central tendency in the LFP-AEC layers, which also featured the smallest mean values among the layers studied. In contrast, LFP-PLV layers stood out for their distinct topologies, differing considerably from the correlation layers. These differences underscore the potential complementary functional roles of amplitude and phase network synchrony. For instance, in the simulation of cluster C1 (more precisely, near the *critical* boundary between cluster C1 and cluster C2) as depicted in Fig C, the parietal–occipital–temporal regions of the left hemisphere and the occipital regions of the right hemisphere (which were associated with the highest levels of Glu_e and $GABA_e$, where periodic orbit peak–peak amplitudes are notably larger), display strong amplitude synchrony but variable phase relationships. In contrast, regions in the remaining lobes (characterized by the lowest Glu_e and $GABA_e$ levels) maintain consistent phase relationships but varied amplitude synchrony. However, in the simulations of clusters C3 and C4, also shown in Fig C, amplitude and phase synchrony exhibit similar topological features. Notably, the LFP-PLV layers maintained remarkably stable connectivity patterns throughout all simulations, showcasing a network backbone that mirrors the neuronal layer Ω_{Pyr} discussed in section *S4.3* in *S4 File*. This backbone of the LFP-PLV layers connected densely interlinked regions, particularly distinguishing the frontal–cingulate lobes where connections were predominantly inter-hemispheric, from other brain regions where connections tended to be intra-hemispheric. These consistent connectivity patterns were not as prevalent in other layers, which only showed similar connectivity patterns near and above the boundary between cluster C2 and cluster C3.

Additionally, the multilayer community detections revealed functional modules primarily comprising frontal–cingulate–insula–parietal regions, juxtaposed with parietal–occipital–temporal regions, with a pronounced preference for intra-hemispheric organization. This organizational pattern mirrors the community structures observed within both the astrocytic and neuronal layers, as previously discussed in section *S4.3* in *S4 File*, with the astrocytic structural constraints particularly facilitating intra-hemispheric or short-range couplings. While not presented here, our detailed examination of adjacency matrices and multilayer community profiles indicated that modules spanning both hemispheres often emerged under conditions where frontal inter-hemispheric connections were weakened, especially in areas of the parameter plane coinciding with the cluster C2 from Fig B(a) where network integration and segregation levels reached their peak. These findings hint at a possible interplay between sparse inter-hemispheric frontal connectivity and the network model's ability to manifest *critical* dynamics, while potentially fostering the establishment of diverse cross-hemispheric links. Moreover, the analysis of LFP-PLV layers indicated slight differences in community structures compared to the correlation layers, and examining the community profiles in the aggregate functional networks demonstrated the enhanced utility of a multilayer modeling approach to functional connectivity, compared to a more simplified aggregated view.

Lastly, the mapping of eigenvector versatility corroborated the preceding findings, highlighting the distinctions in centrality between the hemispheres and among major regional clusters. Detailed analyses revealed consistent centrality in the frontal regions, with a notable transition in the least

central nodes from parietal to occipital to temporal regions. This gradient in functional patterns underscores the intricate posterior–anterior information flows within the network dynamics.

As we close this section *S6.3*, a discussion of its limitations and prospective advancements is essential. In this study, we have presented a simplified depiction of network dynamics by selecting a representative mean multilayer network for each cluster in Fig C (or *Fig 9* of the *Main Manuscript*), instead of displaying all networks from the ten simulation batches per cluster. This approach enhances clarity but may not fully capture the variability of the networks, a concern amplified by the influence of white noise on the *critical* dynamics observed in our simulations. While not depicted here, supplementary analyses employing consensus methods for eigenvector versatility and community detection have corroborated our findings. These techniques help bridge the gap between analyzing an average network representation and examining multiple networks through consensus approaches. Additionally, our adoption of a multiplex network modeling perspective was driven by the lack of standardized methods for defining inter-layer connectivity in more complex multilayer frameworks. Although outside our current analysis scope, previous studies have explored various connectivity measures such as amplitude–amplitude, phase–phase, and amplitude–phase interactions (Brookes et al., 2016; De Domenico, 2017; Palva et al., 2018; Sadaghiani et al., 2022; Tewarie et al., 2016), highlighting the complexity and potential of these measures to reveal unique network topologies not evident through linear correlations and phase locking values alone. Moreover, substantial theoretical challenges arise when applying multilayer functional network models to real-world data. Developing appropriate null models to prevent the mapping of spurious connections and effectively quantifying topological descriptors under stochastic conditions remain formidable challenges (De Domenico, 2017; Raimondo & De Domenico, 2021). Overcoming these obstacles is essential for advancing our understanding of complex network dynamics. Looking forward, it would be beneficial for future research to explore time-resolved and dynamic functional connectivity analyses (Heitmann & Breakspear, 2018; Preti et al., 2017). Such approaches align with our network model's sensitivity to non-stationarity over brief time intervals and would enable more precise characterization of state transitions occurring across different temporal scales, transitions that might be missed by static analyses.

S6.4 Structural reducibility analysis of multilayer functional networks

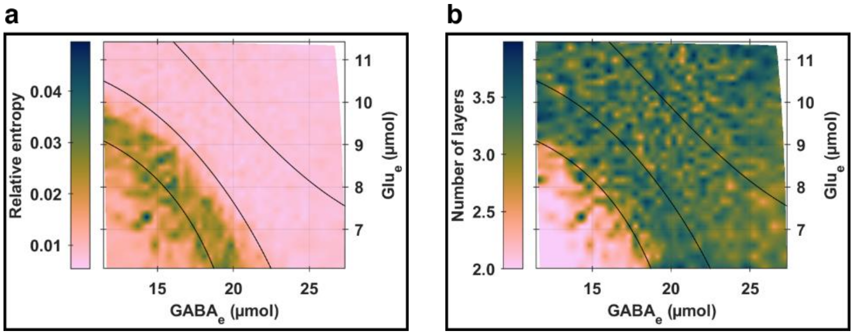

Fig D. **Structural reducibility metrics.** Panel **(a)** displays relative entropies, which measure the level of distinguishability of an optimally reduced multilayer network from its aggregated counterpart, with higher values indicating less redundant topological information across layers. Panel **(b)** shows the number of layers retained after reducibility operations, determined by averaging across multiple simulation batches. The black solid curves represent contour lines of periodic orbit E_{Pyr} peak–peak amplitudes, consistent with those in *Fig 6b* of the *Main Manuscript*. This alignment facilitates comparisons with clustering analysis results of global topological property patterns. Each isoline passes through specific $(\mathrm{Glu_e}; \mathrm{GABA_e})$ coordinates in (μmol) × (μmol): (8.5; 14.0), (8.50; 18.25), or (9.5; 21.0).

Fig D(a) displays relative entropies, assessing the level to which a network can be distinguished from its aggregate counterpart. Notably, the local maxima of these patterns coincide with the cluster C2 identified in Fig B(a). Further exploring in Fig D(b) presents the average number of layers retained after reducibility operations. These visuals reinforce discussions from the *Main Manuscript* concerning the outcomes of structural reducibility. They highlight that many simulations retained all layers without merging, indicative of a lack of redundant topological information and thus maintaining approximately four layers. Additionally. they confirm that in configurations where the network model exhibited *homogeneous* behaviors, as detailed in section *S5.2* in *S5 File*, specifically below the first contour line, three out of four layers (essentially all correlation layers) were merged due to redundancy. The analysis of Fig D also reveals that the simulations associated with cluster C2 uniquely featured multilayer networks that were maximally distinguishable from their aggregate counterparts and devoid of redundant layers, eliminating the need for merging. This observation supports the anticipation of *critical* dynamical network behaviors within cluster C2, as extensively discussed throughout *S5 File*.

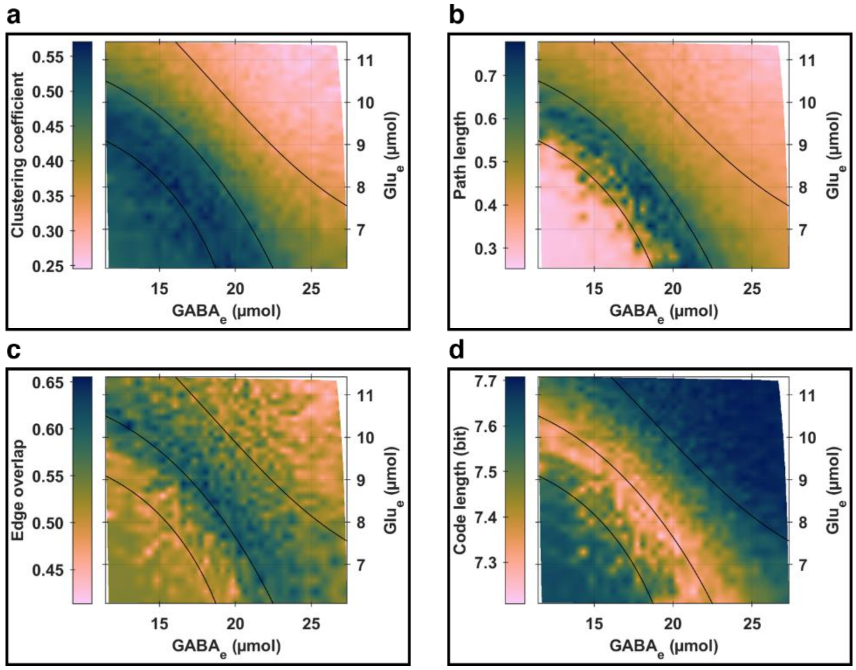

Fig E. **Global topological properties of reduced multilayer functional networks. (a) Clustering coefficient. (b) Path length. (c) Edge overlap. (d) Code length. (a)–(d)** The black solid curves represent contour lines of periodic orbit E_{Pyr} peak–peak amplitudes, consistent with those in *Fig 6b* of the *Main Manuscript*. This alignment facilitates comparisons with clustering analysis results of global topological property patterns. Each isoline passes through specific $(\text{Glu}_\text{e}; \text{GABA}_\text{e})$ coordinates in (μmol) × (μmol): (8.5; 14.0), (8.50; 18.25), or (9.5; 21.0).

Fig E extends *Fig 8* from the *Main Manuscript* by comparing the global topological properties of networks after reducibility operations with those of the original networks. The high consistency in global topological property patterns between the reduced and original networks aligns with the clustering interpretations provided in the *Main Manuscript* and further explored in section *S6.2*. This consistency underscores the efficacy of the reducibility operations in preserving essential topological features.

References

Brookes, M. J., Tewarie, P. K., Hunt, B. A. E., Robson, S. E., Gascoyne, L. E., Liddle, E. B., Liddle, P. F., & Morris, P. G. (2016). A multi-layer network approach to MEG connectivity analysis. *NeuroImage*, *132*, 425–438. https://doi.org/10.1016/j.neuroimage.2016.02.045

Clauset, A., Newman, M. E. J., & Moore, C. (2004). Finding community structure in very large networks. *Physical Review E - Statistical Physics, Plasmas, Fluids, and Related Interdisciplinary Topics*, *70*(6), 6. https://doi.org/10.1103/PhysRevE.70.066111

De Domenico, M. (2017). Multilayer modeling and analysis of human brain networks. *GigaScience*, *6*(5), 1–8. https://doi.org/10.1093/gigascience/gix004

De Domenico, M., Nicosia, V., Arenas, A., & Latora, V. (2015). Structural reducibility of multilayer networks. *Nature Communications*, *6*(1), 6864. https://doi.org/10.1038/ncomms7864

De Domenico, M., Porter, M. A., & Arenas, A. (2015). MuxViz: a tool for multilayer analysis and visualization of networks. *Journal of Complex Networks*, *3*(2), 159–176. https://doi.org/10.1093/comnet/cnu038

Heitmann, S., & Breakspear, M. (2018). Putting the "dynamic" back into dynamic functional connectivity. *Network Neuroscience*, *2*(2), 150–174. https://doi.org/10.1162/netn_a_00041

Neuman, M., Jonsson, V., Calatayud, J., & Rosvall, M. (2022). Cross-validation of correlation networks using modular structure. *Applied Network Science*, *7*(1). https://doi.org/10.1007/s41109-022-00516-5

Palva, J. M., Wang, S. H., Palva, S., Zhigalov, A., Monto, S., Brookes, M. J., Schoffelen, J.-M., & Jerbi, K. (2018). Ghost interactions in MEG/EEG source space: A note of caution on inter-areal coupling measures. *NeuroImage*, *173*(February), 632–643. https://doi.org/10.1016/j.neuroimage.2018.02.032

Preti, M. G., Bolton, T. A., & Van De Ville, D. (2017). The dynamic functional connectome: State-of-the-art and perspectives. *NeuroImage*, *160*(December 2016), 41–54. https://doi.org/10.1016/j.neuroimage.2016.12.061

Raimondo, S., & De Domenico, M. (2021). Measuring topological descriptors of complex networks under uncertainty. *Physical Review E*, *103*(2), 1–15. https://doi.org/10.1103/PhysRevE.103.022311

Sadaghiani, S., Brookes, M. J., & Baillet, S. (2022). Connectomics of human electrophysiology. *NeuroImage*, *247*(December 2021), 118788. https://doi.org/10.1016/j.neuroimage.2021.118788

Tewarie, P., Hillebrand, A., van Dijk, B. W., Stam, C. J., O'Neill, G. C., Van Mieghem, P., Meier, J. M., Woolrich, M. W., Morris, P. G., & Brookes, M. J. (2016). Integrating cross-frequency and within band functional networks in resting-state MEG: A multi-layer network approach. *NeuroImage*, *142*, 324–336. https://doi.org/10.1016/j.neuroimage.2016.07.057
